# Electron transfer ferredoxins with unusual cluster binding motifs support secondary metabolism in many bacteria†
†Electronic supplementary information (ESI) available: Details of *M. marinum* CYPome, experimental methods and additional data tables and figures. See DOI: 10.1039/c8sc01286e


**DOI:** 10.1039/c8sc01286e

**Published:** 2018-08-23

**Authors:** Stella A. Child, Justin M. Bradley, Tara L. Pukala, Dimitri A. Svistunenko, Nick E. Le Brun, Stephen G. Bell

**Affiliations:** a Department of Chemistry , University of Adelaide , SA 5005 , Australia . Email: stephen.bell@adelaide.edu.au; b Centre for Molecular and Structural Biochemistry , School of Chemistry , University of East Anglia , Norwich Research Park , Norwich , NR4 7TJ , UK; c School of Biological Sciences , University of Essex , Wivenhoe Park , Colchester CO4 3SQ , UK

## Abstract

The proteins responsible for controlling electron transfer in bacterial secondary metabolism are not always known or characterised. Here we demonstrate that many bacteria contain a set of unfamiliar ferredoxin encoding genes which are associated with those of cytochrome P450 (CYP) monooxygenases and as such are involved in anabolic and catabolic metabolism. The model organism *Mycobacterium marinum* M contains eleven of these genes which encode [3Fe–4S] or [4Fe–4S] single cluster containing ferredoxins but which have unusual iron–sulfur cluster binding motif sequences, **C**XX**?**XX**C**(X)_*n*_**C**P, where ‘**?**’ indicates a variable amino acid residue. Rather than a cysteine residue, which is highly conserved in [4Fe–4S] clusters, or alanine or glycine residues, which are common in [3Fe–4S] ferredoxins, these genes encode at this position histidine, asparagine, tyrosine, serine, threonine or phenylalanine. We have purified, characterised and reconstituted the activity of several of these CYP/electron transfer partner systems and show that all those examined contain a [3Fe–4S] cluster. Furthermore, the ferredoxin used and the identity of the variable motif residue in these proteins affects the functionality of the monooxygenase system and has a significant influence on the redox properties of the ferredoxins. Similar ferredoxin encoding genes were identified across *Mycobacterium* species, including in the pathogenic *M. tuberculosis* and *M. ulcerans*, as well as in a wide range of other bacteria such as *Rhodococcus* and *Streptomyces*. In the majority of instances these are associated with CYP genes. These ferredoxin systems are important in controlling electron transfer across bacterial secondary metabolite production processes which include antibiotic and pigment formation among others.

## Abstract 2

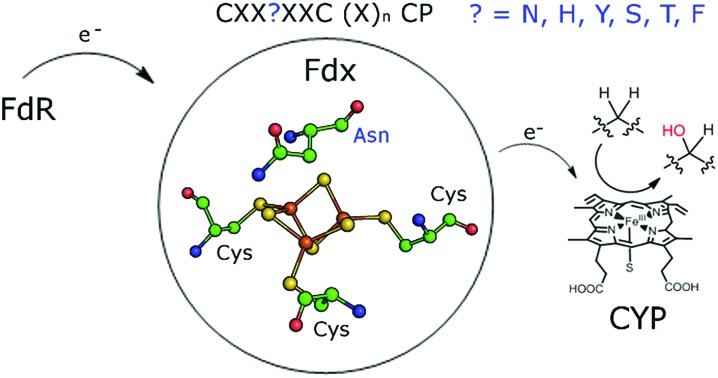
Unusual ferredoxins with different iron–sulfur cluster binding motifs support natural product biosynthesis in a wide range of bacteria.

## Introduction

Electron transfer is fundamental for all living organisms, being involved in respiratory processes to produce chemical energy within the cell, pathways to build large molecules from smaller substrates (anabolic) and the breakdown of molecules into smaller species for cellular metabolism (catabolic). The electron carrier proteins involved in these pathways tightly regulate the shuttling of electrons between the donor and acceptor.^1^,^2^ Monooxygenases are an essential set of enzymes that are intrinsically involved in these anabolic and catabolic processes and require a supply of electrons in order to function. They typically catalyse the selective hydroxylation of a wide range of organic molecules using dioxygen (eqn (1)).^3^,^4^

1 R–H + 2H^+^ + 2e^–^ + O_2_ → R–OH + H_2_O

In Nature, monooxygenase enzymes, including cytochrome P450 enzymes (CYP), show exquisite selectivity. The bacterial CYP family has a broad substrate spectrum yet each individual enzyme can be highly specific. For these reasons many bacteria, including *Mycobacteria*, have a large and highly evolved CYP complement (CYPome) which functions to break down or synthesise molecules as required by the organism.^5^ These enzymes are a valuable resource for biocatalysis and key targets for antibacterial drug design.^6^–^8^

The two electrons required by CYP enzymes are usually derived from NAD(P)H and delivered by electron transfer proteins in two distinct steps. Bacterial CYP enzymes typically use Class I electron transfer systems which normally consist of a NAD(P)H-dependent ferredoxin reductase (FdR) and an iron–sulfur ferredoxin (Fdx).^9^ The reductase most often contains an FAD cofactor. The best studied ferredoxins in these systems are [2Fe–2S] types but [3Fe–4S], [4Fe–4S] clusters, combinations thereof and exceptions such as the FMN containing cindoxin and self-sufficient P450Bm3 are known.^7^,^9^–^12^ The CYP enzymes have evolved to be highly specific for their electron transfer partners from the same species.^13^ Yet within a given bacterium the electron transfer systems can support multiple CYP enzymes and accordingly the number of genes in a bacterium normally decreases in the order CYP > ferredoxin > ferredoxin reductase.^14^–^17^

One bacterium which contains many of these systems is *Mycobacterium marinum*, a ubiquitous pathogen of fish and amphibians, which can infect humans causing aquarium granuloma.^20^,^21^ *M. marinum* can survive in extracellular environmental conditions and is used as a model organism to plot the evolutionary pathway of other more specialist *Mycobacterium* pathogens.^22^ It is closely related to the slower growing human pathogens *Mycobacterium ulcerans* (97% nucleotide sequence identity) and *Mycobacterium tuberculosis* (85%) which cause Buruli ulcer (Bairnsdale or Daintree ulcer) and tuberculosis, respectively.^20^–^23^ Tuberculosis is a global epidemic and a major cause of human mortality and is a growing problem due to the evolution of drug resistant strains.^24^,^25^ Buruli ulcer is a serious skin disease prevalent in Africa, Oceania and Asia.^26^ The mycobacterial pathogens that cause these more serious conditions have adapted to the specific host environment in which they are found and are not viable in other settings. In comparison to *M. marinum*, both *M. tuberculosis* and *M. ulcerans* have undergone reductive evolution. This occurred *via* genome downsizing and pseudogene formation, through the introduction of mutations or insertion sequences.^27^–^29^ Another human pathogen, *Mycobacterium leprae*, responsible for leprosy, has undergone extreme reductive evolution and contains less than half the number of genes of *M. tuberculosis*. In addition these bacteria have also acquired certain unique genes which encode proteins critical to their survival.^23^,^29^

Here we report that there are substantially more CYP encoding genes in *M. marinum* than in *M. ulcerans* and *M. tuberculosis*. The genome of *M. marinum* also contains many genes which encode atypical ferredoxins. Most of these are located close to CYP genes and the majority are [3/4Fe–4S] type ferredoxins containing unusual residues in the iron–sulfur cluster binding motif. Rather than having the **C**XX**C**XX**C**(X)_*n*_**C**P motif of a [4Fe–4S] cluster ferredoxin or the **C**XX**A**/**G**XX**C**(X)_*n*_**C**P motif of a [3Fe–4S] cluster ferredoxin, a different amino acid such as histidine replaces the second cysteine of the [4Fe–4S] motif or the glycine/alanine residue in the [3Fe–4S] ferredoxin motif (**C**XX**H**XX**C**(X)_*n*_**C**P).^18^ The majority of these have yet to be studied and characterised.^18^,^19^

By searching for similar electron transfer systems we find that other residues are commonly located at this position (**C**XX**?**XX**C**(X)_*n*_**C**P) in ferredoxins of this type, from other bacteria.^15^,^30^–^34^ For example, the ferredoxin genes of *M. marinum* encode proteins which contain histidine, asparagine, serine, threonine, tyrosine and phenylalanine. We identify that these types of ferredoxin genes are prevalent across many other bacterial species and isolate and characterise several of the *M. marinum* suite. We demonstrate that, in combination with the same ferredoxin reductase, they can support the monooxygenase activity of their associated CYP enzymes. We characterise the cluster type and importance of the variable residue by a variety of methods and show that they significantly alter the properties of the iron–sulfur cluster. We also demonstrate that CYP enzyme activity is dependent on choosing the correct ferredoxin. Therefore the ferredoxins which contain these unusual residues have a critical function in the metabolism of *M. marinum* and other bacteria through their support of electron transfer to monooxygenase systems.

## Results

### Analysis of the CYPome and the potential electron transfer partners of *M. marinum*

There are forty seven CYP enzyme encoding genes in *M. marinum* and these belong to thirty six different P450 families and thirty nine subfamilies (see ESI for further details; Tables S1, S2 and Fig. S1†). Comparatively there are only twenty CYP enzymes in *M. tuberculosis* and twenty four in *M. ulcerans*. In contrast to these species, *M. leprae* contains only one CYP enzyme encoding gene, CYP164A1. This follows from the smaller gene complement of these bacteria due to reductive evolution (Tables S1 and S3†).^35^–^37^ The CYPome of *M. marinum* plays a role in the bacterium's anabolic and catabolic metabolism. The substrate profiles of several of the cytochrome P450 enzymes can be inferred from sequence homology to those that have been previously studied in other mycobacterial species and inferred from sequence homology (see ESI†).^38^–^43^

**Fig. 1:**
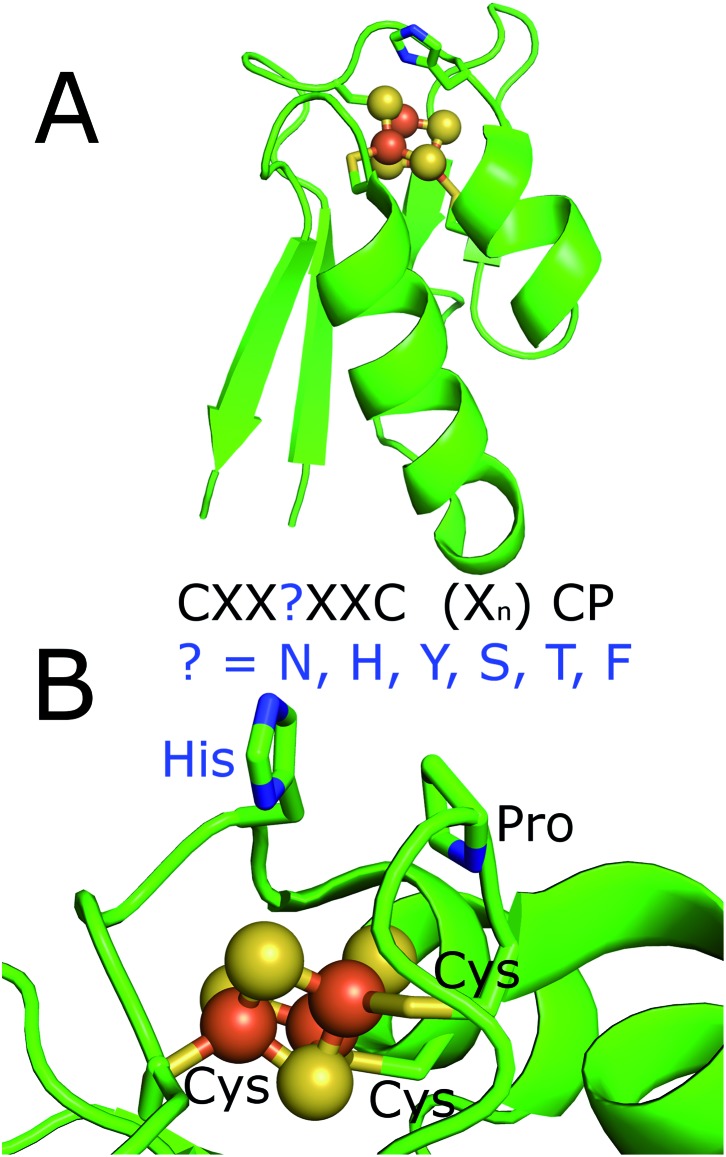
The X-ray crystal structure of the histidine containing ferredoxins from *R. palustris* HaA2 (PDB ; 4ID8).^18^ (A) Full structure; (B) a zoomed in view of the iron–sulfur cluster with the residues of the ferredoxin binding motif highlighted. The motif of the ferredoxins from *M. marinum* is highlighted. These residues would replace the histidine which is highlighted in the figure.

There are many ferredoxin genes associated with the CYPome of *M. marinum*. Overall *M. marinum* contains a set of eleven genes encoding ferredoxins of the single iron–sulfur cluster [3/4Fe–4S] type (Fdx1–Fdx11). These contain the motif **C**XX**?**XX**C**(X)_*n*_**C**P without a cysteine at the ‘**?**’ position (Fig. 1, S2†). These include the equivalent ferredoxins to Rv0763 and Rv1786 of *M. tuberculosis*, (Fdx1 and Fdx10 in *M. marinum*; **C**XX**H**XX**C**(X)_*n*_**C**P, Table 1). These are the only electron transfer partner genes from the CYPome of *M. tuberculosis* (ferredoxin and ferredoxin-NAD(P)H reductase-like proteins) located in the vicinity of the CYP genes (Tables S4 and S5†).^5^,^30^,^44^–^46^ In both *Mycobacterium* species these two ferredoxins are located next to the genes of CYP51 and CYP143 (Tables 1, S3, Fig. S3†). Equivalent genes to all of the electron transfer partners of *M. tuberculosis* are also present in the genome of *M. marinum* (Table S5†). The ferredoxins, Fdx5 and Fdx8, from *M. marinum*, also contain a histidine residue at the variable position.

**Table 1:** The potential electron transfer partners of the CYPome of *M. marinum* (which are located close to CYP genes). The gene name as per the databases at the National Center for Biotechnology Information is provided. The sequence of the iron–sulfur cluster binding motif of the [3/4Fe–4S] ferredoxins as well as the predicted pI and length of the amino acid chain are provided. The equivalent ferredoxin genes in *M. ulcerans* and *M. tuberculosis* are given. The neighbouring CYP genes (1–4 genes away) are also shown (Table S1)

Gene name	ID	Iron–sulfur cluster binding motif	pI	AA	*M. ul*	*M. tb*	*CYP* enzyme
Mmar_2667	Fdx1	**C**XX**H**XX**C**(X)_*n*_**C**P	3.9	63	Mul_5	Rv1786	CYP143A4
Mmar_2879	Fdx2	**C**XX**T**XX**C**(X)_*n*_**C**P	4.0	63	Mul_4	—	CYP278A1
Mmar_2932	Fdx3	**C**XX**Y**XX**C**(X)_*n*_**C**P	4.3	70	—	—	CYP147G1
Mmar_3973	Fdx4	**C**XX**N**XX**C**(X)_*n*_**C**P	3.7	62	Mul_6	—	CYP269A1 & CYP138A4
Mmar_4716	Fdx5	**C**XX**H**XX**C**(X)_*n*_**C**P	3.7	65	Mul_1	—	CYP188A3
Mmar_4730	Fdx6	**C**XX**F**XX**C**(X)_*n*_**C**P	4.3	97	—	—	CYP190A3
Mmar_4734	Fdx7	**C**XX**N**XX**C**(X)_*n*_**C**P	3.8	62	—	—	CYP190A3 & CYP150A5
Mmar_4736	Fdx8	**C**XX**H**XX**C**(X)_*n*_**C**P	4.0	61	—	—	CYP150A5
Mmar_4763	Fdx9	**C**XX**S**XX**C**(X)_*n*_**C**P	3.9	63	Mul_2	—	CYP105Q4
Mmar_4933	Fdx10	**C**XX**H**XX**C**(X)_*n*_**C**P	4.4	67	Mul_3	Rv0763	CYP51B1
Mmar_4991	Fdx11	**C**XX**N**XX**C**(X)_*n*_**C**P	3.9	81	Mul_7	Rv3503c	—
Mmar_3155	[2Fe–2S]		4.0	105	—	—	CYP153A16
Mmar_2931	FdR1	n.a.	5.8	466	—	—	CYP147G1
Mmar_3153	FdR2	n.a.	4.9	400	—	—	CYP153A16

The gene for the CYP153A16 enzyme (Mmar_3154) is in a cluster with genes encoding a [2Fe–2S] ferredoxin (Mmar_3155) and a ferredoxin reductase (FdR2, Mmar_3153), which completes a class I electron transfer system in *M. marinum* (FdR2/[2Fe–2S] ferredoxin/CYP153A16).^43^ A second gene encoding the CYP147G1 (Mmar_2930) enzyme is in a cluster with genes encoding a ferredoxin reductase (FdR1, Mmar_2931) and a ferredoxin (Fdx3, Mmar_2932). There are therefore two obvious complete electron transfer systems in *M. marinum* (FdR1/Fdx3/CYP147G1 and FdR2/[2Fe–2S] ferredoxin/CYP153A16). By analogy with other bacterial systems these two ferredoxin reductases are likely to be responsible for the reduction of ferredoxins and therefore support the CYP enzymes in *M. marinum*.^11^,^13^,^47^,^48^ Fdx3, has a tyrosine in its cluster binding motif (**C**XX**Y**XX**C**(X)_*n*_**C**P). The other ferredoxins of *M. marinum* contain asparagine (Fdx4, Fdx7 and Fdx11), serine (Fdx9), threonine (Fdx2) or phenylalanine (Fdx6) residues at this position of the cluster binding motif (Fig. 1, Table 1, Fig. S2†). All bar phenylalanine could potentially act as a ligand to a metal ion in the cluster.

The *M. marinum* ferredoxins of this type range in size from 62 to 97 amino acid residues in length and all have predicted pI values lower than 7.0 (Table 1). Ten of the potential ferredoxins of *M. marinum*, Fdx11 being the exception, are located next to or close to a CYP gene (one to four genes away, Fig. S3†). Therefore these ferredoxins are likely electron transfer partners which deliver reducing equivalents to the CYP enzymes.^33^

**Fig. 2:**
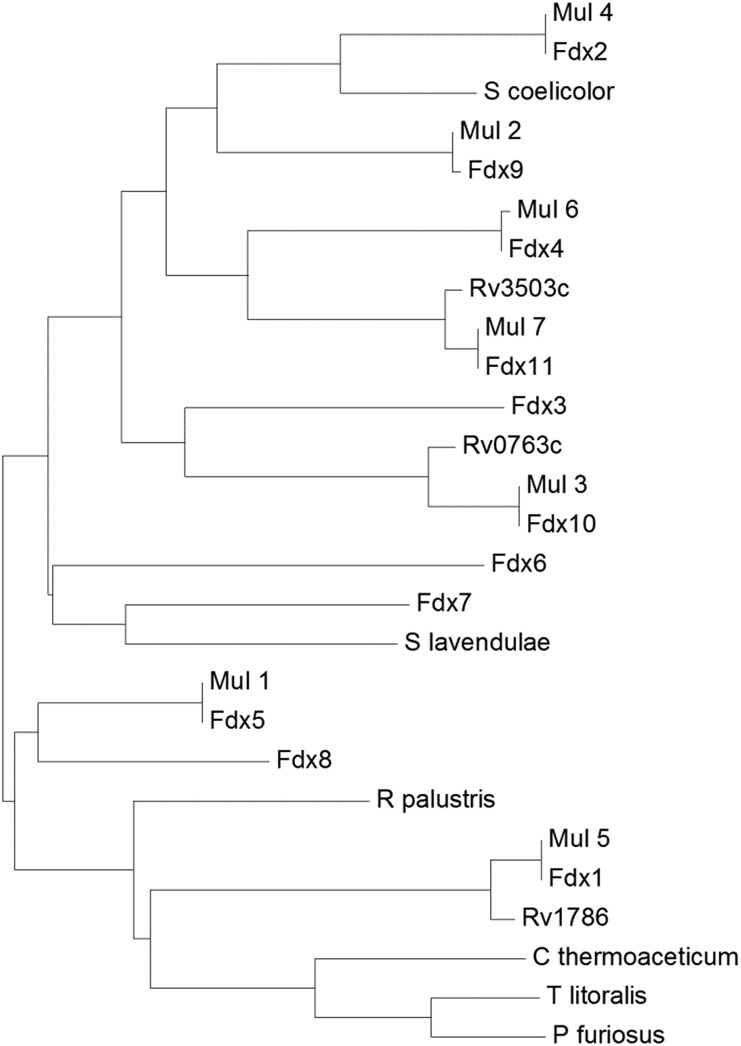
A phylogenetic tree (phenogram) of the [3/4Fe–4S] ferredoxins from *M. marinum* (Fdx1–Fdx11), *M. ulcerans* (Mul_1–Mul_7), and *M. tuberculosis* (Rv0763c, Rv1786 and Rv3503c). The ferredoxins from *S. coelicolor*, *S. lavendulae* and the structurally characterised ferredoxins from *R. palustris* HaA2, *P. furiosus*, *C. thermoaceticum* and *T. litoralis* are included for comparison (see figure). The grouping of the ferredoxins from *M. marinum* and *M. ulcerans* show they are closely related (97–100% sequence identity). There is a lower yet significant similarity to the ferredoxins from *M. tuberculosis* (78–92% sequence identity; note the low 78% value is unusual and arises as the gene Rv3503c is shorter than Fdx11 by the equivalent of nineteen amino acids). For the majority of the ferredoxins there is a low similarity to those from other bacterial species, for example Fdx1 has only 35% sequence identity with the structurally characterised *R. palustris* HaA2 ferredoxin (PDB: ; 4ID8). The threonine containing Fdx2 has the closest relationship with the [3Fe–4S] ferredoxins from *Streptomyces* species while the [4Fe–4S] ferredoxins from the thermophiles *P. furiosus*, *C. thermoaceticum* and *T. litoralis* cluster together.^11^,^14^,^49^–^51^

Of these eleven ferredoxin genes, seven are conserved in *M. ulcerans* and only three in *M. tuberculosis* (Table 1 and Fig. 2).^22^,^24^,^26^ The ferredoxins which are conserved in both *M. marinum* and *M. ulcerans* are very similar or identical showing 97–100% sequence identity, while those of *M. tuberculosis* show more sequence divergence (78–92% identity, Fig. 2). Neither of the ferredoxin reductase genes (FdR1 and FdR2) are conserved in *M. ulcerans* or *M. tuberculosis*. Extending the search to include more diverse species of Mycobacteria and other metabolically varied bacteria revealed the prevalence of analogous ferredoxins (Tables S6 & S7, Fig. S3†).

With the exception of the threonine containing Fdx2, many equivalent ferredoxin genes are found across the *Mycobacterium* genus. These are, more often than not, located in gene clusters with associated CYP genes (Table S6, Fig. S3†). They are also found across other bacteria, including the antibiotic synthesising *Streptomyces* (where variants in the motif at ‘**?**’ include histidine, serine and tyrosine; Table S7†) and *Rhodococcus* (where variants in the motif at ‘**?**’ include histidine, asparagine and tyrosine; Table S7†).^14^ They are also associated with the CYPome of other diverse bacterial species including those of *R. palustris* strains (Table S7†).

To provide insight into the important CYP enzyme catalysed reactions that these ferredoxins support we analysed biosynthetic gene cluster databases for these types of electron transfer partners (Table S8†). The synthesis of a range of complex secondary metabolites, including salinomycin, filipin and cinnabaramide, was found to be supported by these CYP enzyme and ferredoxin combinations. These were mainly found in strains of *Streptomyces* bacteria (Table S8†). This gives a small snapshot of the widespread and critical role these types of ferredoxins have in bacterial metabolism.

### Reconstitution of the activity of CYP147G1 using FdR1 and Fdx3

We set to out to determine whether these electron transfer partners could support the P450 enzymes of *M. marinum* and if the activity would be dependent on using the physiological ferredoxin electron transfer partners. Fdx3 (Mmar_2932) is the only ferredoxin of this type from *M. marinum* to have both a reductase gene (FdR1, Mmar_2931) as well as a CYP gene (CYP147G1, Mmar_2930) co-located in the genome and this was an obvious system to start with (Table S9†).

In order to assess if the ferredoxin reductase (FdR1) and ferredoxin (Fdx3) electron transfer proteins could support the activity of the CYP147G1 enzyme, we constructed a whole-cell oxidation system.^33^,^52^ The FdR1 and Fdx3 genes were cloned in pETDuet to generate pETDuetFdx3/FdR1 and the CYP147G1 and Fdx3 genes were combined with pRSFDuet to construct pRSFDuetFdx3/CYP147G1. By transforming both vectors into *E. coli* we were able to produce all three proteins together and support substrate turnover by CYP147G1 utilising intracellular NAD(P)H. When the cells were grown and protein produced, the culture media took on a blue colouration indicating that indole generated in the growth media from tryptophan breakdown was being oxidised to indigo (Fig. S4†).^53^ By adding indole to the growth we were able to generate more indigo (Fig. S4†). When CYP147G1 was produced in *E. coli* in the absence of FdR1 and Fdx3 no indigo formation was observed, suggesting that the two electron transfer partners from *M. marinum* are required to support CYP147G1 activity. The CYP147G1 enzyme was produced in *E. coli* and purified by two ion exchange chromatography steps. After purification, CYP147G1 was tested for the characteristic absorbance of a P450, the Soret absorbance (Fig. S5†). The binding of CO to the reduced ferrous form of the heme centre results in the almost complete shift of the Soret peak to 450 nm (∼95%), indicating the viability of the CYP147G1 enzyme.^54^

Investigation of the substrate range of the purified CYP147G1 enzyme was undertaken by UV/Vis analysis of the spin state of the heme iron.^55^ Undecanoic acid resulted in the largest spin state shift (40% high-spin, compared to indole, <5%) and the dissociation constant was determined to be 25 ± 4 μM (Fig. S6†). These results indicated that undecanoic acid is complementary to the active site of CYP147G1 and this substrate was chosen for product formation studies.

Despite multiple attempts, purification of the electron transfer partners, Fdx3 and FdR1, in a soluble form have not been successful. The yield of both proteins after cell lysis was insufficient for further workup or detailed *in vitro* analysis of activity. As Fdx3 and FdR1 are required for the creation of a native-like electron transfer system *in vitro* it was necessary to use whole-cell oxidation systems to investigate product formation. Undecanoic acid was chosen as a substrate and after extraction and derivatisation with *N*,*O*-bis(trimethylsilyl)trifluoroacetamide/trimethylsilylchloride (BSTFA/TCMS), the turnovers were analysed *via* GC-MS (Fig. S7†). The CYP147G1 turnover of undecanoic acid showed one peak in addition to that of the substrate. Analysis of the mass spectrum fragmentation pattern for the hydroxyundecanoic acid products displayed an increase in the base peak at 117.1 *m*/*z* (Fig. S7†). This is consistent with cleavage next to the CHOSiMe_3_ group on the ω-1 carbon (forming a CH_3_CHOSiMe_3_^+^ fragment). NMR analysis confirmed the product of undecanoic acid turnover was the ω-1 hydroxylated acid (10-hydroxyundecanoic acid) from several diagnostic signals (Fig. S7†).

### The selectivity of CYP147G1 for Fdx3 and FdR1

In order to investigate the role of Fdx3 in transferring electrons to CYP147G1, and the importance of the tyrosine residue of its iron–sulfur cluster binging motif, we tested the turnover activity of the system by replacing Fdx3 with mutant forms of the protein and other ferredoxins. Mutant versions of the Fdx3 gene were generated in which the polar aromatic tyrosine was replaced with glycine or cysteine. These two mutations were chosen to mimic the iron–sulfur cluster binding motifs of a [3Fe–4S] and a [4Fe–4S] ferredoxin, respectively (Fig. S8†). The mutant ferredoxin enzymes were cloned into both plasmids of the whole-cell oxidation system, pETDuetFdx3/FdR1 and pRSFDuetFdx3/CYP147G1, and used to test the activity of the CYP147G1 enzyme coupled with the mutant and WT forms of the ferredoxin. Samples of the turnovers taken at 4 hours contained the 10-hydroxyundecanoic acid product (Fig. 4). The level of product formation when coupled with the WT Fdx partner was almost double that when coupled with either the Y12C or Y12G ferredoxins. Both mutant ferredoxins performed similarly (Fig. 4). Experiments with indole confirmed these results (Fig. S4†). These results show that the tyrosine amino acid in the ferredoxin binding sequence is important for the regulation of electron transfer to the CYP147G1 enzyme or for the stability of Fdx3.

Next we tested replacing Fdx3/FdR1 electron transfer system with alternatives. CYP147G1 was cloned into the pRSFDuet vector by itself and was tested with different combinations of pETDuetFdx(1–11)/FdR1 and the same vector containing alternative non-physiological electron transfer partners (see ESI, Fig S9† for more details). The results from the turnovers of indole and undecanoic acid were conspicuous, with only the Fdx3 containing system being able to support activity. This was apparent from both indigo formation in the colorimetric assays with indole and the generation of 10-hydroxyundecanoic acid in the GC-MS analysis with undecanoic acid (Fig. S4 and S9†). None of the other electron transfer systems displayed any evidence of P450 metabolite formation highlighting the selectivity of CYP147G1 for Fdx3.

Overall, these assays suggest that protein–protein interactions may be more important than the cluster environment for efficient electron transfer to CYP147G1. The mutation of tyrosine to cysteine and glycine would be expected to alter the properties of the iron–sulfur cluster while maintaining the protein–protein interactions and the assays with these mutants displayed significant activity (albeit reduced compared to the WT Fdx3). While replacing the ferredoxin with another, would alter the redox potential it would also change protein–protein interactions completely and disrupted enzyme activity.

### Assessing the activity of selected other ferredoxin electron transfer partners of *M. marinum*

In order to assess an entire CYP electron transfer chain the ferredoxin reductase, ferredoxin and CYP enzyme have to be isolated and the likely substrate for the CYP enzyme has to be identified. For a bacterium such as *M. marinum* with so many potential combinations of CYP enzymes and electron transfer partners this would be impractical. We therefore chose pairings of CYP enzymes and ferredoxins which showed promising levels of protein production in *E. coli* and were coupled (in the genome) to ferredoxin genes. We also chose ferredoxins with different residues at the variable position (**?**) of the iron–sulfur cluster motif. Based on the protein production data the combinations of genomically associated P450 and Fdx genes chosen were Fdx2(Thr)/CYP278A1, Fdx4(Asn)/CYP269A1, Fdx8(His)/CYP150A5 and Fdx9(Ser)/CYP105Q4 (Table 1). All the CYP enzymes were produced in sufficient yields enabling their purification.

CYP105Q4, CYP278A1 and CYP150A5 displayed the expected substrate-free low spin CYP UV/Vis spectra with a Soret maximum at 419 nm (Fig. S5†). CYP269A1 was more unusual in that it had a spectrum that resembled a high spin ferric heme spectrum with a Soret maximum at 390 nm (Fig. S5†). The addition of the imidazole antifungal agent miconazole shifted the heme Soret absorbance maximum of CYP269A1 to 423 nm, and bound with a *K*_d_ of 0.050 ± 0.007 μM (Fig. S10†). CYP105Q4, CYP278A1 and CYP150A5 showed the characteristic shift to 450 nm for the ferrous–CO bound forms. In the absence of substrate CYP269A1 had a large peak at 420 nm with a shoulder at 450 nm (Fig. S5†). When miconazole was added the peak at 450 nm increased, indicating the ferrous–CO bound form of the enzyme, was generated in greater quantity (Fig. S5†).

The addition of β-ionone was found to shift the spin state of CYP278A1 to the high spin form (50%, Fig. S5†). The binding affinity of CYP278A1 for β-ionone was also tight; *K*_d_, 5.1 ± 1.5 μM, Fig. S6.† 56 Screening a range of aromatic compounds for their ability to bind to CYP150A5 produced low Type I shifts in the Soret peak absorption (Fig. S6†).^57^ However, we found that addition of β-ionone induced a 60% shift to the high spin form and the binding affinity was also reasonably high; *K*_d_ of 41 ± 3 μM (Fig. S6†). None of the extensive range of substrates tested with CYP105Q4 altered the spin state from the low spin form. Having identified viable substrates for CYP150A5 and CYP278A1 and an inhibitor of CYP269A1 we attempted to produce and purify the associated ferredoxins, Fdx8, Fdx2 and Fdx4 respectively, as well as Fdx9. The codon optimised genes of each ferredoxin were obtained and a 6× His tag was added to the C-terminus by PCR (ESI†). Under aerobic conditions Fdx2 and Fdx9 (associated with CYP278A1 and CYP105Q4, respectively) did not produce significant levels of folded ferredoxin after cell lysis. However Fdx4 and Fdx8 were purified in significant quantities using an ion exchange step followed by affinity chromatography (∼1 mg of purified protein per litre of broth, Fig. S11†). The UV/Vis spectra of Fdx4 and Fdx8 showed characteristic absorbances of [3/4Fe–4S] cluster containing ferredoxins (Fig. 5).^18^,^30^

**Fig. 3:**
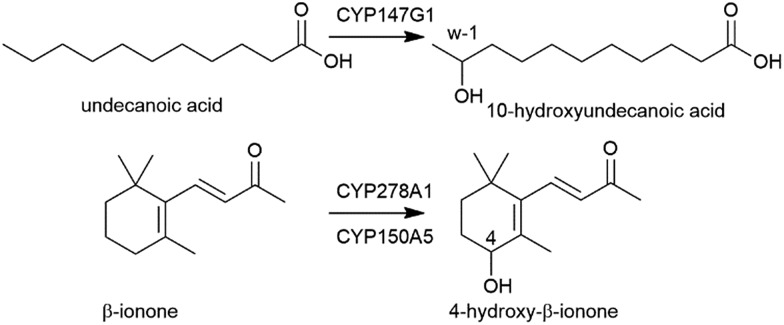
CYP147G1 oxidation of undecanoic acid to 10-hydroxyundecanoic acid by CYP147G1 and the oxidation of β-ionone to 4-hydroxy-β-ionone by CYP278A1 and CYP150A5.

**Fig. 4:**
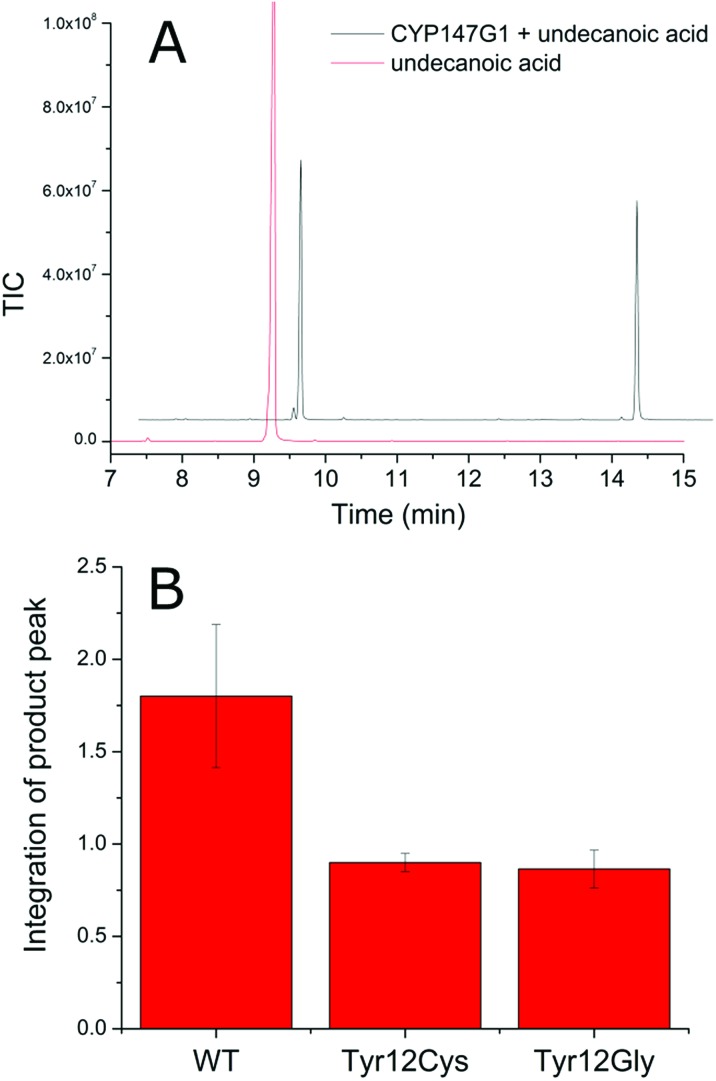
CYP147G1 product formation is reduced when supported by the mutant Fdx partners. (A) GC-MS chromatogram of the CYP147G1 turnover of undecanoic acid (black trace) after derivatisation with BSTFA/TMSCl. Derivatised undecanoic acid (RT 9.2 min, control red trace) and the 10-hydroxyundecanoic acid (RT 13.9 min) are shown. The chromatogram has been offset along the *x* and *y* axes for clarity. (B) Quantitation of the 10-hydroxyundecanoic acid product from variant Fdx3/CYP147G1 whole-cell turnovers of undecanoic acid. The axis shows the triplicate average of the area of integrated product peak divided by the area of the internal standard peak. Error bars show one standard deviation.

We next used whole-cell oxidation systems to study the monooxygenase activity of the FdR1/Fdx2/CYP278A1 and FdR1/Fdx8/CYP150A5 systems. GC and HPLC analysis of the turnovers of both systems showed a single product was formed from β-ionone oxidation. The product eluted at the same retention time for both systems. Co-elution experiments with turnovers of β-ionone using the CYP101B1 and P450Bm3 which generate the 3- or 4-hydroxy products, respectively, revealed that the sole product from both CYP278A1 and CYP150A5 systems was 4-hydroxy-β-ionone (Fig. S12†). This demonstrates FdR1 is able to support the activities of the Fdx2/CYP278A1 and Fdx8/CYP150A5 systems as well as Fdx3/CYP147G1 (Fig. 3, 4, S4, S9 and S12†).

### Further characterisation of the ferredoxins generated under anaerobic conditions

The purification of a range of the ferredoxins studied above was also undertaken under anaerobic conditions to further assess their redox activity and stability to oxygen. Fdx2 and Fdx3 yielded very low levels of folded protein making further study impossible. However, Fdx4 (Asn), Fdx5 (His) and Fdx9 (Ser) were isolated in good yields (ESI†).

**Fig. 5:**
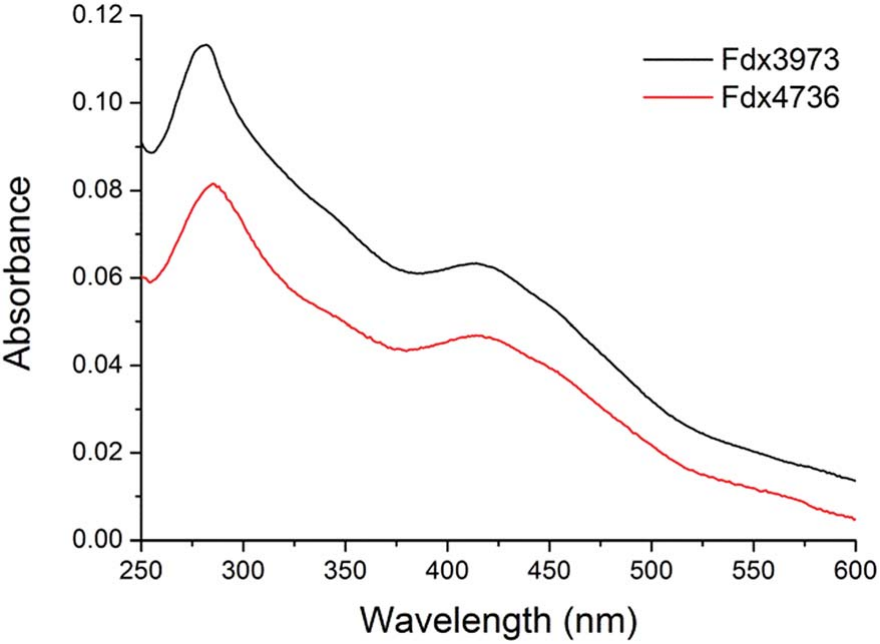
The UV/Vis absorbance spectra of aerobically purified Fdx4 (Fdx3973, black) and Fdx8 (Fdx4736, red) from *M. marinum*. Other spectra are included in the ESI.†

The ferredoxins were characterised by UV/visible absorbance and CD spectroscopy (Fig. S13†). The UV/visible absorbance spectra (Fig. S13†) were typical of proteins that contain either a [3Fe–4S] or [4Fe–4S] cluster. They consisted of a peak at approximately 430 nm with a larger extinction coefficient when oxidized than when reduced and showed no evidence of a peak at 450–460 nm, which is more commonly associated with [2Fe–2S] centres (Fig. 5 and S13†). As with all Fe–S cluster containing proteins a number of unresolved, low intensity transitions combine to produce a broad and generally featureless absorbance spectrum in the visible region. Whilst not diagnostic of cluster type, the bisignate nature of CD spectroscopy often affords greater resolution of these spectral components. This was the case with the spectra of Fdx4, Fdx5 and Fdx9, both as isolated and incubated with 1.5 mm ascorbate or excess EuCl_2_ as reductants or a stoichiometric equivalent of ferricyanide as an oxidant (Fig. S13†). The CD spectra of each of the ferredoxins in the oxidized state contained a negative feature at 420 nm (417 nm in the case of Fdx9) and a positive feature at 470 nm. In the absorbance spectra the apparent peak at 430 nm most likely arises from the scalar sum of the contributions from these two transitions. Reduction of the cluster results in a blue shift of the negative feature in the CD spectrum, to 409 nm for Fdx4 with a concomitant increase in intensity, and to 414 nm for Fdx5 with an apparent decrease in intensity due to overlap with a positive feature centred at 350 nm. Any shift of the corresponding feature in the spectrum of Fdx9 was less apparent since full reduction of the cluster could not be achieved with the reductants employed (see below). However, in all cases, CD spectroscopy afforded a more robust assignment of cluster oxidation state than the minimal changes observed in the absorbance spectra.

Fdx4, Fdx5 and Fdx9 were shown to bind a [3Fe–4S] cluster by a combination of non-denaturing ESI-MS and standard LC-MS (Fig. S14†). They also exhibited EPR spectra characteristic of [3Fe–4S] centres (*g* value ∼2.03, Fig. S15†) meaning that the cluster observed by mass spectrometry is unlikely to be the result of degradation during ionisation.^58^ A sample of Fdx4 purified by ion exchange and size exclusion methods had optical properties identical to those purified exploiting the poly-His tag, demonstrating that IMAC chromatography does not result in loss of a loosely bound Fe ion from the cluster. Furthermore, the CD and absorbance spectra of Fdx4 were unaffected by incubation with ferrous ion (Fig. S16†). We therefore conclude that each of the ferredoxins binds a [3Fe–4S] cluster following expression in *E. coli* and that *in vitro* incubation with Fe^2+^ does not lead to incorporation of a fourth metal ion and reconstitution of a [4Fe–4S] cluster. The stability of Fdx2 and Fdx4 to oxygen was also assessed by UV spectroscopy with no significant cluster degradation occurring on exposure to 120 μM O_2_ for 40 minutes (Fig. S17†).

Attempts to cycle the oxidation state of the clusters bound to anaerobically purified Fdx4, Fdx5 and Fdx9 were suggestive of large differences in their midpoint potentials on variation of the residue at position ‘**?**’ in the binding motif. The CD and electronic absorbance spectra of Fdx4 were altered by incubation with either a stoichiometric equivalent of K_3_Fe(CN)_6_ or excess EuCl_2_ as oxidant and reductant, respectively (Fig. S13†). This suggests a mixed oxidation state of the cluster in the protein isolated under anaerobic conditions with the cluster being readily oxidised by K_3_Fe(CN)_6_ and reduced by EuCl_2_. EPR spectra of the chemically poised samples (Fig S15†) were also consistent with oxidation and reduction to the EPR active and EPR silent forms of the cluster, respectively. Equilibration of Fdx4 with a 1.5 mM solution of sodium ascorbate resulted in a CD spectrum readily interpreted as a 50 : 50 sum of those of the oxidised and reduced clusters, suggesting a midpoint potential closely matched to the ascorbate solution potential (+60 mV *vs.* SHE). The optical spectra of Fdx5 were invariant following incubation with EuCl_2_ whilst incubation with a stoichiometric equivalent of K_3_Fe(CN)_6_ produced significant changes that were reversed upon subsequent incubation with excess EuCl_2_ (Fig. S13†). Therefore Fdx5 isolated under identical conditions to Fdx4 contains clusters predominantly in the reduced state. Incubation of reduced Fdx5 with 1.5 mM sodium ascorbate had no significant effect on the CD spectrum indicating that the cluster remained reduced at +60 mV, suggesting a midpoint potential greater than +150 mV.

In contrast, incubation with K_3_Fe(CN)_6_ had no effect on the optical spectra of Fdx9 whilst EuCl_2_ led to a reversible loss of CD intensity demonstrating that the protein was isolated with the cluster in the oxidised state (Fig. S13†). The EPR spectra of chemically poised Fdx9 samples indicated that 50% of the sample retained oxidised clusters following equilibration with excess EuCl_2_ (Fig. S15†) suggesting a midpoint potential similar to that of the Eu^3+^/Eu^2+^ couple (–360 mV *vs.* SHE).

**Fig. 6:**
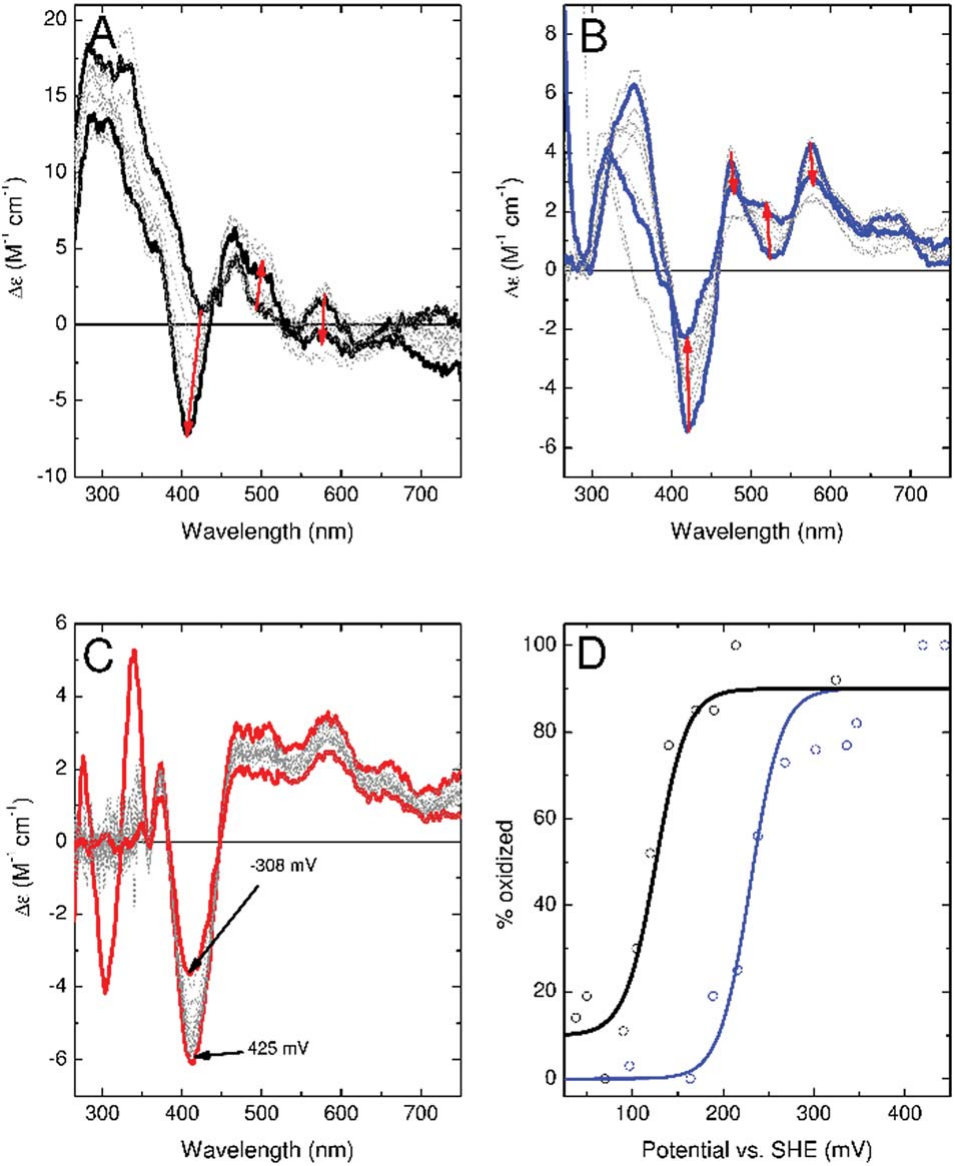
CD monitored potentiometric titrations of Fdx4 (Mmar3973, panel A), Fdx5 (Mmar4716, panel B) and Fdx9 (Mmar4763, panel C). Traces taken as representing fully oxidized and, where accessible, fully reduced cluster are shown in bold (for Fdx9 the bold traces correspond to the highest and lowest potentials at which spectra were recorded). Red arrows in panels A and B indicate the direction of spectral change upon reduction of the [3Fe–4S] cluster. Panel D shows the relative proportion of oxidized cluster for Fdx4 (black) and Fdx5 (blue) as a function of potential. Solid line shows the predicted behaviour for a single electron oxidation/reduction event with a midpoint potential of +120 mV *vs.* SHE (black line) or + 230 mV *vs.* SHE (blue line).

To confirm these observations, experiments were conducted to more accurately determine the redox potential of the ferredoxins. CD monitored titrations of Fdx4, Fdx5 and Fdx9 were performed in an optical cuvette containing a platinum electrode under a nitrogen atmosphere (Fig. 6). Samples containing a mediator cocktail were equilibrated with sodium dithionite or potassium ferricyanide and used to determine the potential of the solution (see ESI† for details). For Fdx4 and Fdx5 the spectral response was fitted to that predicted by the Nernst equation for a single electron oxidation/reduction event to yield midpoint potentials of +120 ± 13 mV for Fdx4 and +230 mV ± 16 mV for Fdx5, respectively (Fig. 6). For Fdx9 the CD response became obscured by absorbance features from added sodium dithionite before the potential could be lowered sufficiently to record the fully reduced spectrum. Whilst this precluded full analysis, titration to a potential of –308 mV *vs.* SHE allowed the estimate of an upper bound to the midpoint potential less than or equal to –340 mV for this cluster (Fig. 6). These values are in agreement with the observations reported above and highlight that varying the residue of the motif in each of these ferredoxins does have a significant effect on their redox properties.

## Discussion

The CYPome of *M. marinum* is larger than that of *M. tuberculosis* and the related *M. ulcerans*. There are also more electron transfer partner genes in *M. marinum* and these are closely associated with the genes of the CYPome. Given the relative number of CYP and electron transfer partner genes it is expected that each electron transfer ferredoxin could support multiple CYP enzymes. One interesting observation is that Fdx4 is associated with an unusual P450 with substrate free spectra indicating it exists in the high-spin form.

The majority of the ferredoxins are of the single cluster type and all have a non-cysteine residue in their iron–sulfur cluster binding motif, but not an alanine or glycine residue that commonly replaces the second cysteine of the motif in [3Fe–4S] ferredoxins. The identity of this residue varies among the ferredoxins of *M. marinum* and includes histidine, asparagine, threonine, tyrosine, serine and phenylalanine. The character of the non-cysteine residues modifies the environment of the iron–sulfur cluster and was shown to effect the properties and function of the ferredoxin. While many of these non-cysteine amino acid substitutions have the potential to act as ligands to metal ions and determine the cluster type ([3Fe–4S] or [4Fe–4S]) all of the ferredoxins were isolated as [3Fe–4S] forms under aerobic and anaerobic conditions. With the exception of the histidine containing version, these types of ferredoxins have not been characterised previously. The histidine containing ferredoxins from *Mycobacterium* and *Rhodopseudomonas* species were isolated aerobically as [3Fe–4S] ferredoxins.^18^,^30^–^32^ By way of contrast, histidine coordination to [4Fe–4S] iron–sulfur clusters has been observed in Ni–Fe and Fe-only hydrogenases and in [2Fe–2S] Rieske proteins.^61^–^63^

The most studied ferredoxin of this type is that from the thermophile *Pyrococcus furiosus* in which one of the iron ions of the cluster coordinates to an aspartate residue (**C**XX**D**XX**C**(X)_*n*_**C**P).^19^,^59^,^60^ Replacement of the more usual cysteine residue with aspartate alters the properties of this ferredoxin, most notably the reduction potential, compared to typical cysteinate-ligated [4Fe–4S] ferredoxins.^51^ This ferredoxin is isolated as a [3Fe–4S] ferredoxin under aerobic conditions but can be isolated as a [4Fe–4S] ferredoxin when oxygen is excluded. In common with other thermophilic ferredoxins, it also contains an additional disulphide bond which is thought to take part in the redox cycling of the enzyme. In contrast to the ferredoxins we have identified, the redox partner for the aspartate containing ferredoxin from *P. furiosus* is as yet unknown. This makes an analysis of the role of this residue in physiological electron transfer reactions difficult.

The identity of the iron–sulfur cluster, the ligands which coordinate to the metal ions and the surrounding environment can have a profound effect on the reduction potential of the ferredoxins. Cysteine-coordinated [2Fe–2S] containing ferredoxins (reduction potential –150 to –400 mV *versus* SHE) and the histidine Rieske equivalents (reduction potential +100 to +400 mV) contain two Fe(iii) ions in the oxidised form with one of these being reduced to Fe(ii) in the reduced form.^63^ It is usual for [4Fe–4S] clusters to shuttle between the [4Fe–4S]^2+/+^ state and have reduction potentials ranging from –280 to –715 mV, though high potential iron–sulfur clusters with potentials of +90 to +450 mV are known ([4Fe–4S]^3+/2+^).^63^ Previously characterised [3Fe–4S] ferredoxins have redox potentials ranging from –203 to –85 mV.^51^ The histidine containing [3Fe–4S] ferredoxin from *M. tuberculosis* (–31 mV) and the aspartate version from *P. furiosus* (–160 mV for 3Fe form and –375 mV for [4Fe–4S] form) both have significantly different reduction potentials compared to standard [3Fe–4S] and [4Fe–4S] ferredoxins. Our results show that the [3Fe–4S] ferredoxins from *M. marinum* have reduction potentials which vary between –340 to +230 mV. Those containing the neutral histidine and asparagine residues, are more positive compared to other proteins of this type. The serine containing ferredoxin (Fdx9) had a significantly lower redox potential, similar to those of the cysteine containing species. It seems probable that bacteria may use these different motifs to tune the redox potential in order to facilitate electron transfer to the different monooxygenases present.

The best characterised mycobacterial electron transfer ferredoxin of this type is encoded by the gene Rv0763c and is associated with CYP51 of *M. tuberculosis*.^30^ This ferredoxin has been shown to support the first electron transfer step of CYP51 from *M. tuberculosis* (but not the monooxygenase activity). The low activity observed was rationalised by the high reduction potential of the ferredoxin which is reported to be unfavourable for the reduction of the substrate bound CYP51. However, it is important to note that the recognition of the correct electron transfer partner by the P450 enzyme, through protein–protein interactions, may have a more important role in determining the selectivity. This will also contribute to the rate of these steps of the catalytic cycle. This was highlighted by the strict requirement of Fdx3 for the reconstitution of the activity of CYP147G1.

Given that the majority of the types of ferredoxins found in *M. marinum* have not been reported previously, we were surprised to discover that they are prevalent across a range of bacteria in particular in *Mycobacterium*, *Rhodococcus*, *Streptomyces* and other species of actinobacteria. They are also found in other bacteria. For example, the tyrosine containing ferredoxins are found in bacteria of the phylum *Chloroflexi*. Of particular interest is that bacterial operon predictions (; http://operondb.cbcb.umd.edu) show that the CYP147 genes in other bacteria including *Methanosarcina barkeri* (CYP147E1), *Myxococcus xanthus* (CYP147A1), *M*. *vanaabelinii* (CYP147G2) and *Streptomyces avermitilis* (CYP147B1), are all found next to a ferredoxin reductase encoding gene. All of these also have a gene present which encodes a similar ferredoxin to Fdx3 (Table S9†) which, in the case of *M. xanthus*, is a ferredoxin–ferredoxin reductase fusion protein. All of these ferredoxins contain a **C**XX**Y**XX**C**(X)_*n*_**CP** iron–sulfur cluster binding motif. Overall the data suggest that these three genes may form part of an operon with a similar function across many different species.

It is telling that in these bacteria the types of ferredoxins found in *M. marinum* are associated with CYP genes. It seems likely that they are involved in controlling the electron transfer pathways to enable the monooxygenase enzymes to synthesise complex natural products with a diverse array of biological function.

## Conclusions

Overall we have identified, isolated and characterised unusual ferredoxins from *M. marinum* containing [3Fe–4S] clusters containing proteins. We have used several of them in conjunction with a ferredoxin reductase to reconstitute the activity of their associated P450 enzyme. Similar ferredoxins are co-located with the CYPomes of *Mycobacteria*, as well as in many other types of bacteria. We found that the activity of CYP147G1 was reliant on using the physiological ferredoxin and that the identity of the altered residue of the motif was found to alter the redox potential. The diversity of these genes in *M. marinum* make it an excellent model organism for investigating electron transfer and its role in bacterial secondary metabolism. The observation of similar CYP systems in pathogenic bacteria such as *M. ulcerans* means these could be targets for drug design resulting in inhibition. The prevalence of these types of ferredoxins across the bacterial kingdom and their presence in the gene clusters of complex secondary metabolites highlights their importance in prokaryote secondary metabolism. Further study will lead to a better understanding of the role of the electron transfer partner proteins for efficient metabolism and natural product synthesis which in turn will allow the design of improved monooxygenase systems for applications in synthetic biology.

## Experimental

Phylogenetic analysis of the P450s and ferredoxins was carried out using standard methodologies as described in the ESI.† The cloning, protein purification steps, whole-cell turnovers, metabolite characterisation and all the aerobic and anaerobic protein analysis are described in full in the ESI.†

## Conflicts of interest

The authors confirm there are no conflicts of interest to declare.

## Supplementary Material

Supplementary information
