# Supplementary material for: Electron transfer ferredoxins with unusual cluster binding motifs support secondary metabolism in many bacteria
Source: Chem Sci. 2018 Aug 23;9(41):7948–57. doi: 10.1039/c8sc01286e (PMC6237146; doi:10.1039/c8sc01286e)
Supplement: Supplementary file 1 [file SC-009-C8SC01286E-s001.pdf]

Electronic Supplementary Material (ESI) for Chemical Science.
This journal is © The Royal Society of Chemistry 2018

Electron transfer ferredoxins with unusual cluster binding motifs support secondary metabolism in many bacteria

Stella A. Child,[1] Justin M. Bradley,[2] Tara L. Pukala,[1] Dimitri A. Svistunenko,[3] Nick E. Le Brun[2] and Stephen G. Bell[1,*]

Supporting information

Index

The CYPome of *Mycobacterium marinum* 2

Experimental

CYP assignments and alignments 4

Construction of whole-cell oxidation system for CYP147G1/Fdx3/FdR1 4

CYP enzyme production and purification 5

Ferredoxin purification 8

Spin state shift assays and binding constant determination 9

Whole-cell oxidation turnovers 9

Product analysis 11

Protein mass spectrometry 12

Characterisation of ferredoxins after anaerobic purification 12

CD monitored potentiometric titratons 14

Gene fragments and oligonucleotides used in this work 16

Tables S1-S9 20

Figures S1-S17 31

References 61

The CYPome of *Mycobacterium marinum*

There are forty seven CYP enzyme encoding genes in *M. marinum* and these belong to thirty six different P450 families and thirty nine subfamilies (Table S1 and Fig. S1). Those members of the same family but different subfamilies are CYP123A3 and CYP123B1 (43 %; sequence identity), CYP136A2 and CYP136B2 (46 %) and CYP138A3 and A4 and CYP138B1 (47 % and 45 %, respectively). There are multiple members of several subfamilies including CYP125A6 and A7 (75 %), CYP135B3, B4 (71 %) and B6 (66 % to B3 and 65 % to B4), CYP138A3 and A4 (62 %), CYP143A3 and A4 (58 %), CYP150A5 and A6 (55 %), CYP187A4 and A5 (57 %) and CYP189A6 and A7 (58 %). The proteins range from 389 to 491 amino acids in length and fourteen of the CYPs encoded by these genes are predicted to be positively charged at pH 7.0 with the remainder being negatively charged (Table S1).

The glutamate and arginine pair (EXXR) heme binding residues, which are highly conserved in the K-helix of the majority of CYP enzymes, was retained in all of those from *M. marinum* (Table S1).[1] The acid alcohol pair in the I helix was also conserved across most CYP members, the exceptions being CYP276A1 (GT) CYP135B3 (DN) and CYP51B1 (HT). Where it was conserved the acid residue was a mixture of aspartate and glutamate residues (18:27). The alcohol of this pair was predominantly threonine with only CYP144A4, CYP147G1 and CYP269A1 containing a serine residue (Table S1).[2] The phenylalanine residue which is often found seven residues before the conserved proximal cysteine (or eight if an additional glycine residue is inserted) was also mostly retained with only CYP183B1 (glutamate), CYP278A1 (leucine) and CYP147G1 (tryptophan) deviating from the norm (Table S1).[3]

The substrate profiles of several of the cytochrome P450 enzymes can be understood from those that have been previously studied in other mycobacterial species (see supporting information), e.g. CYP51B1 (sterol demethylase), CYP124A1 (phytanic acid ω-hydroxylation), CYP153A16 (alkane oxidation) and CYP142A3 and CYP125A7 (both cholesterol monooxygenases).[4-9] In addition to the CYP51B1, CYP124A1, CYP153A16, CYP142A3, CYP125A6 and CYP125A7 enzymes

mentioned above, several other CYP enzymes in *M. marinum* have homologues from *M. tuberculosis* which have been structurally characterised and tested with azole inhibitors (e.g. CYP130 and CYP144).[10, 11]

The likely substrates for some of the other CYP enzymes can be predicted based on their homology with other CYP enzymes e.g. CYP150 family members are reported to support aromatic hydrocarbon oxidation.[12] Finally the function of some can be inferred from their neighbouring genes as they are clustered with terpene and polyketide synthases and peptide synthetases. For example CYP226B1, CYP271A1, CYP183B1 and CYP274A1 are clustered together with enzymes involved in isoprenoid synthesis, CYP185A4 is found next to a gene with modules predicted to be responsible for the synthesis of an eight amino acid metabolite and a thioesterase domain, CYP139A3 is surrounded by genes involved in polyketide synthases and macrolide transport and CYP108B4, CYP187A4 and CYP187A5 are found together with genes involved in fatty acid and lipid metabolism. CYP genes such as CYP123A3 and CYP51B1 and CYP142A3 and CYP125A7, are found close together in a similar environment to those which have been described for other *Mycobacterium*.[13] For example the CYP125 and CYP142 genes are part of a conserved cholesterol metabolism gene cluster.[14]

There are only twenty CYP enzymes in *M. tuberculosis* and twenty four in *M. ulcerans* (plus ten pseudogenes) compared to forty seven in *M. marinum* (Table S3). This follows from the smaller gene complement of these bacteria due to reductive evolution (Table S1). Twelve are common to all three *Mycobacteria*, twenty one are unique to *M. marinum* with eleven being common to *M. marinum* and *M. ulcerans* strains and three shared by *M. marinum* and *M. tuberculosis* (Table S3). There is one unique cytochrome P450 encoding gene in *M. ulcerans*, CYP140A7, which is involved in the biosynthesis of Mycolactone A.[15] There are five unique cytochrome P450s in *M. tuberculosis*, of which one, CYP121A1, is essential for the bacterium's survival and is responsible for the formation of an intramolecular C-C bond in the cyclodipeptide cyclo(l-Tyr-l-Tyr).[16]

All of the CYP enzymes most closely associated with these ferredoxins have low pI values though other mycobacterial CYP enzymes have pI values higher than 7.0 (Table S1). The significance of the pI of the ferredoxin and CYP enzymes has not yet been fully established. However, based on the number of published studies on mycobacterial CYP enzymes those with low pI appear to be more readily produced in a soluble form in *E. coli*. This trend seems to extend across other bacterial families where CYP enzymes with a high pI (> 7.0) are not produced as readily.[4-7, 11, 16-19]

Of particular relevance to this work; CYP278A1 has significant sequence overlap with certain members of CYP109 family including CYP109D1 (*Sorangium cellulosum,* 42% sequence identity) and CYP109B1.[20] Norisoprenoids and fatty acids have been found to be efficiently oxidised by CYP109 family monooxygenases. The CYP150 enzyme from *Mycobacterium vanbalaanii* PYR1, which is in the same family as CYP150A5, has been reported to oxidise hydrophobic aromatic compounds.[12] CYP147 genes are also found in other bacteria including *Methanosarcina barkeri* (CYP147E1), *Myxococcus xanthus* (CYP147A1), *M. vanaabelinii* (CYP147G2, 68% sequence identity to CYP147G1) and *Streptomyces avermitilis* (CYP147B1).

Experimental

CYP assignments and alignments

CYP family and subfamily assignments were made by Dr. David Nelson according to the P450 nomenclature[21, 22] and are used as given in the National Centre for Biotechnology Information (NCBI) database. The genes and proteins from *M. marinum* M were compared to *M. ulcerans* Agy99, *M. tuberculosis* H37Rv and *M. leprae* TN.[23, 24]

Sequence alignments were performed using ClustalW. The evolutionary history was inferred by using the Maximum Likelihood method based on the Jones-Taylor-Thornton (JTT) matrix-based model.[25] The tree with the highest log likelihood is shown (Fig. S1). Initial tree(s) for the heuristic search were obtained automatically by applying Neighbor-Join and BioNJ algorithms to a matrix of pairwise distances estimated using a JTT model, and then selecting the topology with superior log likelihood value. The tree is drawn to scale, with branch lengths measured in the number of substitutions per site. The analysis of the CYPs involved the amino acid sequences of all forty seven enzymes from *M. marinum* and selected others for comparison. The analysis of the ferredoxins included all similar species from *M. marinum, M. ulcerans, M. tuberculosis* and other structurally characterised ferredoxins of this type. All positions containing gaps and missing data were eliminated. Evolutionary analyses were conducted in MEGA6.[26]

Construction of whole-cell oxidation system for CYP147G1/Fdx3/FdR1

General DNA manipulations and microbiological experiments were carried out by standard methods. The pETDuet and pRSFDuet vectors and the KOD Hot start polymerase used for the PCR steps were from Merck-Millipore. Enzymes for molecular biology were from New England Biolabs. The genomic DNA of *M. marinum* (ATCC 700278D-5) was obtained from Prof. Tim Stinear (University of Melbourne, Australia) and Prof. Lalita Ramakrishnan (University of Washington, USA, now at Cambridge University, UK). The genes encoding the P450 enzyme CYP147G1 (MMar_2930), CYP150A5 (MMar_4737), CYP269A1 (MMar_3969) CYP278A1 (MMar_2877) and the ferredoxin reductase FdR1 (MMar_2931) were amplified by PCR using

oligonucleotide primers (*vide infra*). The FdR1 and CYP genes were amplified by 30 cycles of strand separation at 95 ºC for 45 s followed by annealing at 55 ºC for 30 s and extension at 68 ºC for 80 s. The genes were cloned into the pET26 vector using the appropriate restriction enzymes. Codon optimised genes for Fdx2 (Mmar_2879), Fdx3 (Mmar_2932), Fdx4 (Mmar_3973), Fdx8 (Mmar_4736), Fdx9 (Mmar_4763) and the mutants of Fdx3 encoding the putative 3/4Fe-4S ferredoxin were obtained from IDT in the form of a g-block with NcoI and HindIII restriction sites incorporated at the 5′ and 3′ termini, respectively (for primer details, *vide infra*). The sequence encoding a 6xHis tag was inserted at the 3′ end of the gene using PCR (amplification for 30 cycles with strand separation at 95 ºC for 30 s followed by annealing at 55 ºC for 30 s and extension at 68 ºC for 20 s, for primers, *vide infra*).

The CYP genes (CYP147G1, CYP150A5, CYP269A1 and CYP278A1) were incorporated into the pET26 vector (Merck-Millipore) between NdeI and HindIII (XhoI for CYP150A5) restriction sites. The ferredoxin genes were cut using the NcoI and HindIII restriction enzymes introduced by PCR and cloned into the pETDuet and pRSFDuet vectors using the same restriction sites to yield pETDuetFdx and pRSFDuetFdx. The pETDuetFdx constructs were used to produce the encoded proteins using *E. coli*. To generate the whole-cell oxidation systems the FdR1 and CYP genes were cut using NdeI and KpnI (XhoI for CYP150A5) restriction enzymes. The FdR1 gene was cloned into the pETDuetFdx vectors to yield the plasmid pETDuetFdx/FdR1. The CYP genes were cloned into the appropriate pRSFDuetFdx (NdeI/KpnI) to yield the plasmid pRSFDuetFdx/CYP. The CYP enzymes above were also cloned into the pRSFDuet vector by themselves for analysis with different electron transfer partners (contained in the pETDuet vectors as described above).

Successful incorporation of the genes and mutants into each vector was confirmed by restriction enzyme digest followed by DNA sequencing (AGRF, Adelaide node) using the primers; T7 promoter, T7 terminator, pET Upstream, ACYDuetUP1, DuetUP2, DuetDOWN1 appropriate for each parent vector (Merck-Millipore).

CYP enzyme production and purification

The pET26 plasmid containing the appropriate CYP gene was transformed into BL21(DE3) competent *E. coli* and plated onto LB_{kan} plates and left overnight. A colony was picked and grown in 5 mL LB_{kan} at 37 °C and this starter culture was used to grow 2L of $2xYT_{kan}$ (4 x 500 mL) at 37 °C for 5 hours at 150 rpm. The incubator temperature was lowered to 25 °C, followed by the addition of IPTG (0.1 mM) and further incubation at this temperature for 16 hours at 120 rpm. The cells were harvested by centrifugation (5,000 *g*, 15 min) and resuspended in 200 mL of 50 mM Tris, pH 7.4, containing 1 mM DTT (henceforth Buffer T). The resuspended cells were kept on ice and lysed by sonication (25 cycles at 20:40 seconds on:off, 70 %, 19 mm probe, Sonics Vibra-Cell). The supernatant, containing the desired protein, was isolated from cell debris by centrifugation (40,000 *g*, 30 min). The protein was then loaded onto a DEAE Sepharose column, (XK50, 200 mm x 40 mm, GE Healthcare) and eluted using a linear salt gradient of 100 mM to 400 mM KCl in Buffer T. The fractions containing the desired protein (identified by red colour of the P450 enzymes) were combined and the volume reduced using a Vivacell 100 (Sartorius Stedim, 10 kD membrane) aided by centrifugation (1,500 *g*). The protein was desalted using a Sephadex G-25 medium grain column (250 mm x 40 mm) eluted with Buffer T. The desalted protein was then further purified by loading it onto a Source-Q ion-exchange column (XK26, 80 mm x 30 mm, GE Healthcare) and eluted with a gradient of 0 to 300 mM KCl in Buffer T. The fractions were selected for purity of protein by measurement of A_{418}/A_{280} ratio. Fractions with the highest A_{418}/A_{280} ratio were retained. The protein was concentrated via ultrafiltration and an equal volume of 80 % glycerol was added before filtration through a 0.22 μm syringe filter and storage at -20 °C.

To assess the viability of the P450 enzyme in the purified samples and determine the protein extinction coefficient, the enzyme was diluted to approximately 2 μM in Buffer T and the spectrum recorded between 300 and 700 nm on a UV/Vis spectrometer. A few flakes of sodium dithionite were added to reduce the iron and the spectrum recorded, then CO was gently bubbled through the cell and the spectrum was recorded. The extinction coefficient was determined using ε_{450} = 91

mM^{-1} cm^{-1} for the reduced CO-bound form. For CYP enzymes that did not fully shift to 450 nm, the concentration of the heme was determined by the pyridine hemochromagen assay as reported by Barr and Guo.[27]

Ferredoxin purification

The pETDuet vectors with the incorporated genes for Fdx2 (Thr; Mmar_2879), Fdx3 (Tyr; Mmar_2932), Fdx4 (Asn; Mmar_3973), Fdx5 (His; Mmar_4716), Fdx8 (His; Mmar_4736), Fdx9 (Ser; Mmar_4763) were transformed into competent *E. coli* BL21(DE3) and plated onto LB_{amp} plates and left overnight at 37 °C. A colony was picked and grown in 5 mL LB_{amp} at 37 °C overnight and this starter culture was then added across 2 L of $2xYT_{amp}$ (4 x 500 mL). After growing at 37°C for 5 hours at 150 rpm the incubator temperature was lowered to 25 °C, followed by the addition of benzyl alcohol (0.02 % v/v) and ethanol (2 % v/v) and then IPTG (0.1 mM) after a further 30 min. The cultures were incubated at this temperature for 16 hours with shaking at 120 rpm. The cells were harvested by centrifugation (5,000 *g*, 15 min) and resuspended in 200 mL of 50 mM Tris (pH 7.4) with DTT (1 mM) plus 30 mL glycerol, 3 mL β-mercaptoethanol and 1 mL TWEEN. Lysozyme (300 mg) was added and the resuspended cells were stirred on ice for 30 min, before sonication. The supernatant, containing the desired protein, was isolated from cell debris by centrifugation (40,000 *g*, 30 min). The protein was then loaded onto a DEAE Sepharose column, (XK50, 200 mm x 40 mm, GE Healthcare) and eluted using a linear salt gradient of 150 mM to 400 mM KCl in Buffer T. The fractions containing the desired protein (identified by brown colour of the Fdx enzyme) were combined and the volume reduced using a Vivacell 100 (Sartorius Stedim, 3 kD membrane) aided by centrifugation (1,500 *g*). The protein was loaded onto a His-trap column (GE Healthcare) equilibrated with Buffer T plus 300 mM NaCl and 20 mM imidazole. The column was washed with a further 5 column volumes before elution of the His-tagged protein with Buffer T containing 300 mM NaCl and 300 mM imidazole. The protein was concentrated via ultrafiltration and an equal volume of 80 % glycerol was added before filtration through a 0.22 μm syringe filter and storage at -20 °C.

Spin state shift assays and binding constant determination

Glycerol in stored protein samples was removed via buffer exchange into Buffer T using a PD-10 desalting column (GE Healthcare). The P450 was diluted to approximately 1 - 2 μM using the same buffer and the spectrum was recorded on a UV/Vis spectrophotometer. Aliquots (1 to 10 μL) of substrate stock solutions in DMSO or EtOH (50 mM - 100 mM) were added and the spectrum recorded until the shift from 420 nm to 390 nm reached a stable point. The ratio of high-spin P450 to low-spin P450 (390 nm peak to 420 nm peak) was estimated (to ± 5 %) by comparison to camphor-bound $P450_{cam}$ spectra.

For binding assays varying aliquots (1 to 3 μL) of substrate stock solutions (1 mM, 10 mM or 100 mM concentrations) were added to 2.5 mL of protein (0.5 - 1.5 μM). The sample was mixed and the absorbance difference spectrum was recorded between 300 nm and 600 nm on a UV/Vis spectrophotometer. Further substrate was added until no difference in peak-to-trough ratio at 420 nm and 390 nm was observed. The difference in absorbance versus substrate concentration was fitted to the hyperbolic function (Equation 1):

$$\Delta A = \frac{\Delta A_{max} \times [S]}{K_d + [S]}$$

where K_d is the binding constant, [S] is the substrate concentration, ΔA the peak-to-trough ratio, and ΔA_{max} the maximum peak-to-trough absorbance. Miconazole binding to CYP269A1 exhibited tight binding, with $K_d < 5$ μM and the data were fitted to the tight binding quadratic equation:[28]

$$\frac{\Delta A}{\Delta A_{\max}} = \frac{([E]+[S]+K_d) - \sqrt{\{([E]+[S]+K_d)^2 - 4[E][S]\}}}{2[E]}$$

where $\Delta A_{\max}$ is the maximum absorbance difference and [E] is the enzyme concentration.

Whole-cell oxidation turnovers

The appropriate DNA vectors pETDuetFdx/FdR1 and pRSFDuetFdx/CYP were mixed with competent BL21(DE3) *E. coli* cells, and grown on $LB_{amp/kan}$ plates for 16 hours at 37 °C. Colonies were inoculated into 2 mL $LB_{amp/kan}$ and grown at 37 °C for 4 hours at 200 rpm. This starter culture

was then added to 200 mL 2xYT$_{amp/kan}$ and grown at 120 rpm and 37 °C for a further 4 hours. The culture was cooled to room temperature, IPTG was added (to a final concentration of 1 mM) to induce protein expression and the growth was incubated overnight at 120 rpm. The cells were harvested via centrifugation (5,000 *g*, 10 min). The resulting cell pellet was resuspended in EMM (200 mL, 2 % DMSO), added to a baffled flask for increased aeration and the substrate was then added before shaking at 150 rpm at 30 °C.

The ability of CYP147G1 to oxidise indole (0.1 mM) to indigo was qualitatively determined colorimetrically via the formation of an intense blue colour when compared to the pET26CYP147G1 cultures as a control (Fig. S4). The cell pellet of this control was red in colour with no indication of any indigo formation in the cell pellet or the supernatant (Fig. S4).

After 16 hours the turnovers with other substrates were centrifuged (15 min, 5,000 *g*) and the supernatant isolated. Samples (1 mL) of the turnover were taken for initial testing and extracted with ethyl acetate (400 μL). For fatty acid substrates 3 M HCl (20 μL) was added to turnovers and the samples were extracted into ethyl acetate, dried over $MgSO_4$ before resuspension in anhydrous acetonitrile (200 μL). The acid samples were derivatised with TMCS/BSTFA before analysis by GC-MS. For other substrates the samples were extracted using ethyl acetate and the organic layer used directly for analysis.

For larger scale extractions 200 mL of the supernatant was acidified with 3 M HCl to pH 2, extracted three times with an equal volume of ethyl acetate. Extracts were washed with water and saturated brine solutions, combined and dried over $MgSO_4$ and the solvent was removed under reduced pressure.

To generate sufficient fatty acid product for characterisation the whole-cell oxidation system (200 mL) was used to convert 1 mM of the fatty acid substrates to product. The sample was extracted as described previously. Where GC-MS indicated a product:substrate ratio of ≥95 % reverse phase solid phase extraction (SPE) DSC-18 SPE tubes (Supelco) were used to isolate all fatty acid compounds, using the method described by Horak *et al* with minor modifications.[29] SPE

columns were activated with methanol (3 mL), washed with water (5 mL) and the extract was dissolved in methanol and water (200 µL) and loaded onto the column. The column was washed with 5 mL 10 % v/v methanol solution followed by 5 mL of 20 % v/v methanol:water. The acid products were eluted with 600 µL methanol and the elution was dried under a flow of nitrogen and dissolved in deuterated chloroform, 0.8 mL, before characterisation. Full product characterisation was performed by NMR. ^{1}H NMR and 2D COSY spectra were recorded at 500 MHz (Agilent DD2 500MHz NMR).

Product analysis

The activity of the mutant ferredoxins of Fdx3 with CYP147G1 was tested *in vivo* by performing turnovers in triplicate according to the procedure outlined above using 1 mM undecanoic acid as the substrate. Comparison of product formation by the wild type Fdx3 to the mutant Tyr→Cys and Tyr→Gly was analysed by GC-MS as before except that an internal standard (octanoic acid) was added to the samples (final concentration 0.5 mM) before extraction. The product yield was compared using the ratio of the area of the product peak versus that of the internal standard.

GC-MS analysis was used for enzyme turnover analysis and was performed on a Shimadzu GC-17A with a DB-5 MS fused silica column (30 m x 0.25 mm, 025 µm) and a QP5050A GC-MS detector. The injector was held at 250 °C and the interface at 280 °C. For fatty acid substrates, the initial oven temperature was 120 °C which was held for 3 min, before increasing to 220 °C at 7 °C per minute, where it was held for 7 min. The total ion count was monitored and the MS fragmentation pattern was recorded. For β-ionone, the initial oven temperature was 120 °C which was held for 3 min, before increasing to 220 °C at 10 °C per minute, where it was held for 7 min. The total ion count was monitored and the MS fragmentation pattern was recorded. Analytical liquid chromatography was performed using an Agilent 1260 Infinity pump equipped with an Agilent Eclipse Plus C18 column (250 mm x 4.6 mm, 5 µm), an autoinjector and UV detector. A gradient, 20 – 95 %, of acetonitrile (with trifluoroacetic acid, 0.1 %) in water (TFA, 0.1 %) was used.

Protein mass spectrometry

Protein mass measurements were carried out under denaturing conditions using an Agilent 6560 ion mobility quadrupole time-of-flight instrument with Dual AJS electrospray ionisation source, coupled to an Agilent 1290 Infinity II LC System. The protein was buffer exchanged into 250 mM ammonium acetate, concentrated to ~10 mM, then diluted 1:1 with acetonitrile. Protein sample (3 μL) was injected and electrosprayed using 50 % aqueous acetonitrile/0.01 % formic acid at a flow rate of 0.1 mL min^{-1}, without chromatographic separation. ESI-MS conditions were: positive-ion mode; capillary voltage, 3500 V; nozzle voltage 1000 V; fragmentor, 400 V; gas 8 L min^{-1}; gas temperature, 300 °C; sheath gas 11 L min^{-1}; and sheath gas temperature, 350 °C. Spectra were deconvoluted using BioConfirm software (Agilent).

Characterisation of ferredoxins after anaerobic purification

Protein was generated, using the same plasmid vectors as described above, from *E. coli* BL21(DE3) cells as follows. Culture was grown in LB to OD_{600} of 0.8 at 37 degrees 200 rpm shaking and cold shocked on ice for 18 minutes prior to induction with 50 μM IPTG. Post induction the cultures were supplemented with 20 μM ammonium ferric citrate and grown for a further 20 hours at 25 °C, 90 rpm shaking, to a final OD_{600} ~1.5.

After harvesting the pellets were re-suspended in anaerobic buffer (20 mM HEPES, 100 mM NaCl, 20 mM imidazole, pH 7.4), ruptured by sonication and centrifuged under nitrogen in gas tight tubes to remove debris. All subsequent steps were carried out anaerobically ($[O_2] < 10$ ppm). Supernatant was loaded onto a Ni^{2+} charged IMAC column equilibrated with the buffer above and eluted using a 50 mL gradient from 0 to 100 % buffer containing 500 mM imidazole. The sample was then exchanged into imidazole free buffer containing 1.5 M NaCl as cryoprotectant using a PD-10 column and stored in an anaerobic chamber at -5 ˚C prior to use.

In order to discount stripping of ferrous iron from the sample during IMAC purification a duplicate sample of Fdx4 was produced using the identical expression protocol as above. Pellets

were re-suspended in 20 mM HEPES, 50 mM NaCl, pH 7.4. Following sonication and centrifugation the sample was loaded on to a 5 ml FF Hi-Trap Q-sepharose column (GE Healthcare) and eluted using 20 mM HEPES, 500 mM NaCl, pH 7.4. The cluster containing fractions were pooled, concentrated and loaded on to a Sephacryl S-100 size exclusion column equilibrated with 20 mM HEPES, 500 mM, NaCl pH 7.4 and eluted using this buffer. Sample purity as judged from SDS-PAGE was as least equivalent to samples purified using an IMAC column (Fig. S9). Sample purified in this way gave an almost identical CD spectrum demonstrating that Fdx4 binds a [3Fe-4S] cluster regardless of the purification method used. The minor differences in the CD spectra obtained following the two preparation methods are consistent with slightly altered ratios of oxidised to reduced cluster in the samples obtained (Fig. S13).

Native- and LC-MS spectra of Fdx4, Fdx5 and Fdx9 were recorded on a Bruker micrOTOF-QIII mass spectrometer (Bruker Daltonics) equipped with an UltiMate 3000 HPLC system (Dionex). Samples for native MS were de-salted by dilution with an equal volume of 50 mM ammonium acetate pH 7.4 followed by exchange into the same buffer using a PD-10 column. Desalted samples were infused directly into the ionisation chamber at a flow rate of 300 μL min^{-1}. For LC-MS 50 μL samples were diluted with 450 μL of 2 % acetonitrile, 0.1 % formic acid solution. 1 μL of the diluted sample was then injected onto a ProSwift reversed phase RF-1S column at 25 ˚C and eluted using a gradient of 2 – 100 % acetonitrile, 0.1 % formic acid with a flow rate of 0.2 mL min^{-1} (15 min). Instrument parameters were: dry gas flow 8 L min^{-1}, nebuliser gas pressure 1.8 bar, dry gas temperature 240 ˚C and capillary voltage 4500 V (LC-MS) or dry gas flow 4 L min^{-1}, nebuliser gas pressure 0.8 bar, dry gas temperature 180 ˚C and capillary voltage 3000 V (native MS). Collision cell energy was 5.0 eV in all cases. Neutral mass spectra were calculated by maximum entropy deconvolution over the mass range 6 - 9 kDa using ESI Compass version 1.3 (Bruker Daltonics). The mass of the bound cluster was calculated from the difference in mass between the apo protein (deduced from LC-MS) and the cluster bound form (deduced from

native MS) and found to be 296 Da (corresponding to the predicted mass of a [3Fe-4S] centre) in all cases.

Redox activities of the [3Fe-4S] clusters of Fdx4, Fdx5 and Fdx9 were probed spectroscopically following anaerobic chemical poising. Electronic absorbance spectra were recorded on a Jasco V550 spectrophotometer and CD spectra on a Jasco J-810 spectropolarimeter. Sample concentration was estimated assuming an extinction coefficient ε_{410nm} = 9000 $M^{-1}cm^{-1}$. 250 μL samples of ferredoxin in 1 mm pathlength anaerobic cuvettes were equilibrated with either: 5 μL of an approximately 100 mM $EuCl_2$ solution in 20 mM HEPES, pH 7.4 containing 1.5 M NaCl (reduction), an appropriate volume of a 14 mM $K_3Fe(CN)_6$ solution in water to provide a 1:1 ratio with estimated cluster concentration (oxidation) or a 1.5 mM solution of sodium ascorbate (to set a defined solution potential of +60 mV).[30] Following characterisation by absorbance and CD spectroscopy, samples of as isolated and chemically poised proteins were loaded into EPR tubes and flash frozen by plunging into liquid N_2. EPR spectra were recorded at 10 K using a Bruker EMX (X-band) EPR spectrometer equipped with an Oxford Instruments liquid helium system and a spherical high-quality ER 4122 SP 9703 resonator (Bruker). Instrument parameters were as follows: microwave frequency ν_{MW} = 9.4657 GHz, modulation frequency ν_M = 100 kHz, time constant τ = 82 ms, microwave power = 3.19 mW, modulation amplitude A_M = 5 G, scan rate v = 22.6 Gs^{-1}. Estimated concentrations of [3Fe-4S] clusters prior to addition of chemical reagents were as follows; 180 μM (Fdx4 and Fdx9) and 330 μM (Fdx5). An equivalent sample of Fdx4 was anaerobically incubated with 180 μM $(NH_4)_2Fe(SO_4)_2$, subsequent characterisation (CD spectroscopy and redox activity) showed no evidence of incorporation of metal ion to yield a [4Fe-4S] centre.

CD monitored potentiometric titrations

Samples of each of each ferredoxin (4 mL) Fdx 4 (30 μM), Fdx 5 (45 μM) and Fdx 9 (40 μM) in 20 mm HEPES, 100 mm NaCl pH 7.4 were loaded into a bespoke optical cuvette capable of housing a

(Metrohm micro Pt titrode platinum electrode) under a nitrogen atmosphere. Samples contained the following mediators 3,6 –diaminodurene (DAD) (E_m = +276 mV vs. SHE), phenazine methosulphate (PMS) (E_m = +80 mV vs. SHE), phenazine ethosulphate (PES) (E_m = +55 mV vs. SHE), juglone (E_m = +30 mV vs. SHE), duraquinone (E_m = +5 mV vs. SHE), menadione (E_m = -70 mV vs. SHE), anthraquinone-2,6-disulphonic acid disodium salt (ADQS) (E_m = -185 mV vs. SHE), anthraquinone-2-sulphonic acid sodium salt monohydrate (AQS) (E_m = -225 mV vs. SHE) at approximately 1 μM concentration. CD spectra were acquired as described above following equilibration with 2 μL additions of stock solutions of sodium dithionite (reductive direction) or potassium ferricyanide (oxidative direction) injected through a rubber septum. The potential of the solution as reported by the Pt electrode was converted to a value vs. the standard hydrogen electrode (SHE) by calibration against the known potential (+295 mV) of a saturated solution of quinhydrone at pH 7.0. For Fdx 4 and Fdx 5 the spectral response was fitted to that predicted by the Nernst equation for a single electron oxidation/reduction event using Origin 8 (Origin Labs) to yield an estimate of the midpoint potential describing the process. For Fdx 9 the CD response became obscured by absorbance features from added sodium dithionite before the potential could be lowered sufficiently to record the fully reduced spectrum. Whilst this precluded analysis similar to that performed for Fdx 4 and 5, titration to a potential of -308 mV vs SHE allowed the estimate of an upper bound to the midpoint potential of this cluster.

Gene fragments and oligonucleotides used in this work.

Sequences of codon optimised ferredoxin genes; NcoI sites and HindIII sites were incorporated at the 5′ and 3′ ends respectively. The restriction sites NcoI and HindIII are underlined, start and stop codons highlighted in bold. Note that an additional codon GTG encoding a valine has been added to the N-terminus to allow for the incorporation of the NcoI site. A double stop codon was incorporated at the C-terminus. In the mutants the modified triplet codon and the amino acid are highlighted in red.

Fdx3 (MMar_2932)
tttaattcc**atg**gtgcgtctggtggttgatttaaacaaatgccaaggctacgctcagtgcgtaccactggcaccggaagttttcaagctggttggtg
aggaagctctggcttatgatccgaacccggacgactctcagcgtcagcgtgtactgcgcgcggtagcatcctgtccggttcaagcaattattctg
gaagtagacccgccggccgatcgcgacactaaa**taatag**aagcttaattaat

The amino acid sequence of Fdx3 with residues important in the ferredoxin binding motif highlighted in bold and underlined

MVRLVVDLNK**C**QG**Y**AQ**C**VPLAPEVFKLVGEEALAYDPNPDDSQRQRVLRAVAS**CP**VQAI
ILEVDPPADRDTK

Fdx3 Tyr-Gly mutant
tttaattcc**atg**gtgcgtctggtggttgatttaaacaaatgccaaggc**ggc**gctcagtgcgtaccactggcaccggaagttttcaagctggttggt
gaggaagctctggcttatgatccgaacccggacgactctcagcgtcagcgtgtactgcgcgcggtagcatcctgtccggttcaagcaattattct
ggaagtagacccgccggccgatcgcgacactaaa**taatag**aagcttaattaat

The amino acid sequence of Fdx3 with residues important in the ferredoxin binding motif highlighted in bold and underlined

MVRLVVDLNK**C**QG**G**AQ**C**VPLAPEVFKLVGEEALAYDPNPDDSQRQRVLRAVAS**CP**VQAI
ILEVDPPADRDTK

Fdx3 Tyr-Cys mutant
tttaattcc**atg**gtgcgtctggtggttgatttaaacaaatgccaaggc**tgc**gctcagtgcgtaccactggcaccggaagttttcaagctggttggtg
aggaagctctggcttatgatccgaacccggacgactctcagcgtcagcgtgtactgcgcgcggtagcatcctgtccggttcaagcaattattctg
gaagtagacccgccggccgatcgcgacactaaa**taatag**aagcttaattaat

The amino acid sequence of Fdx3 with residues important in the ferredoxin binding motif highlighted in bold and underlined

MVRLVVDLNK**C**QG**C**AQ**C**VPLAPEVFKLVGEEALAYDPNPDDSQRQRVLRAVAS**CP**VQAI
ILEVDPPADRDTK

Fdx2 (MMar_2879)
tttaattcc**atg**gtccgcgtggctgcggaccgcgagatctgtatggccaccggcatgtgtgtgatgaccgctgacgcattcttcgaccaggacgc
cgacggcattgttgtgctggctgcgcacgaagttccggcggacgaagagcgtagagttcgtaatgcagtgaaactgtgcccgtccggcgccct
ggaactgatgtccgat**taatag**aagcttaattaat

The amino acid sequence of Fdx2 with residues important in the ferredoxin binding motif highlighted in bold and underlined

MVRVAADREI**C**MA**T**GM**C**VMTADAFFDQDADGIVVLAAHEVPADEERRVRNAVKL**CP**SG
ALELMSD

Fdx4 (MMar_3973)
tttaattcc**atg**gttcgcgttatcgttgacgaaactctgtgcgaggcgaacggtttctgtgaaagtctggcaccagacatctttgctctgggcgatg
ctgatgtagttcagatcgctgacggcccggttcctgccgaccgccagatcgacgtgcgtgccgctgttgatcagtgcccgaaggccgccctgc
gtctgatcgag**taatag**aagcttaattaat

The amino acid sequence of Fdx4 with residues important in the ferredoxin binding motif highlighted in bold and underlined

MVRVIVDETL**C**EA**N**GF**C**ESLAPDIFALGDADVVQIADGPVPADRQIDVRAAVDQ**C**PKAAL
RLIE

Fdx5 (MMar_4716)
tttaattcc**atg**gtaaaagtttgggtggatccgcagcgttgtcaaggtcacaccctgtgtgctatgatcgcgccggacagcttccagctctctgaca
tcgacggttctagctcggcgatcagcgaaactgttccggctgatcagtgggacctggtgcgtgaagcggcgcatagctgtccggagcaggcg
atcgtcatcacc**taatag**aagcttaattaat

The amino acid sequence of Fdx5 with residues important in the ferredoxin binding motif highlighted in bold and underlined

MVKVWVDPQR**C**QG**H**TL**C**AMIAPDSFQLSDIDGSSSAISETVPADQWDLVREAAHS**CP**EQA
IVITDET

Fdx8 (MMar_4736)
tttaattcc**atg**gtcaaagtacgtgttgacgatcagcgttgccgcggccacggtatgtgcctgaccctgtgtccagaagtgttctctctgacggac
gatggttacgcagtggctatcactagcgacgtaccgatggaactggaagaggctgtgcgtgaagcgatccagtgctgcccggagcaggccat
ctccgaatctt**taatag**aagcttaattaat

The amino acid sequence of Fdx8 with residues important in the ferredoxin binding motif highlighted in bold and underlined

MVKVRVDDQR**C**RG**H**GM**C**LTLCPEVFSLTDDGYAVAITSDVPMELEEAVREAIQC**CP**EQAI
SES

Fdx9 (Mmar_4763)
tttaattcc**atg**gtgaaagtgatcgtagatgaaaatatctgcgcgtccagcggcaactgtgtgatgaatgcgccggaaattttcgaccagcgcga
cgaggacggcgtggtagtgctgcttaacgcaaatccgccagcggaactcgccgaaggtgcccgccgtgctgctgcttcttgcccggccctgg
caattaaaatcgaggag**taatag**aagcttaattaat

The amino acid sequence of Fdx9 with residues important in the ferredoxin binding motif highlighted in bold and underlined

MVKVIVDENI**C**AS**S**GN**C**VMNAPEIFDQRDEDGVVVLLNANPPAELAEGARRAAAS**CP**ALA
IKIEE

The primers used to insert a 6xHis Tag at the C-terminus of the ferredoxins. The NcoI and HindIII restriction sites and the sequence of the 6xHis are underlined and the start and stop codons are highlighted in bold.

Mmar_2879 5'	tttctatcc**atg**gtccgcgtggctgc
Mmar_2879 3'	attaattaagctt**ctatta**atgatggtggtgatgatgatcggacatcagttccagggc
Mmar_2932 5'	tttctatcc**atg**gtgcgtctggtggttg
Mmar_2932 3'	attaattaagctt**ctatta**atgatggtggtgatgatgtttagtgtcgcgatcggccgg
Mmar_3973 5'	tttctatcc**atg**gttcgcgttatcgttgac
Mmar_3973 3'	attaattaagctt**ctatta**atgatggtggtgatgatgctcgatcagacgcagggcg
Mmar_4716 5'	tttctatcc**atg**gtaaaagtttgggtggatc
Mmar_4716 3'	attaattaagctt**ctatta**atgatggtggtgatgatgggtttcatcggtgatgacgatc
Mmar_4736 5'	tttctatcc**atg**gtcaaagtacgtgttgac
Mmar_4736 3'	attaattaagctt**ctatta**atgatggtggtgatgatgagattcggagatggcctgctc
Mmar_4763 5'	tttctatcc**atg**gtgaaagtgatcgtagatg
Mmar_4763 3'	attaattaagctt**ctatta**atgatggtggtgatgatgctcctcgattttaattgccaggg

The primers used to clone the cytochrome P450 genes of CYP278A1 (*Mmar_2877*), CYP147G1 (*Mmar_2930*), CYP269A1 (*Mmar_3969*), CYP150A5 (*Mmar_4737*), CYP105Q4 and the ferredoxin reductase gene (*Mmar_2931*). The restriction sites are underlined and the start and stop codons are highlighted in bold. An additional KpnI site was added to all the genes to allow incorporation in the Duet vectors for the whole-cell oxidation system with the exception of CYP150A5 where the HindIII site at the 3′ end was replaced with XhoI.

CYP278A1 NdeI 5'	ttaattcat**atg**tcaacagagaccgtttcagg
CYP278A1 KpnI 3'	ttaattaagcttggtacc**ctatta**tgacaggtgcaggggtagc
CYP147G1 NdeI 5'	ttaattcat**atg**aatgccgaaaccgcttgggc
CYP147G1 KpnI 3'	ttaattaagcttggtacc**ctatta**ttcggtgatcgctgcgaaatc
FdR2931 NdeI 5'	ttaattcat**atg**aacccggggtcgttggtcg
FdR2931 KpnI 3'	ttaattaagcttggtacc**ctatta**gcctcggcggggccggaattc
CYP269A1 NdeI 5’	ttaattcat**atg**gcctatcctgaaaccaatac
CYP269A1 KpnI 3’	ttaattaagcttggtacc**ctatta**ccaacgcactggcagcgacag
CYP150A5 NdeI 5’	ttaattcat**atg**aatgattgtgccgagccgg
CYP150A5 XhoI 3’	ttaattctcgag**ctatta**tgcgggcgtgaaatccaaatg
CYP105Q4 NdeI 5’	ttaattcat**atg**tccgacacgctcgcaagcc
CYP105Q4 KpnI 3’	ttaattaagcttggtacc**ctatta**ccaggtcacgggtagttcatag

Table S1 Chromosome features of *M. marinum* M compared with three other *Mycobacteria*.

	M. marinum M	***M. ulcerans Agy99***	***M. tuberculosis H37Rv***	***M. leprae TN***
Size of Chromosome (bp)	6,636,827	5,631,606	4,411,532	3,268,203
Coding sequences	5424	4160	3974	1605
Pseudogenes	65	771	17	1115
**Number of P450 genes**	**47**	**24**	**20**	**1**
**Associated Fdx genes**	**11**	**6**	**2**	**0**
**Associated FdR genes**	**2**	**0**	**0**	**0**

Table S2 The CYPome of *M. marinum*. The gene and P450 name are assigned as per the databases at the National Center for Biotechnology Information. The sequences of conserved regions of the I-helix, K-helix and the heme binding motif as well as the predicted pI and length of the amino acid chain are also provided.

Gene name	Accession number	ID	I-helix	K-helix	Heme binding motif	pI	AA
Mmar_0122	ACC38592.1	CYP279A2	GTDTT	ETMR	IQTFGAGMHYCLG	4.9	411
Mmar_0272	ACC38739.1	CYP226B1	ATDTT	EGER	HATFGFGTHICSG	5.1	422
Mmar_0274	ACC38741.1	CYP271A1	GLDTV	ELMR	HLAFGSGIHRCLG	5.9	430
Mmar_0281	ACC38748.1	CYP183B1	GTETT	ETLR	YIPEGGGARKCIG	10.5	461
Mmar_0283	ACC38750.1	CYP274A1	ATETS	ETLR	FIPFGMGKHKCIG	9.6	450
Mmar_0346	ACC38813.1	CYP138A3	GHETT	EVQR	WIPFGGGTRRCVG	10.2	440
Mmar_0399	ACC38866.1	CYP191A3	GTETV	EMIR	SLAFGRGQHFCIG	5.2	401
Mmar_0852	ACC39310.1	CYP185A4	GEDTT	EAMR	YLPFGGGGRSCLG	8.8	473
Mmar_0928	ACC39385.1	CYP189A6	GNETT	ETLR	HLTFGKGVHYCLG	5.8	405
Mmar_0938	ACC39395.1	CYP135B4	GHDTT	ETLR	WLPFGGGNRRCLG	9.8	462
Mmar_1564	ACC40016.1	CYP276A1	AHGTT	ESLR	AVMFGAGIHYCLG	7.6	410
Mmar_1634	ACC40084.1	CYP136A2	AHDTS	ESIR	FTPFGGGAHKCLG	6.6	491
Mmar_2475	ACC40925.1	CYP139A3	GYETT	ETLR	FIPFSGGLHRCIG	9.8	432
Mmar_2631	ACC41077.1	CYP143A3	GLDTV	EIVR	HWGFGGGTHRCLG	5.1	389
Mmar_2654	ACC41098.1	CYP144A4	GGEST	ETLR	HISFGKGAHFCVG	4.8	403
Mmar_2666	ACC41109.1	CYP143A4	GLDTV	EIVR	HWGFGGGPHRCLG	6.1	390
Mmar_2768	ACC41210.1	CYP140A5	GFETT	EILR	HLAFSTGRHFCLG	8.4	437
Mmar_2783	ACC41225.1	CYP125A6	GNETT	EIVR	VGFGGTGAHYCIG	4.8	427
Mmar_2877	ACC41317.1	CYP278A1	GSETT	ETLR	HLSLGHGLHFCLG	4.8	426
Mmar_2930	ACC41369.1	CYP147G1	GHDST	EVQR	HFGWGSGIHTCMG	5.0	421
Mmar_2978	ACC41416.1	CYP135B6	GYDTS	ETLR	WLPFGGGARRCLG	10.3	472
Mmar_3135	ACC41569.1	CYP136B2	AHDTS	EALR	WVPFGGGAHKCIG	7.2	484
Mmar_3154	ACC41588.1	CYP153A16	GNDTT	EIIR	HISFGFGVHRCMG	6.0	462
Mmar_3361	ACC41787.1	CYP124A1	GNETT	EIVR	VGFGGGGAHFCLG	4.9	432
Mmar_3761	ACC42177.1	CYP268A2	GNDTT	ELVR	VGFGGGGVHFCLG	5.1	418
Mmar_3969	ACC42377.1	CYP269A1	GIDST	EVLR	HVSFGHGRFLCPG	4.8	402
Mmar_3976	ACC42384.1	CYP138A4	GHETT	EVQR	WIPFGGGTRRCIG	9.1	441
Mmar_3996	ACC42404.1	CYP187A4	GLETT	EGLR	HIAFAGGIHMCLG	4.8	407
Mmar_3999	ACC42407.1	CYP108B4	GHDTT	EMIR	VAFGYGVHFCMG	4.9	409
Mmar_4008	ACC42416.1	CYP187A5	GLETT	EGLR	HISFAAGEHTCLG	6.3	403
Mmar_4184	ACC42591.1	CYP130A4	GNDTV	ELLR	ILTFSHGAHHCLG	5.7	412
Mmar_4430	ACC42837.1	CYP138B1	GHETT	EVQR	WIPFGGGIHRCIG	8.4	455
Mmar_4483	ACC42889.1	CYP135B3	GHDNT	ETLR	WLPFGGGSRRCLG	10.1	456
Mmar_4694	ACC43098.1	CYP150A6	GQETT	ESLR	HMAFARGVHSCPG	5.0	423
Mmar_4717	ACC43121.1	CYP188A3	GFDTT	EFLR	HFSFGIGVHRCIG	4.9	453
Mmar_4733	ACC43137.1	CYP190A3	GAETV	ELLR	NTLGFGYGIHSCLG	4.8	399
Mmar_4737	ACC43141.1	CYP150A5	GQETT	EALR	HLSFGRGIHSCPG	4.9	422
Mmar_4753	ACC43157.1	CYP189A7	GNETT	ELLR	HLTFSVGTHYCLG	4.8	399
Mmar_4762	ACC43166.1	CYP105Q5	GHETT	ELLR	NVAFGYGRHQCVG	5.6	413
Mmar_4833	ACC43238.1	CYP123B1	GHETT	ELLR	VAFGRGIHFCLG	4.8	402
Mmar_4915	ACC43319.1	CYP126A3	GAETT	EMVR	LGFGQGVHYCLG	4.8	417
Mmar_4930	ACC43334.1	CYP123A3	GNETT	ETLR	LSFGSGAHFCLG	5.0	405
Mmar_4932	ACC43336.1	CYP51B1	GHHTS	ETLR	WIPFGAGRHRCVG	5.4	455
Mmar_5002	ACC43406.1	CYP142A3	GDETT	EMLR	LAFGFGTHFCMG	4.5	400
Mmar_5032	ACC43436.1	CYP125A7	GNETT	EIVR	VGFGGTGAHYCIG	4.6	416
Mmar_5175	ACC43581.1	CYP137A2	GHETT	ETLR	WVPFGGGAKRCLG	10.4	455
Mmar_5268	ACC43672.1	CYP164A3	GHETT	ETMR	HLGFGRGAHYCLG	4.7	441

Table S3 Analysis of the CYPomes of *M. marinum*, *M. ulcerans* and *M. tuberculosis*. The subfamily name of the *M. marinum* gene is given. If a pseudogene is present in *M. ulcerans* it has been highlighted in red and underlined. Ferredoxin (Fdx) and ferredoxin reductase (FdR) genes that are associated with CYP genes are highlighted in blue and italics. Neither of the ferredoxin reductase genes in *M. marinum* are present in *M. ulcerans* or *M. tuberculosis*. (A pseudogene is a dysfunctional relative of a gene which contains stop codon, frame shifts or insertions). Fdx11, which is not associated with a CYP gene, is conserved in all three bacterium.

***M. marinum* only**		***M. marinum* and *M. ulcerans***	**Conserved in all three**	***M. marinum* and *M. tuberculosis***
CYP123B1	CYP164A3	CYP105Q4 ***Fdx9***	CYP51B1 ***Fdx10***	CYP135B4
CYP125A6	CYP183B1	CYP108B4	CYP123A3	CYP137A2
CYP135B3	CYP189A6	CYP143A3	CYP124A1	CYP139A3
CYP135B6	CYP190A3***Fdx6/Fdx7***	CYP150A6	CYP125A7	
CYP136B2	CYP226B1	CYP185A4	CYP126A3	***M. tuberculosis* only**
CYP138A4	CYP268A2	CYP187A4	CYP130A4	CYP121A1
CYP138B1	CYP271A1	CYP187A5	CYP136A2	CYP128A1
CYP147G1	CYP274A1	CYP188A3 ***Fdx5***	CYP138A3	CYP132A1
Fdx3/FdR1	CYP276A1	CYP189A7	CYP140A5	CYP135A1
CYP150A5 ***Fdx8***	CYP278A1 ***Fdx2***	CYP191A3	CYP142A3	CYP141A1
CYP153A16n	CYP279A2	CYP269A1 ***Fdx4***	CYP143A4 ***Fdx1***	
[2Fe-2S]/FdR2			CYP144A4	
				***M. ulcerans* only**
			Fdx11	CYP140A7

Table S4 The genes encoding the [3/4Fe-4S] ferredoxins of *M. ulcerans* and *M. tuberculosis* which have equivalents in *M. marinum*. The sequences of iron sulphur cluster binding motif of the [3/4Fe-4S] ferredoxins as well as the predicted pI and length of the amino acid chain are provided.

Gene name	Accession number	ID	*Mycobacterium ulcerans* Iron Sulfur cluster binding motif	pI	AA
Mul_0316	ABL03046.1	Mul_1	**C**XX**H**XX**C**(X)$_n$**C**P	3.7	65
Mul_0334	ABL03060.1	Mul_2	**C**XX**S**XX**C**(X)$_n$**C**P	3.9	63
Mul_0472	ABL03175.1	Mul_3	**C**XX**H**XX**C**(X)$_n$**C**P	4.4	67
Mul_2873	ABL05156.1	Mul_4	**C**XX**T**XX**C**(X)$_n$**C**P	4.0	63
Mul_3090	ABL05334.1	Mul_5	**C**XX**H**XX**C**(X)$_n$**C**P	3.9	63
Mul_3830	ABL05923.1	Mul_6	**C**XX**N**XX**C**(X)$_n$**C**P	3.6	62
Mul_4066	ABL06117.1	Mul_7	**C**XX**N**XX**C**(X)$_n$**C**P	4.0	81

Gene name		ID	*Mycobacterium tuberculosis* Iron Sulfur cluster binding motif	pI	AA
Rv0763c	CCP43510.1	-	**C**XX**H**XX**C**(X)$_n$**C**P	4.6	67
Rv1786	CCP44552.1	-	**C**XX**H**XX**C**(X)$_n$**C**P	3.7	66
Rv3503c	CCP46325.1	-	**C**XX**N**XX**C**(X)$_n$**C**P	3.6	62

Table S5 The genes encoding the other potential electron transfer proteins of *M. marinum* which are not closely associated with CYP enzyme genes. The gene name as per the databases at the National Center for Biotechnology Information is provided. The predicted pI and length of the amino acid chain are provided as are the names of the equivalent genes in *M. ulcerans* and *M. tuberculosis*.[a] Mmar_2994 is 16 genes away from CYP135B6 [b] Mmar_5043 is 11 genes away from CYP125A7. No others are within 30 genes of members of the CYPome of *M. marinum.* While this does not rule out their ability to function as electron transfer partners for a P450 enzyme they could also be involved in other metabolic processes which require these types of proteins. We also cannot rule out that they may have evolved to support P450 electron transfer in these other bacteria.

Gene name	ID	*Mycobacterium Marinum* Iron Sulfur cluster binding motif	pI	AA	*M. ul*	*M. tb*
Mmar_2080 ACC40530.1	FdxA	7Fe Ferredoxin	3.9	114	Mul_3264 ABL05469.1	Rv2007c CCP44779.1
Mmar_2994[a] ACC41432.1	FdxA	7Fe Ferredoxin	4.1	113	-	-
Mmar_3421 ACC41846.1	FdxC	7Fe Ferredoxin	3.8	107	Mul_2700 ABL05017.1	-
Mmar_4274 ACC42683.1	FdxC	7Fe Ferredoxin	3.4	107	Mul_1025 ABL03633.1	Rv1177 CCP43933.1
Mmar_4794 ACC43198.1	2Fe-2S	2Fe-2S	3.9	93	Mul_0363 ABL03085.1	-
Mmar_1017 ACC39474.1	FdR3	FdR3	4.6	411	Mul_0769 ABL03414.1	Rv0688 CCP43431.1
Mmar_1526 ACC39977.1	FprA	FprA	5.1	455	Mul_2413 ABL04766.1	-
Mmar_3420 ACC41845.1	FprA	FprA	5.3	453	Mul_2699 ABL05016.1	Rv3106 CCP45916.1
Mmar_4646 ACC43049.1	FprB	FprB	7.1	560	Mul_0264 ABL02999.1	Rv0886 CCP43634.1
Mmar_5043[b] ACC43447.1	FdxB	FdxB	5.9	673	Mul_4117 ABL06161.1	Rv3554 CCP46376.1

Table S6 A list of other ferredoxin genes from strains of *Mycobacterium* which share the same ferredoxin cluster binding motif as those from *M. marinum*. Note the list is not exhaustive and more than one ferredoxin with the stated motif may be present in the strain listed. Ferredoxin genes with the **CXXTXXC**(X)$_n$**CP** motif were only found in a few strains closely related to *M. marinum* e.g. *M. ulcerans* and *M. liflandii*.
[a] may be a pseudogene. [b] a ferredoxin reductase gene is also located by the ferredoxin. Entries in red have a CYP enzyme clustered with the ferredoxin and those in blue have a CYP gene close by.

		**cluster binding motif**		
**CXXHXXC**(X)$_n$**CP**	**CXXNXXC**(X)$_n$**CP**	**CXXYXXC**(X)$_n$**CP**[b]	**CXXFXXC**(X)$_n$**CP**	**CXXSXXC**(X)$_n$**CP**
M. simiae<br>WP_044508369.1	*M. xenopi*<br>WP_039890166.1	*M. gastri*<br>WP_036416246.1	*M. kansasii*<br>WP_023367649.1	*M. asiaticum*<br>WP_036365249.1
M. colombiense<br>WP_007771429.1	*M. heckeshornense*<br>WP_048893299.1	*M. kansasii*<br>WP_023364435.1	*M. gastri*<br>WP_036414200.1	*M. lentiflavum*<br>CQD20859.1
M. kyorinense<br>WP_045384657.1	*M. intracellulare*<br>WP_014382133.1	*M. aromaticivorans*<br>WP_036346035.1	*M. asiaticum*<br>WP_036365153.1	*M. xenopi*<br>WP_039891214.1
M. kansasii<br>WP_023367619.1	*M. lentiflavum*<br>CQD18585.1	*M. rhodesiae*<br>WP_005140322.1	*M. parascrofulaceum*<br>WP_007168606.1	*M. simiae*<br>WP_044512409.1
M. lentiflavum<br>CQD20693.1	*M. asiaticum*<br>WP_036358226.1	*M. chlorophenolicum*<br>WP_048472356.1	*M. avium*<br>WP_003877260.1	*M. avium*<br>WP_023876861.1
M. xenopi<br>WP_003919555.1	*M. simiae*<br>WP_044507197.1	*M. vanbaalenii*<br>ABM11247.1	*M. lentiflavum*<br>CQD20744.1	*M. sinense*<br>WP_013830364.1
M. gastri<br>WP_036414692.1	*M. genavense*<br>WP_025738432.1[a]		*M. nebraskense*<br>WP_046181754.1	*M. heckeshornense*<br>WP_048890125.1
M. parascrofulaceum<br>WP_007166396.1	*M. kyorinense*<br>WP_045383051.1		*M. europaeum*<br>CQD03229.1	*M. genavense*<br>WP_025737053.1
M. sinense<br>WP_013830530.1	*M. avium*<br>WP_009975858.1		*M. heckeshornense*<br>WP_048890092.1	*M. heraklionense*<br>WP_047318276.1
M. nebraskense<br>WP_046186374.1	*M. parascrofulaceum*<br>WP_007168596.1		*M. xenopi*<br>WP_003922631.1	*M. kansasii*<br>WP_036393982.1
M. haemophilum<br>WP_047316334.1	*M. europaeum*<br>CQD03261.1		*M. vanbaalenii*<br>WP_041308028.1	
M. europaeum<br>CQD04192.1	*M. nebraskense*<br>WP_046181777.1		*M. gilvum*<br>WP_041799998.1	
M. phlei<br>WP_003889324.1	*M. thermoresistibile*<br>WP_003927083.1		*M. rufum*<br>WP_043411031.1	

Continued overleaf

cluster binding motif

**CXXHXXC**(X)$_n$**CP**	**CXXNXXC**(X)$_n$**CP**	**CXXYXXC**(X)$_n$**CP**	**CXXFXXC**(X)$_n$**CP**	**CXXSXXC**(X)$_n$**CP**
M. hassiacum WP_005630344.1	*M. iranicum* WP_024448284.1		*M. vaccae* WP_040539836.1	
M. tusciae WP_006241645.1	*M. phlei* WP_003889603.1		*M. chubuense* WP_048418201.1	
M. canettii WP_042914619.1	*M. heraklionense* WP_047319309.1		*M. chlorophenolicum* WP_048473402.1	
M. smegmatis WP_015309433.1	*M. rhodesiae* WP_014212260.1		*M. iranicum* WP_036465671.1	
M. thermoresistibile WP_003926283.1	*M. rufum* WP_043411043.1		*M. phlei* WP_003889597.1	
M. elephantis WP_046752094.1	*M. gilvum* WP_011893358.1		*M. elephantis* WP_046750099.1	
M. avium WP_019734709.1	*M. aurum* WP_048631967.1		*M. aurum* WP_048631973.1	
M. heckeshornense WP_048889694.1	*M. chubuense* WP_014816820.1		*M. tusciae* WP_006241377.1	
M. heraklionense WP_047320532.1	*M. tusciae* WP_006241383.1		*M. rhodesiae* WP_014212266.1	
M. goodie WP_049744806.1	*M. vaccae* WP_003928240.1		*M. thermoresistibile* WP_040547386.1	
M. rufum WP_043412685.1	*M. vulneris* WP_036448623.1		*M. smegmatis* WP_015308293.1	
M. setense WP_039382128.1	*M. smegmatis* WP_015308299.1			
M. mageritense WP_036434743.1				
M. aromaticivorans WP_036341668.1				

Table S7 A list of other ferredoxin genes from other bacteria which share the same ferredoxin cluster binding motif as those from *M. marinum*. Entries in red have a CYP enzyme clustered with the ferredoxin. Note the list is not exhaustive and more than one ferredoxin with the stated motif may be present in the strain listed. Ferredoxin genes with the **CXXTXXC**(X)$_n$**CP** motif were not found in other bacteria using BLAST searches of the NCBI database.

[a] clustered with a dioxygenase gene. [b] clustered close to a monooxygenase gene. [c] Part of a larger steroid degrading biosynthetic cluster which contains a P450 encoding gene. [d] a ferredoxin reductase gene is also located by the ferredoxin. [e] the ferredoxin gene is fused to the ferredoxin reductase gene.

		**cluster binding motif**		
**CXXHXXC**(X)$_n$**CP**	**CXXNXXC**(X)$_n$**CP**	**CXXYXXC**(X)$_n$**CP**[d]	**CXXFXXC**(X)$_n$**CP**	**CXXSXXC**(X)$_n$**CP**
Nocardia higoensis WP_040801623.1	*Nocardia pneumonia* WP_040773457.1[a,b,c]	*Nocardia jiangxiensis* WP_040830166.1	*Nocardia jiangxiensis* WP_040825470.1	*Saccharopolyspora spinosa* WP_010693449.1[e]
Nocardia farcinica WP_011209115.1	*Nocardia farcinica* WP_011207065.1[c]	*Streptomyces avermitilis* WP_037650459.1	*Microbacterium ketosireducens* KJL44014.1[a]	*Saccharomonospora cyanea* WP_005457293.1[e]
Gordonia amicalis WP_024497964.1	*Rhodococcus opacus* WP_005244549.1[c]	*Streptomyces natalensis* WP_030064645.1	*Rhodococcus rhodochrous* WP_033237125.1	*Streptomyces rapamycinicus* AGP60579.1[e]
Rhodococcus fascians WP_037176148.1	*Rhodococcus jostii* RHA1 ABG96480.1	*Streptomyces collinus* WP_020940041.1	*Saccharopolyspora spinosa* WP_010314919.1	*Streptomyces violaceusniger* WP_014058289.1[e]
Rhodococcus opacus WP_015888533.1	*Nocardioides luteus* WP_045549377.1	*Streptomyces antibioticus* WP_053212137.1		*Streptosporangium amethystogenes* WP_030915565.1
Frankia sp. EAN1pec WP_020462084.1	*Haliangium ochraceum* WP_012827286.1	*Streptomyces virginiae* WP_033214178.1		*Streptomyces durhamensis* WP_031160704.1[e]
Spongiibacter tropicus WP_051151222.1	*Frankia* sp. EAN1pec WP_020459245.1[a]	*Streptosporangium roseum* WP_012895195.1		*Saccharomonospora glauca* WP_005465046.1[e]
Novosphingobium malaysiense WP_039287162.1[a]	*Saccharomonospora halophile* WP_019811873.1	*Ktedonobacter racemifer* WP_052569463.1	***Continued overleaf***	*Streptacidiphilus albus* WP_034086903.1[e]

cluster binding motif				
**CXXHXXC**(X)$_n$**CP**	**CXXNXXC**(X)$_n$**CP**	**CXXYXXC**(X)$_n$**CP**	**CXXFXXC**(X)$_n$**CP**	**CXXSXXC**(X)$_n$**CP**
Streptomyces mirabilis WP_037736688.1	*Herbidospora cretacea* WP_030456498.1	*Actinopolymorpha alba* WP_020578491.1[e]		*Frankia* sp. CN3 WP_007510855.1[e]
Spirillospora albida WP_030164411.1	*Gordonia amarae* WP_005182707.1[b]	*Rhodococcus opacus* WP_012689438.1		
Saccharomonospora marina WP_009157031.1	*Actinomadura atramentaria* WP_019633183.1	*Methanosarcina barkeri* WP_048110266.1		
Actinomadura oligospora WP_026414661.1				
Tomitella biformata WP_024794878.1				
Amycolatopsis halophile WP_034277668.1				
Streptomyces griseus WP_037679114.1				
Kineosporia aurantiaca WP_052531168.1				
Methyloferula stellate WP_020176888.1				
Rhodopseudomonas palustris BisB5 WP_011502257.1				
Novosphingobium aromaticivorans ABP64540.1				

Table S8 Characterised gene clusters with a ferredoxin (located close to the CYP genes) which has a ferredoxin motif similar to those identified in *M. marinum* M. The gene name as per the databases at the National Center for Biotechnology Information or the gene cluster is provided. The sequence of the iron-sulfur cluster binding motif of the [3/4Fe-4S] ferredoxin is provided. The neighbouring CYP genes (all next to each other with one exception which is 2 genes away) are also shown. The data was obtained from the Minimum Information about a Biosynthetic Gene cluster (https://mibig.secondarymetabolites.org/index.html) and DoBiscuit (http://www.bio.nite.go.jp/pks/tutorial/view). Most species are from strains of *Streptomyces*.[a] a 2-Thiosugar-Containing Angucycline-Type Natural Product

**Natural product**	**Organism**	**Fdx Gene name**	**Fdx Motif**	**CYP gene name**
Griseorhodin A	*Streptomyces* sp. JP95	*grhO4*	CXX**S**XXC(X)$_n$CP	*grhO4*
Salinomycin2	*S. albus* subsp. *albus*	*salF*	CXX**T**XXC(X)$_n$CP	*salD*
Salinomycin3	S. *albus*	*slnE*	CXX**T**XXC(X)$_n$CP	*slnF*
Tetronomycin	*Streptomyces* sp. NRRL 11266	*tmn14a*	CXX**S**XXC(X)$_n$CP	*tmn14*
Rapamycin	*S. rapamycinicus*	*rapO*	CXX**S**XXC(X)$_n$CP	*rapN*
Phoslactomycin	*Streptomyces* sp. HK803	*plmT4*	CXX**T**XXC(X)$_n$CP	*plmT6*
Filipin	*S. avermitilis MA-4680*	*pteE*	CXX**S**XXC(X)$_n$CP	*pteD*
BE-7585A[a]	*Amycolatopsis orientalis* subsp. *vinearia*	*bexO*	CXX**S**XXC(X)$_n$CP	*bexK*
Chrysomycin	*S. albaduncus*	*chryY*	CXX**H**XXC(X)$_n$CP	*chryOIII*
Enterocin	*S. maritimus*	*EncQ*	CXX**S**XXC(X)$_n$CP	*EncR*
Lysolipin	*S. tendae*	*llpK*	CXX**S**XXC(X)$_n$CP	*llpOIV*
Xantholipin	*S. flavogriseus*	*xanK*	CXX**S**XXC(X)$_n$CP	*xanO2*
Cinnabaramide	*Streptomyces* sp. JS360	*ORF11*	CXX**S**XXC(X)$_n$CP	*cinL*
Leinamycin	*S. atroolivaceus* S-140	*lnmB*	CXX**S**XXC(X)$_n$CP	*lnmA*

Table S9 Conservation of the CYP147/Fdr1/Fdx3 operon across different bacterial families. Fusion is where the ferredoxin gene is fused to the ferredoxin reductase gene.
[a] This ferredoxin gene is associated with the ferredoxin reductase and CYP147 genes. [b] There are three other ferredoxin genes with the **C**XX**Y**XX**C**(X)$_n$**C**P motif in *Streptomyces avermitilis* MA-4680.

CYP147G1 operon			
**CYP147**	**FdR**	**Ferredoxin**	CXX?XX**C**(X)$_n$**C**P
***Frankia* sp. CN3**			
WP_007510563.1	WP_007510561.1	**fusion**	**Y**
***Methanosarcina barkeri* str. Fusaro**			
WP_011306930.1	WP_011306931.1	WP_011306932.1	**Y**
***Methylobacterium extorquens* CM4**			
WP_015952233.1	WP_015952234.1	WP_003597514.1	**Y**
***Methylobacterium radiotolerans* JCM 2831**			
WP_012318808.1	WP_012318807.1	WP_012318806.1[a]	**Y**
***Mycobacterium vanbaalenii* PYR-1**			
ABM11249.1	ABM11248.1	ABM11247.1	**Y**
***Myxococcus xanthus* DK 1622**			
ABF88234.1	ABF90123.1	**fusion**	**Y**
***Rhodococcus jostii* RHA1**			
WP_011595234.1	WP_009475197.1	WP_011595232.1	**Y**
***Rhodococcus opacus* B4**			
WP_012689440.1	WP_012689439.1	WP_012689438.1	**Y**
***Streptomyces avermitilis* MA-4680[b]**			
WP_010982022.1	WP_010982021.1	WP_010982020.1	**Y**
***Streptosporangium roseum* DSM 43021**			
ACZ91470.1	ACZ91469.1	ACZ91468.1	**Y**

Figure S1 A phylogenetic tree of the forty seven CYP enzymes from *M. marinum*. Also included for comparison are the mycobacterial CYP enzymes; CYP125A1, CYP51, CYP124, CYP121A1, CYP141A1, CYP128A1, CYP132A1 and CYP135A1 from *M. tuberculosis* and CYP125A7 and CYP140A7 from *M. ulcerans*. CYP101A1 (P450cam), CYP102A1 (P450Bm3), CYP108A1 (P450terp), CYP111A1 (P450lin) and CYP176A1 (P450cin) are also included.

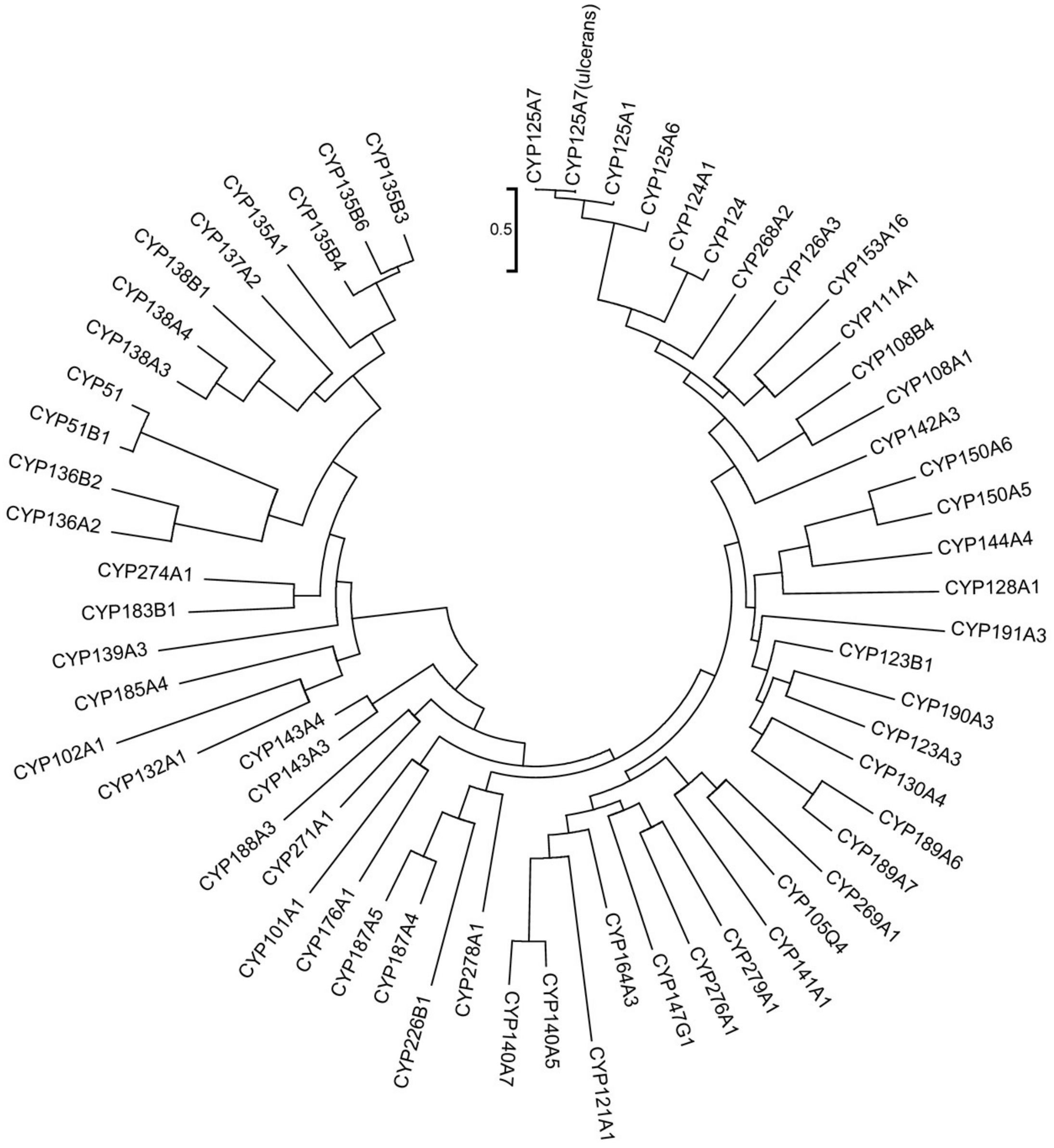

Figure S2 Sequence alignment of the eleven ferredoxins of *M. marinum* (Fdx1 – Fdx11). A structurally characterised histidine containing ferredoxin from *R. palustris* HaA2 (PDB: 4ID8) is shown for reference. The three conserved cysteine residues are highlighted in red while the location of the variable residue (CXX?XXC(X)nCP) is highlighted in the blue box. For comparison the [3Fe-4S] ferredoxins from *Streptomyces coelicolor* and [4Fe-4S] ferredoxins from *Clostridium thermoaceticum* and *Thermococcus litoralis* are also included (see Fig. 2 for further details on each species).

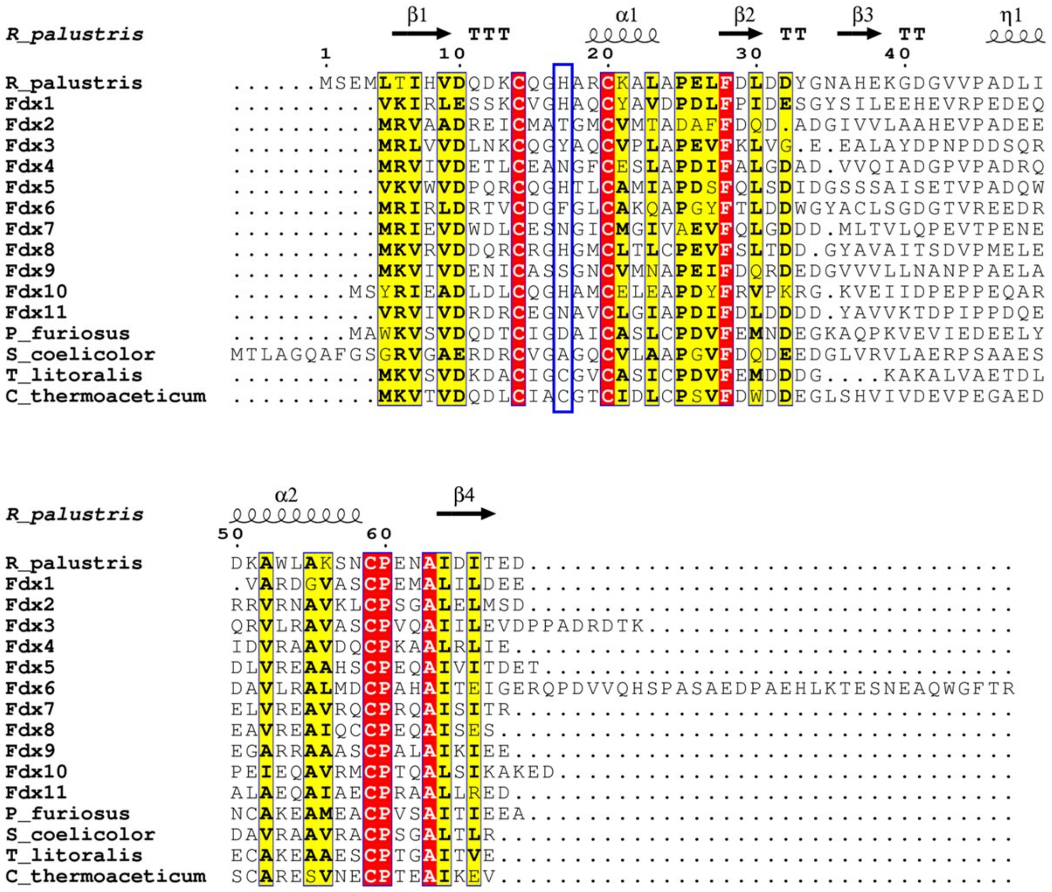

Figure S3 The genes surrounding the ferredoxin genes of *Mycobacterium marinum* M and comparison with other *Mycobacteria* and bacteria which contain an equivalent ferredoxin. The Microbial Genomic Context Viewer (MGcV)[31] was used to compare genome regions encoding homologous ferredoxins. Genes are labelled by their locus tag and coloured by COG (Clusters of Orthologous Groups). The ferredoxin gene is the in the centre of each figure (yellow triangle) and the genes on either side are shown.

(a) MMar_2666 (CYP143A4), associated with Fdx1 (Mmar_2667), comparison to *M. ulcerans* Agy99 (Mul_3091), *M. liflandii* 128FXT (MulP_04211), *M. tuberculosis* H37Rv (RVBD_1785c), *Frankia alni* CAN 14a (FRAAL3663) and *Rhodococcus jostii* RHA1 (RHA1_ro00423). The surrounding genes in the *Mycobacterium* species are similar while those in the *Frankia* and *Rhodococcus* species show greater variation (though the CYP143A4 gene is conserved). MalQ (Mmar_2663) is a 4-alpha-glucanotransferase, Mmar_2664 and Mmar_2665 gene products are predicted to be ESX conserved components eccB5 and eccC5. The genes from Mmar_2669 and Mmar_2677 encode PE, PPE, ESAT and ESX proteins.

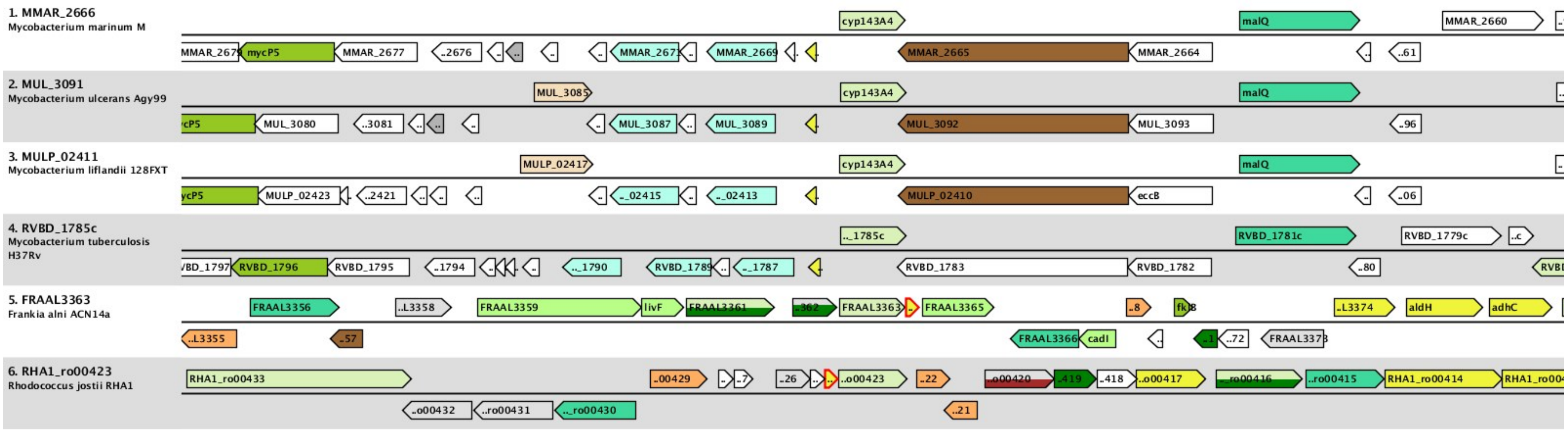

(b) Mmar_2877 (CYP278A1), associated with Fdx2 (Mmar_2879), comparison to *M. liflandii* 128FXT (MulP_02643), *M. rhodesiae* NB83 (MycrhN_4190), *M. vanbaalenii* PYR-1 (Mvan_3974). While the CYP278A1 and ferredoxin gene are conserved across the *Mycobacterium* as are some of the surrounding genes though there are differences in the surrounding region.

Mmar_2876 encodes an unknown regulatory protein and Mmar_2875 is predicted to be an aldehyde dehydrogenase. The cluster of genes from Mmar_2878 to Mmar_2897 encode integral membrance proteins, Mce3 proteins believed to be involved in cell division and associated proteins.

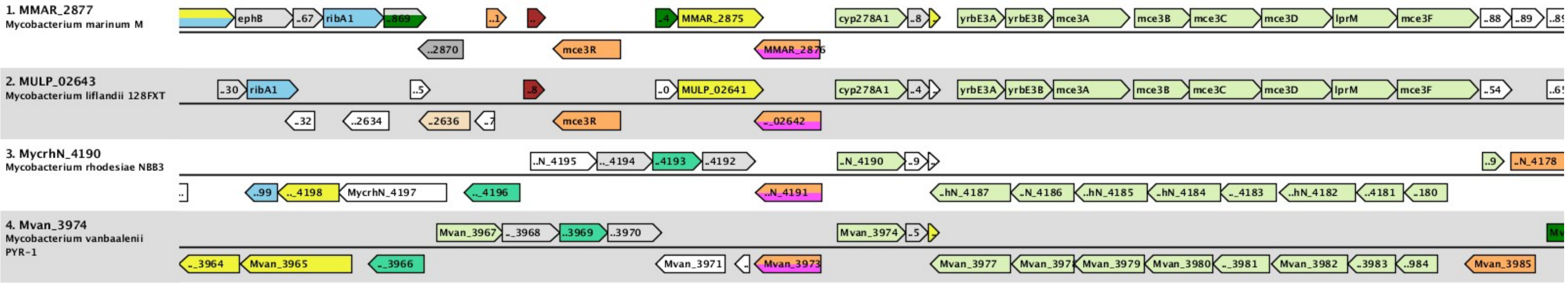

(c) Mmar_3154 (CYP153A16), associated with the [2Fe-2S] ferredoxin (Mmar_3155) and the ferredoxin reductase (FdR2, Mmar_3153), comparison to *Rhodococcus erythropolis* PR4 (pREL1_0283), *M. gilvum* PYR-GCK (Mflv_4592), *M. rhodesiae* NB83 (MycrhN_5185). The ferredoxin reductase (FdR2) is conserved in *M. gilvum* PYR-GCK but not *M. rhodesiae* NB83 or *R. erythropolis* PR4.
Mmar_3150 encodes a NAD dependent zinc-containing alcohol dehydrogenase, Mmar_3151, a medium chain fatty-acid-CoA ligase, Mmar_3152, a protein of unknown function. MMar_3156 encodes a transcriptional regulatory protein.

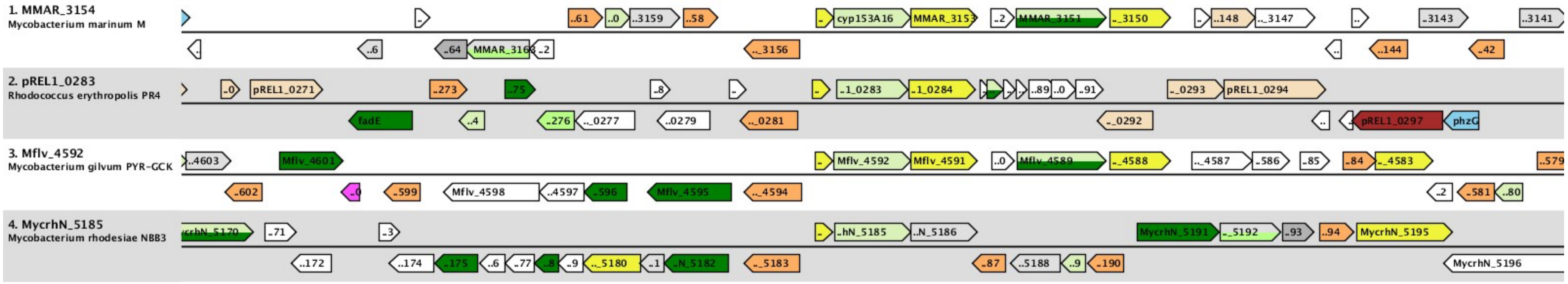

(d) Mmar_3969 (CYP269A1), associated with the Fdx4 (Mmar_3973), comparison to *M. liflandii* 128FXT (MulP_04142). The ferredoxin is not conserved in *M. liflandii* 128FXT.
Mmar_3962 encodes a fatty acid synthase, Mmar_3964 a subunit of a ring hydroxylating dioxygenase, Mmar_3965 a NAD-dependent aldehyde dehydrogenase, Mmar_3966 an acyl dehydratase.
Mmar_3971 encodes a probable oxidoreductase and Mmar_3972 an enzyme involved in non-ribosomal peptide synthetase.
Mmar_3974 encodes an acyl-CoA transferase.

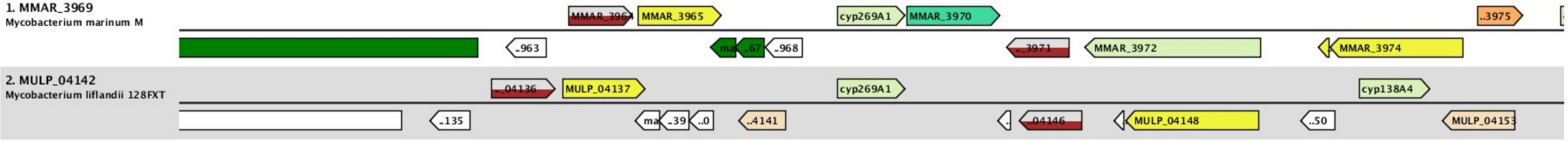

(e) Mmar_4717 (CYP188A3), associated with Fdx5 (Mmar_4716), comparison to *M. ulcerans* Agy99 (Mul_0317), *M. kansasii* ATCC 12478 (MKAN_09840), *M. vanbaalenii* PYR-1 (Mvan_4160) and *M. smegmatis* str. Mc2 155 (155 (MSMEI_4679). Several of the surrounding genes are highly conserved across the *Mycobacterium*. Of particular note is the close proximity of the CYP150A6 gene in *M. ulcerans* Agy99 (the equivalent gene in *M. marinum* M is Mmar_4694).

Mmar_4705 to Mmar_4710 encode proteins in involved in MCE (mammalian cell entry), Mmar_4711 and Mmar_4712 conserved hypothetical proteins of unknown function, Mmar_4713 a proposed dehydratase, Mmar_4714 a NAD-dependent aldehyde dehydrogenase and Mmar_4715 a NAD-dependent aldehyde dehydrogenase.

Mmar_4718 and Mmar_4719 encode acyl-CoA dehydrogenases and Mmar_4722 a membrane-associated phospholipase plcB_6 possibly involved in sphingomyelin and phosphatidylcholine hydrolysis.

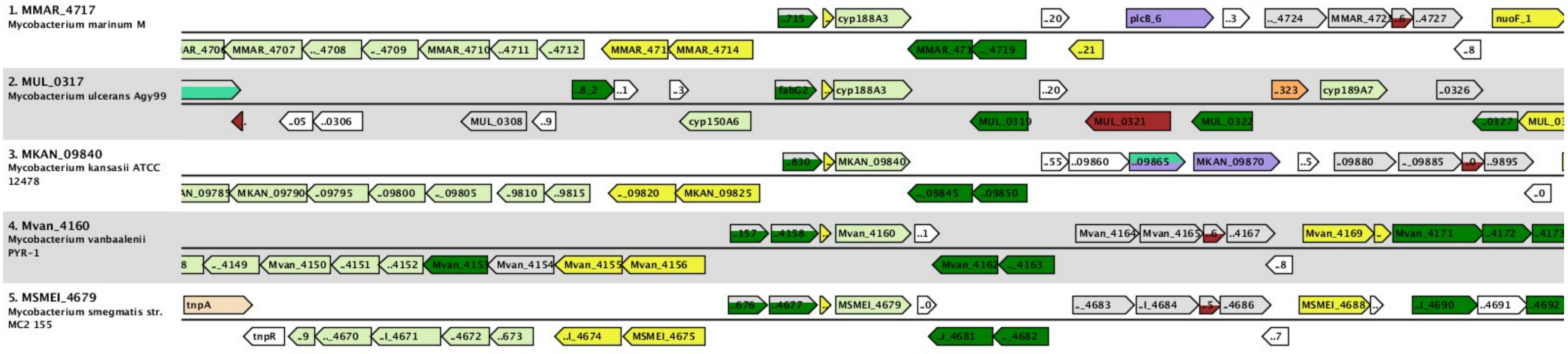

(f) Mmar_4730 (Fdx6), associated with CYP190A3 (Mmar_4733), Fdx7 (Mmar_4734), Fdx8 (Mmar_4736) and CYP150A5 (MMar_4737), comparison to *M. vanbaalenii* PYR-1 (Mvan_4170), *M. smegmatis* str. Mc2 155 (MSMEI_4689), *M. kansasii* ATCC 12478 (MKAN_09910) and *M. gilvum* PYR-GCK (Mflv_2484). Many of the surrounding genes are highly conserved across the *Mycobacterium*. Of particular note is the close proximity of the genes equivalent to CYP188A3 and it associated ferredoxin in *M. vanbaalenii* PYR-1 and *M. smegmatis* str. Mc2 155 (see Fig S2(e)). Mmar_4724 and Mmar_4725encode hypothetical metal-dependent hydrolase, Mmar_4726, similar to a Rieske ferredoxin subunit of certain proteins, a Mmar_4727, a hydrolase, Mmar_4729 a protein with similarity to NADH dehydrogenase I (chain F).
Mmar_4732 and Mmar_4732 encode a 3-ketoacyl-CoA thiolase and a thioesterase, respectively.
Mmar_4735 encodes a PE-PGRS family protein.
Mmar_4739 encodes a transcriptional regulatory protein, Mmar_4740 and Mmar_4741, an acetyl-CoA acetyltransferase FadA6_2 and an acyl-CoA dehydrogenase FadE12_1, respectively and Mmar_4742 a metal-dependent amidohydrolase.

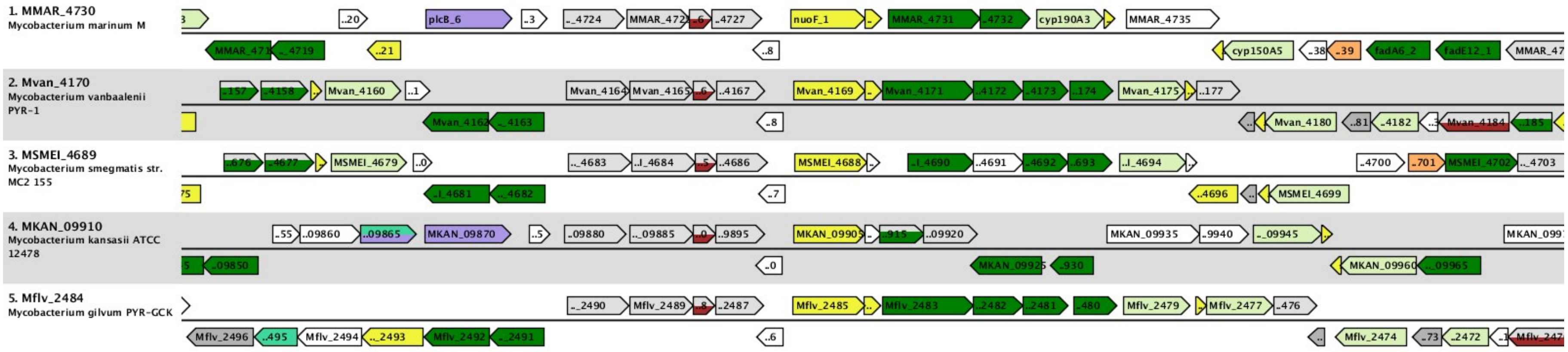

(g) Mmar_4762 (CYP105Q4), associated with Fdx9 (Mmar_47633), comparison to *M. ulcerans* Agy99 (Mul_0333), *M. vanbaalenii* PYR-1 (Mvan_4234), *M. gilvum* PYR-GCK (Mflv_2418), *M. smegmatis* str. Mc2 155 (MSMEI_4731) and *Amycolatopsis mediterranei* RB (B737_7119). Many of the surrounding genes are highly conserved across the *Mycobacterium*. A ferredoxin gene is also found in close proximity to the P450 gene in *A. mediterranei* RB.

Mmar_4756 to Mmar_4760 encodes an oxidoreductase, an acyl-CoA transferase, an enoyl-CoA hydratase, EchA4_1, an aminopeptidase and a dipeptidase, respectively. The function of Mmar_4761 is unknown.

Mmar_4764 may encode a transcription regulator, Mmar_4765 encodes a short chain dehydrogenase, Mmar_4766 a hypothetical protein, Mmar_4767 a membrane transport protein, Mmar_4768 a cysteine synthase a CysK2, Mmar_4769 a lipoprotein LpqS, Mmar_ Mmar_4770 an oxidase.

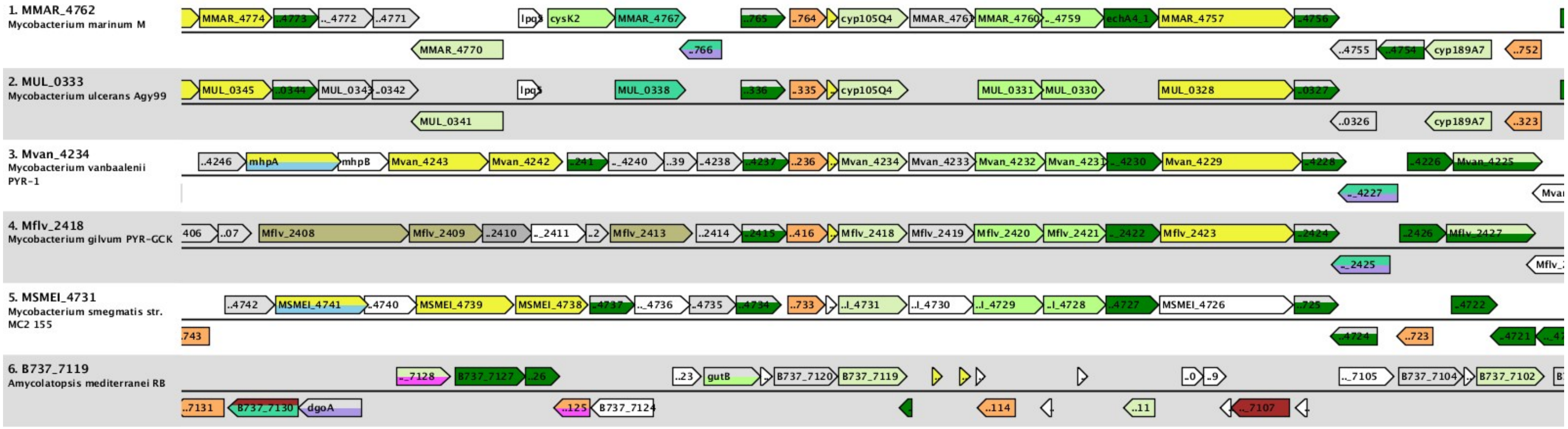

(h) Mmar_4932 (CYP51B1), associated with Fdx10 (Mmar_4933) and CYP123A1 (Mmar_4930), comparison to *M. ulcerans* Agy99 (Mul_0473), *M. vanbaalenii* PYR-1 (Mvan_5161), *M. gilvum* PYR-GCK (Mflv_1596), *M. tuberculosis* H37Rv (RVBD_0764c), *M. smegmatis* str. Mc2 155 (MSMEI_5704), *Rhodococcus jostii* RHA1 (RHA1_ro04671) and *Nocardia farcinica* IFM 10152. Many of the surrounding genes are highly conserved across the *Mycobacterium*. Several of the genes are also conserved in the *R. jostii* RHA1 species but the genes in the vicinity of the CYP51 gene in *N. farcinica* IFM 10152 are significantly different.

Mmar_4925 though to Mmar_4928 encode a phosphoribosylamine-glycine ligase, PurD, a 4-carboxymuconolactone decarboxylase, two dehydrogenase/reductases and a NAD-dependent aldehyde dehydrogenase, AldA. Mmar_4929 encodes a protein of unknown function.

Mmar_4931 encodes a short-chain alcohol dehydrogenase.

Mmar_4934 to Mmar_4937 encodes a conserved/hypothetical protein, a zinc-containing alcohol dehydrogenase NAD-dependent AdhB, a possible NADH:flavin oxidoreductase and an unknown conserved hypothetical protein containing a nuclear transport factor 2 (NTF2) domain.

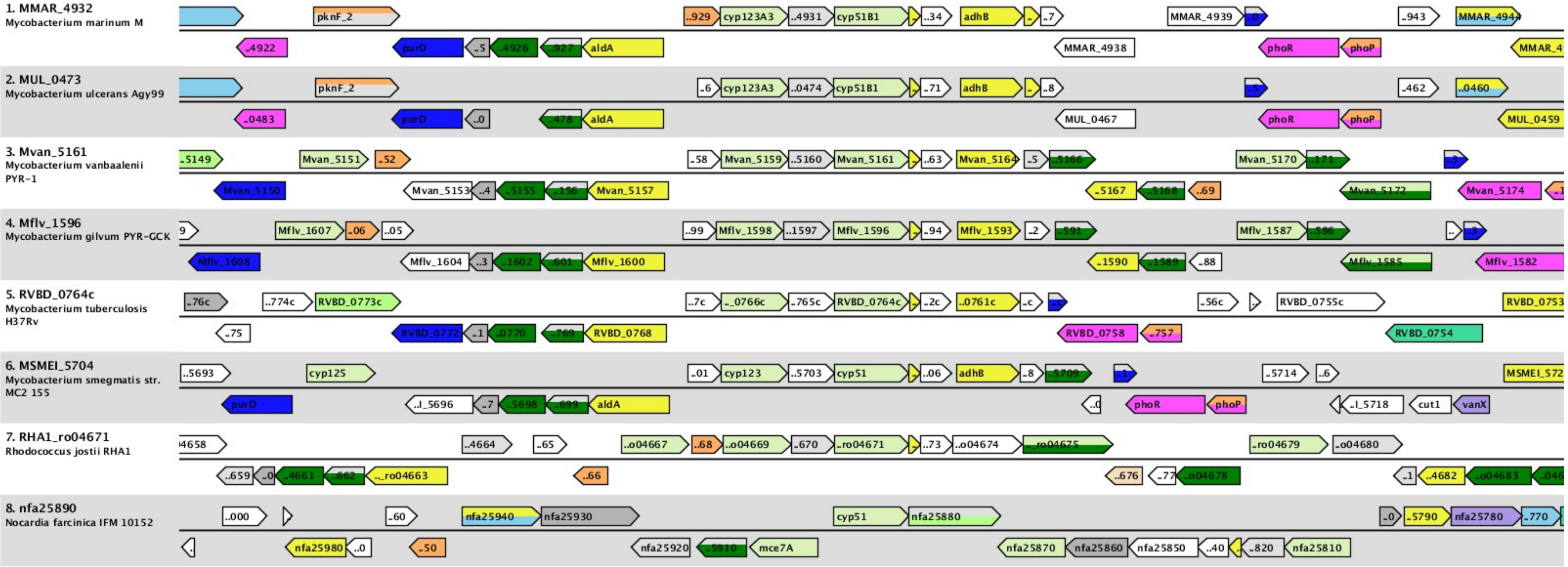

(i) Mmar_4991 (Fdx11, which is not associated with a P450 gene), comparison to *M. liflandii* 128FXT (MulP_05239), *M. ulcerans* Agy99 (Mul_4066), *M. tuberculosis* H37Rv (RVBD_3503c), *M. smegmatis* str. Mc2 155 (MSMEI_5744), *M. kansasii* ATCC 12478 (MKAN_11680), *M. rhodesiae* NB83 (MycrhN_2333) and *M. gilvum* PYR-GCK (Mflv_1596). Many of the surrounding genes are highly conserved across the *Mycobacterium* species.

Mmar_4980 to Mmar_4989 encodes a conserved MCE-associated protein, a conserved hypothetical alanine and valine rich MCE-associated protein, a MCE-family protein Mce4F, a MCE-family lipoprotein LprN, a MCE-family protein Mce4D, a MCE-family protein Mce4C, a MCE-family protein Mce4B, a MCE-family protein Mce4A, a conserved hypothetical membrane protein YrbE4B and a conserved membrane protein YrbE4A, respectively.

Mmar_4990 encodes a short-chain type dehydrogenase/reductase.

Mmar_4992 to Mmar_4996 encodes an acyl-CoA dehydrogenase FadE26, an acyl-CoA dehydrogenase FadE27, a fatty-acid-CoA synthetase FadD17, a PE-PGRS family protein and a conserved protein of unknown function which contains an eta-lactamase/transpeptidase-like superfamily domain

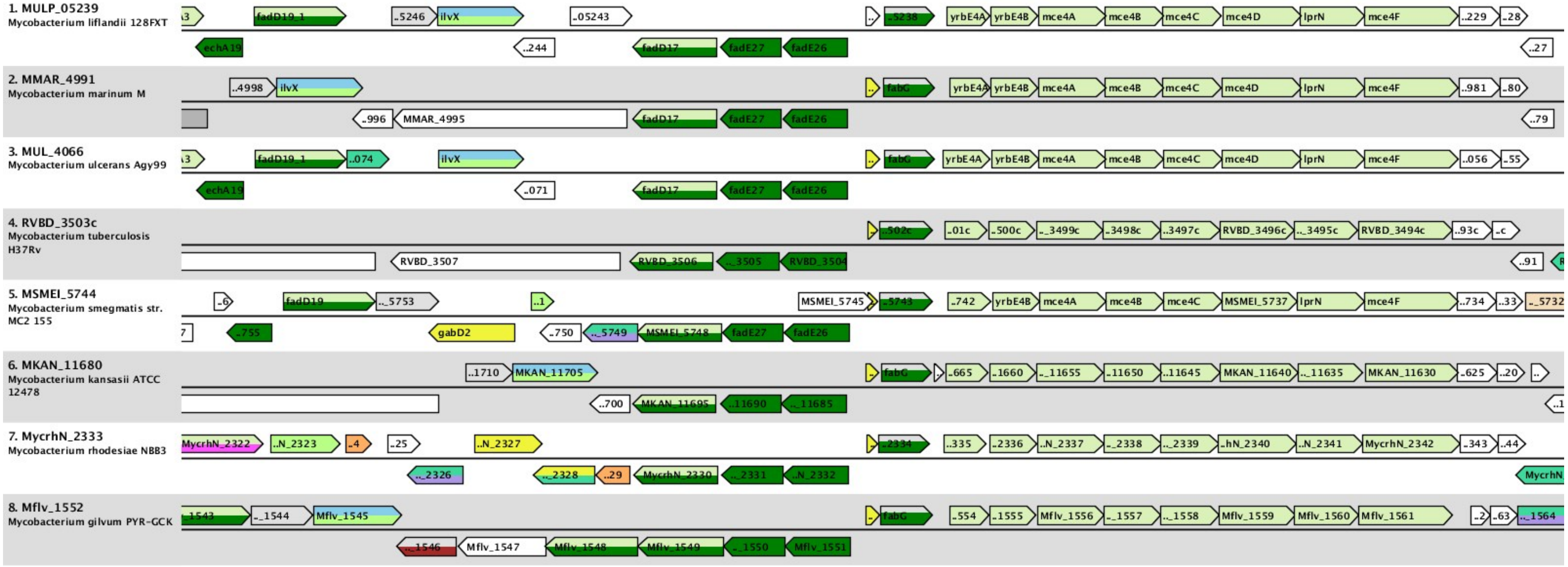

Figure S4 (a) Cell pellet of *E. coli* containing pET26CYP147G1 showing a red colouration due to the CYP147G1 expression but no blue colouration due to indigo formation. (b) Cell culture of the whole-cell oxidation system consisting of pETDuetFdx3/FdR1 and pRSFDuetFdx3/CYP147G1 and the cell pellet showing the formation of a blue dye. (c) Reaction showing how the P450 catalysed oxidation of indole can generate indigo. (d) Pellets and organic extracts of whole-cell turnovers of CYP147G1 with combinations of different electron transfer partners. Only the combination with FdR1 and Fdx3 (Mmar_2931 and Mmar_2932) generated any indigo as detected by formation of a blue colour. In each instance this is pellet and extract on the right hand side of each figure.

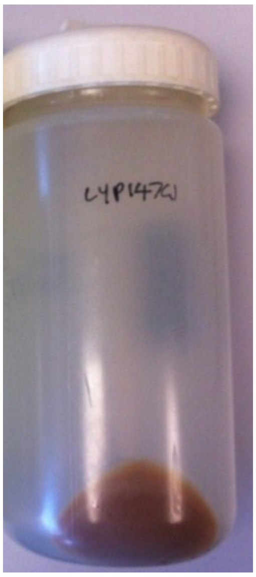

(a)

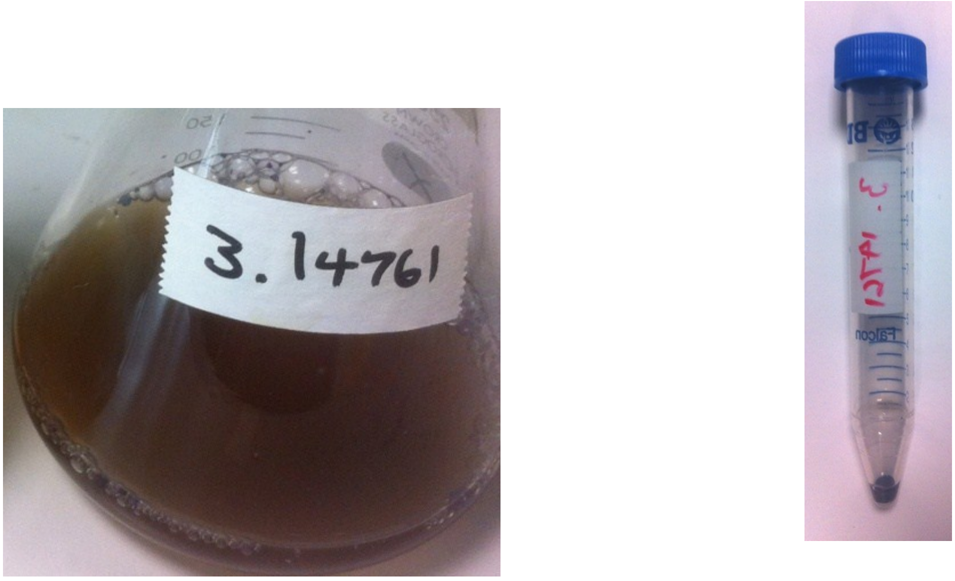

(b)

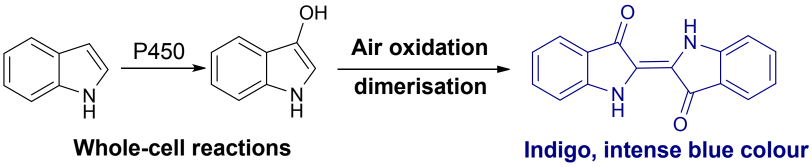

(c)

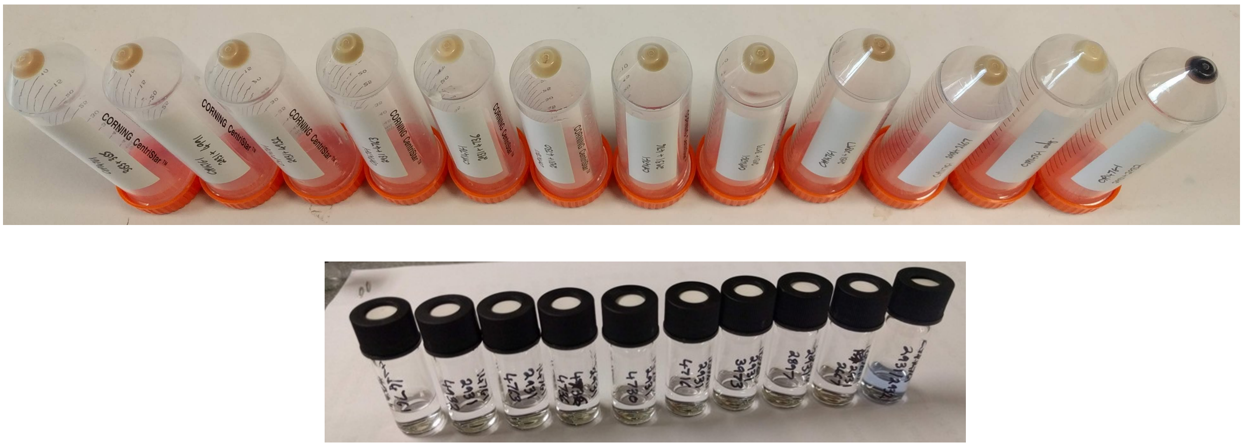

(d)

Figure S4 (e) Pellets and organic extracts of whole-cell turnovers of CYP147G1 with FdR1 and Fdx3 (Mmar_2931 and Mmar_2932) and mutants of Fdx3. In each case from left to right the samples are the tyrosine to cysteine mutant of Fdx3, the tyrosine to glycine mutant of Fdx3, wild-type Fdx3 and a control containing a different ferredoxin (see Fig. S4(d)). The level of indigo formation was greatest for the wild-type enzyme while more indigo was generated in the mutant forms of Fdx3 than in the control.

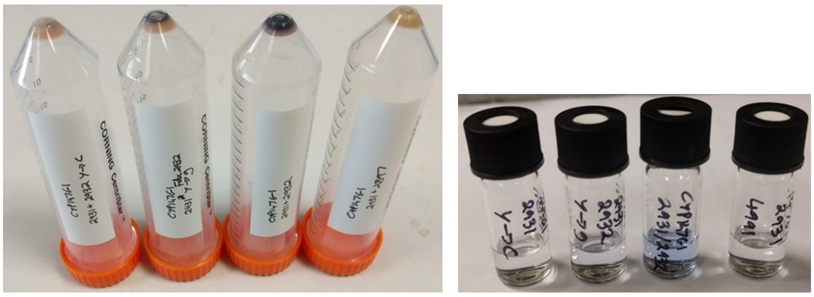

(e)

Figure S5 The spectra of the ferrous, carbon monoxide bound forms of (a) CYP147G1, (b) CYP278A1, (c) CYP269A1, (d) CYP269A1 in the presence of miconazole, (e) CYP150A5 and (f) CYP105Q4. With the exception of CYP269A1 approximately 95% of the proteins shifted from 418 nm (black trace, ferric state) to 450 nm (red trace, reduced CO-bound). In (d), the addition of miconazole to CYP269A1 (black trace before addition, green trace after) stabilised the reduced CO-bound form of the enzyme (blue is reduced state, red is ferrous CO-bound).

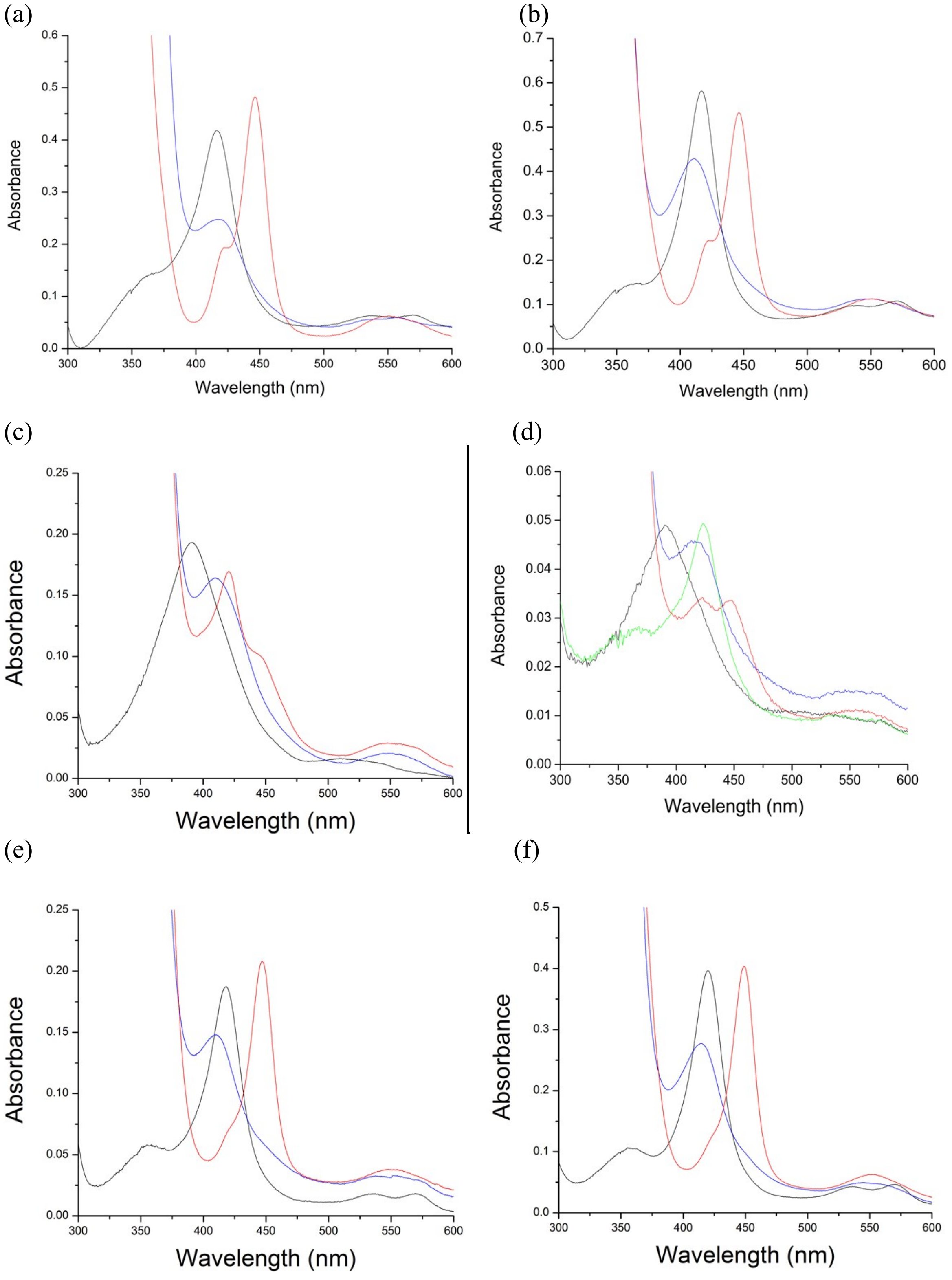

The absorbances of the ferric, ferrous and ferrous-CO bound forms and the extinction coefficients are provided overleaf.

Absorbance maxina of the Soret band for the ferric, ferrous and ferrous-CO species of each P450 enzyme:

CYP147G1 420 nm, 420 nm and 446 nm

CYP278A1 418 nm, 411 nm and 446 nm

CYP269A1 392 nm, 410 nm and 423/446 nm split peak

CYP150A5 418 nm, 409 nm and 447 nm

CYP105Q4 420 nm, 415 nm and 449 nm

Extinction coefficients for the P450s (calculated on CO difference spectra and confirmed by the Pyridine hemochromagen method) * CYP269A1 was only determined by the pyridine hemochromagen method.

CYP147G1

ε_{417}= 111 mM cm^{-1}

CYP150A5

ε_{418}= 103 mM cm^{-1}

CYP278A1

ε_{417}= 126 mM cm^{-1}

CYP269A1*

ε_{392}= 114 mM cm^{-1}

CYP105Q4

ε_{420}= 110 mM cm^{-1}

Figure S6 Spin-state shift and binding constants assays for (a) CYP147G1 with undecanoic acid, (b) CYP278A1 with β-ionone and (c) CYP150A5 with β-ionone.

(a)

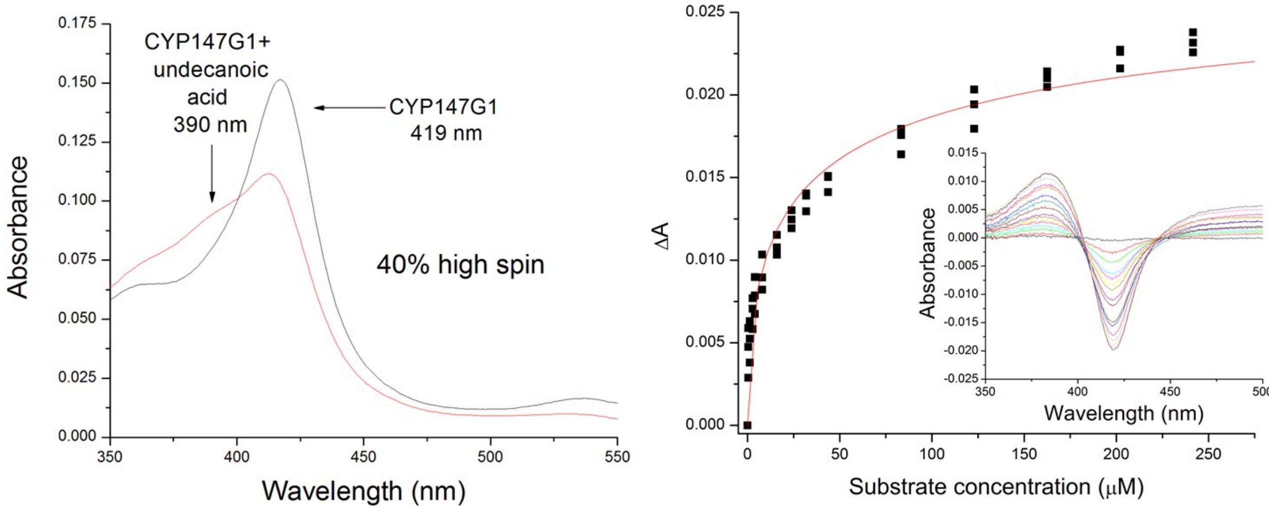

(b)

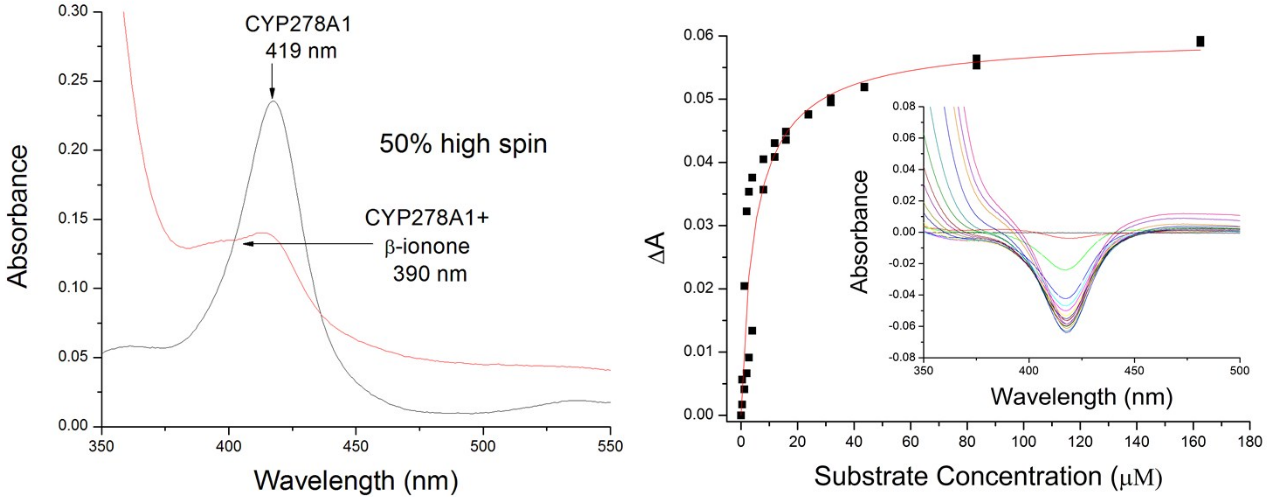

(c)

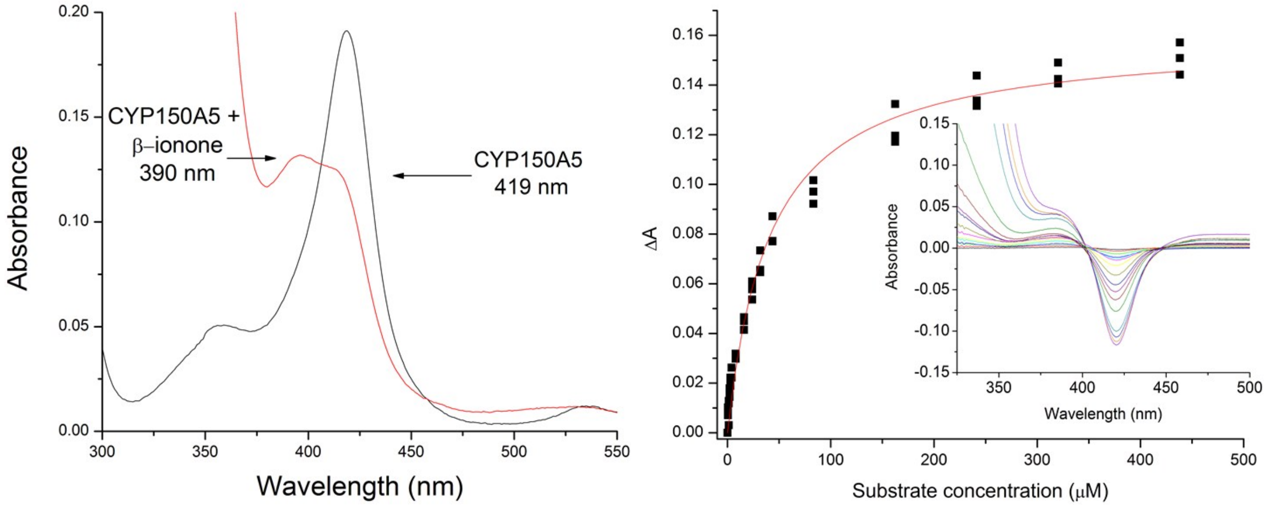

Figure S7 Mass spectrum of GC peaks from CYP147G1 turnovers with undecanoic acid (A) undecanoic acid substrate (retention time 9.2 min), (B) hydroxyundecanoic acid (13.9 min).

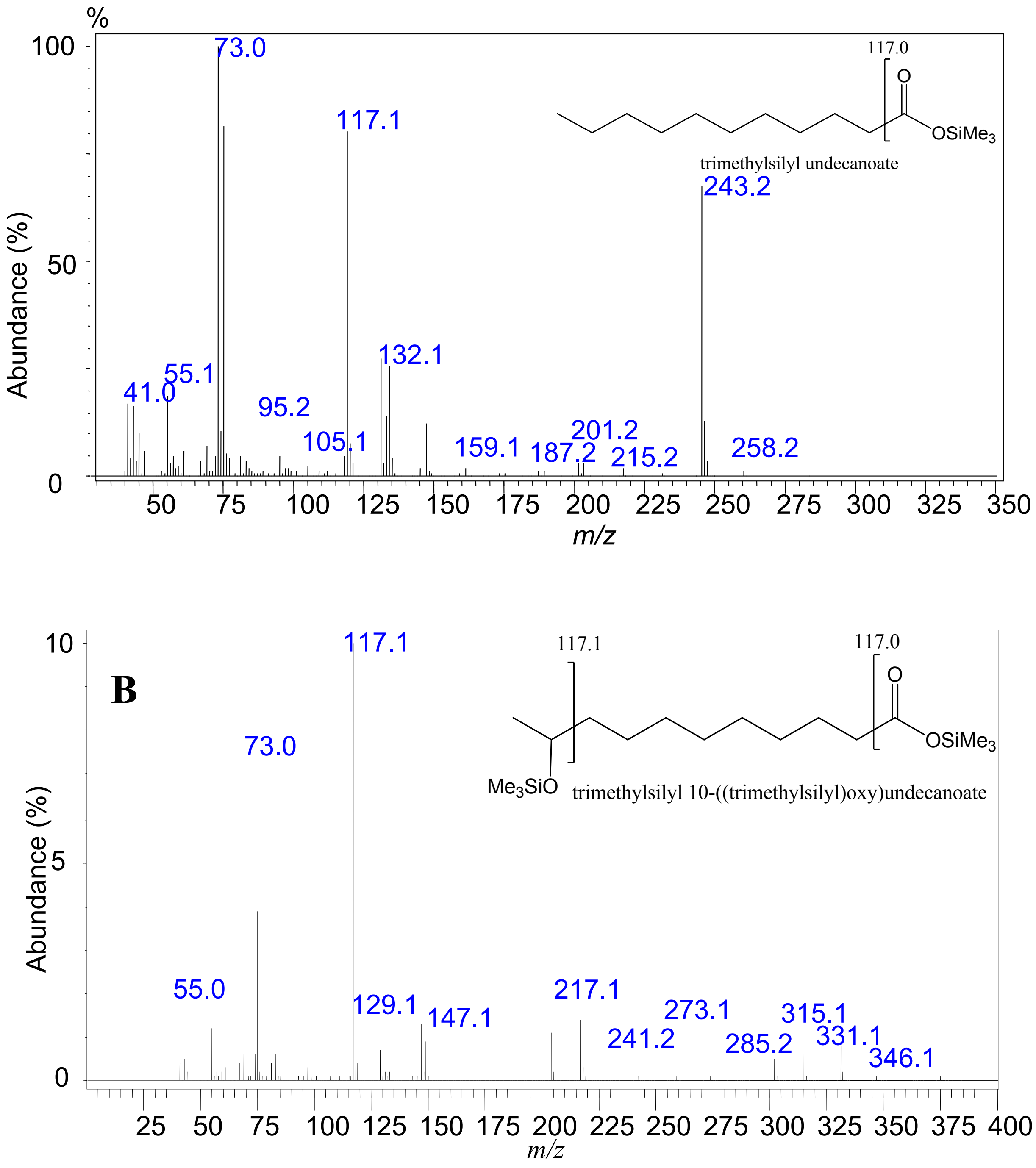

The peak at 9.2 min showed an *m/z* peak at 243.2 in comparison to the expected *m/z* of 258.2 for trimethylsilyl undecanoic acid (a mass loss of 15 *m/z*) and coeluted with the substrate standard (Appendix D). The product of the undecanoic acid turnover (with two trimethylsilyl protecting groups) has an expected *m/z* of 346.24. The peak at 13.9 min showed an *m/z* of 346.1 and a secondary peak of 331.1 (a mass difference of 15 *m/z* from the apparent molecular ion peak).

10-hydroxyundecanoic acid NMR data.^{1}H NMR (500 MHz, $CDCl_3$) δ 3.80 (tq, J = 6.3 Hz, 1H), 2.35 (t, J = 7.4 Hz, 2H), 1.64 (tt, J = 7.4 Hz, 2H), 1.46 – 1.23 (m, 12H), 1.19 (d, J = 6.2 Hz, 3H). ^{13}C NMR (126 MHz, $CDCl_3$) δ 136.81, 132.71, 68.17, 39.26, 33.66, 31.58, 30.26, 29.66, 29.63, 29.45, 29.27, 29.06, 28.95, 25.63, 24.64, 23.45, 19.18.

The overlapping triplet of quartets (tq) at approximately δ 3.8 ppm is distinctive for ω-1 hydroxylation. If the acid was hydroxylated at any other sub-terminal position, the H on the hydroxylated carbon would be a triplet of triplets, as it would not be split by the terminal CH_3 group. Terminal hydroxylation would also be distinctive, as the C**H**$_2$OH group signal would be observed as a triplet of 2H intensity. The 3H doublet peak at δ 1.19 ppm can be assigned to the terminal CH_3 group, split by the single H on the CHOH group, thereby confirming the formation of 10-hydroxyundecanoic acid.

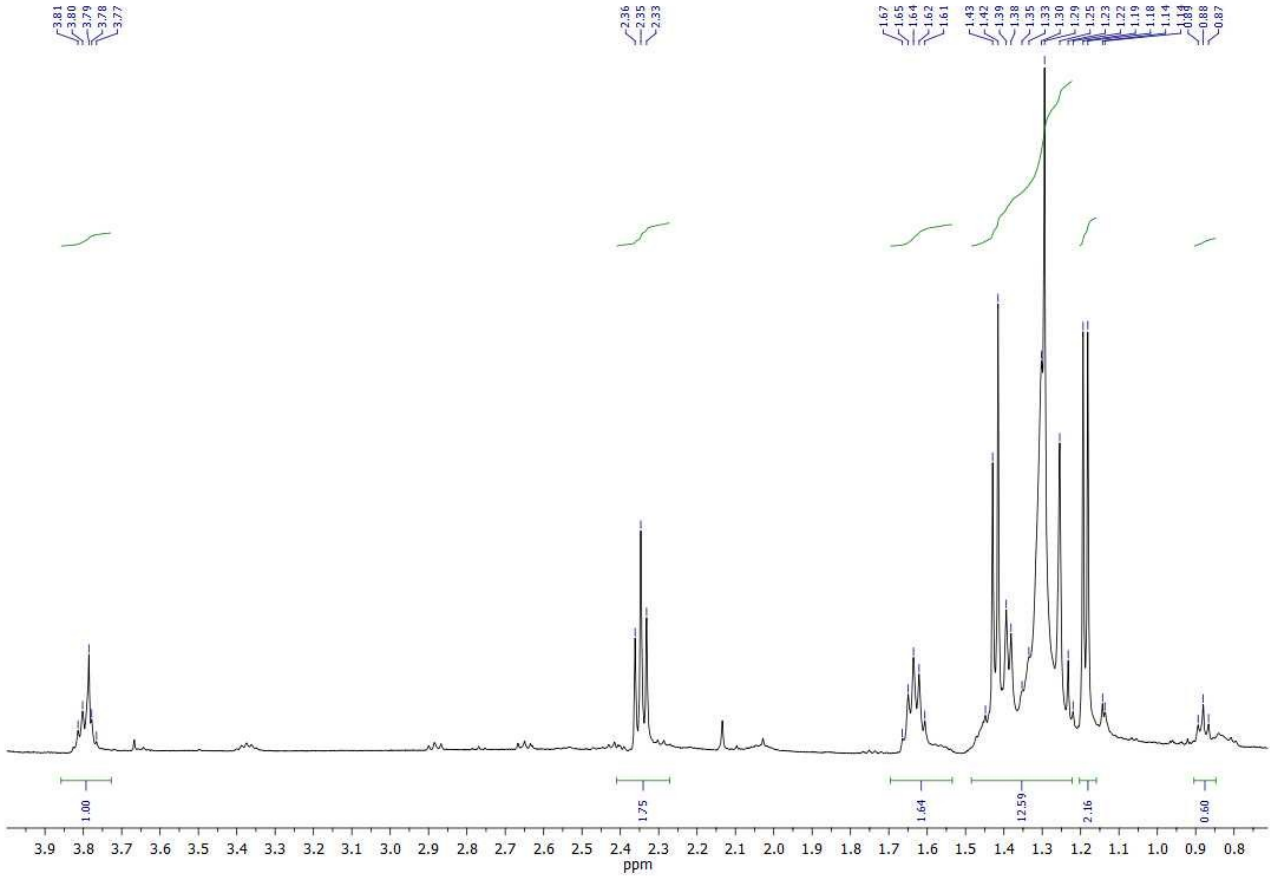

^{1}H NMR of 10-hydroxyundecanoic acid

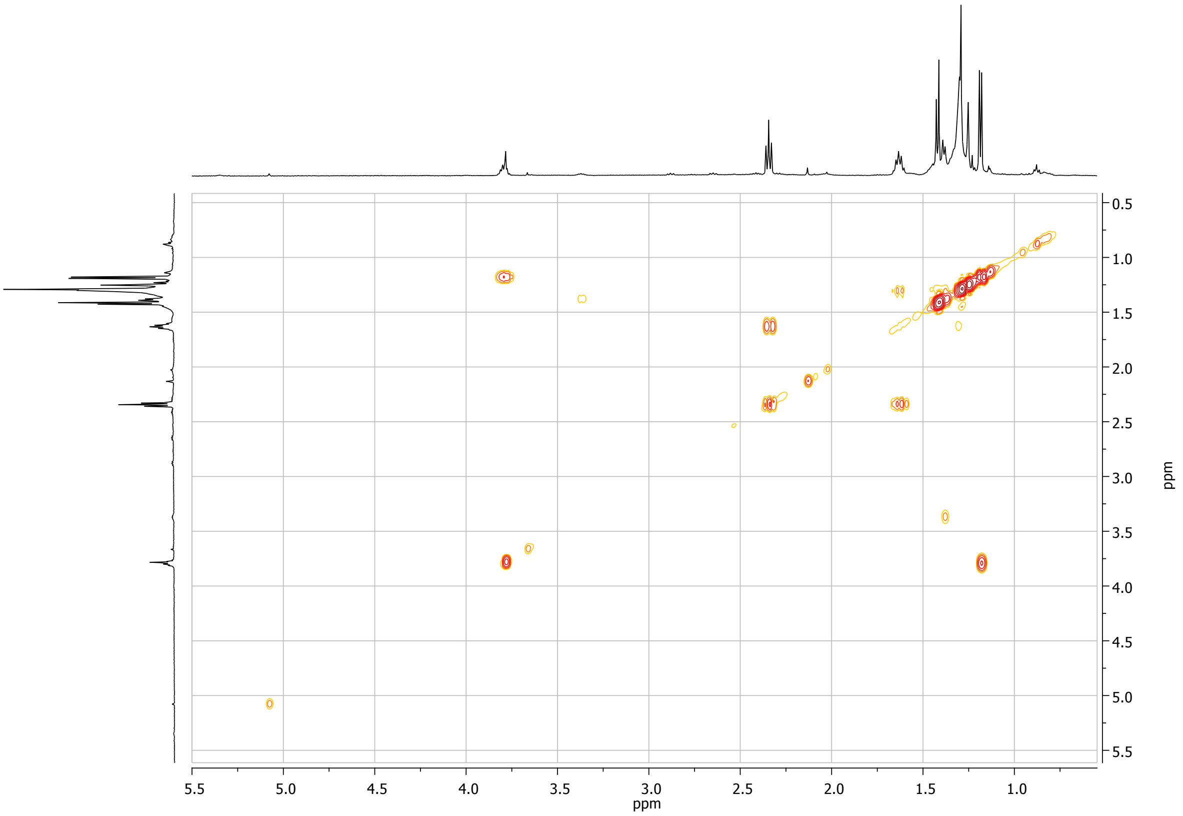

^{1}H COSY NMR of 10-undecanoic acid

Figure S8 Excerpts of sequence chromatograms for Fdx3 mutants, with the successful codon change highlighted in blue.

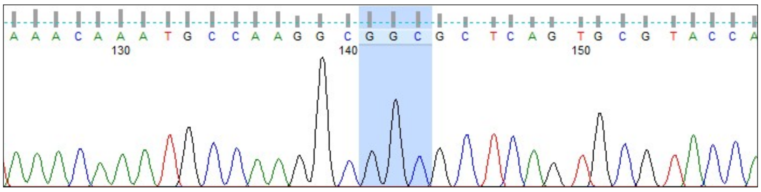

pETDuet with FdR2931 (FdR1) and Fdx2932 (Fdx3) Tyr12Gly – with codon GGC replacing TAC

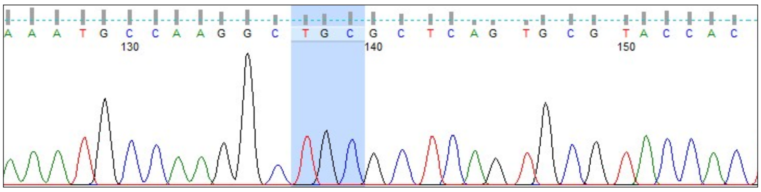

pRSFDuet with CYP147G1 and Fdx2932 (Fdx3) Tyr12Cys – with codon TGC replacing TAC

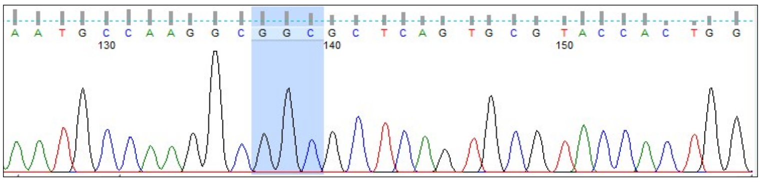

pRSFDuet with CYP147G1 and Fdx2932 (Fdx3) Tyr12Gly – with codon GGC replacing TAC

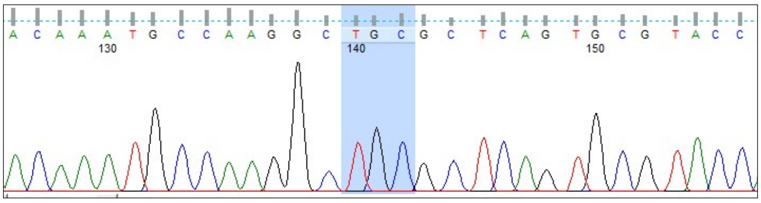

pETDuet with Fdr2931 (FdR1) and Fdx2932 (Fdx3) Try12Cys – with codon TGC replacing TAC

Figure S9 GC-MS chromatograms of the CYP147G1 whole-cell turnovers of undecanoic acid supported by different ferredoxins/electron transfer partners. The samples were derivatised with BSTFA/TMSCl. Derivatised 10-hydroxyundecanoic acid (RT 13.75 min) are shown. In all cases CYP147G1 was produced using the pRSFDuetCYP147G1 vector. In black (Fdx2932) is the turnover supported by pETDuetFdR1/Fdx3 the other chromatograms show different systems where the ferredoxin or both electron transfer partners have been substituted and show that no detectable product was observed. pETFdR2/2Fe-2S (3153/3155 red), pETFdR1/Fdx4 (Fdx3973 blue), pETFdR1/Fdx5 (Fdx4716 magenta) and pETFdR1/Fdx9 (Fdx4763 purple). Other electron transfer partners were also tested and are included in the table below. None of these generated any detectable product.

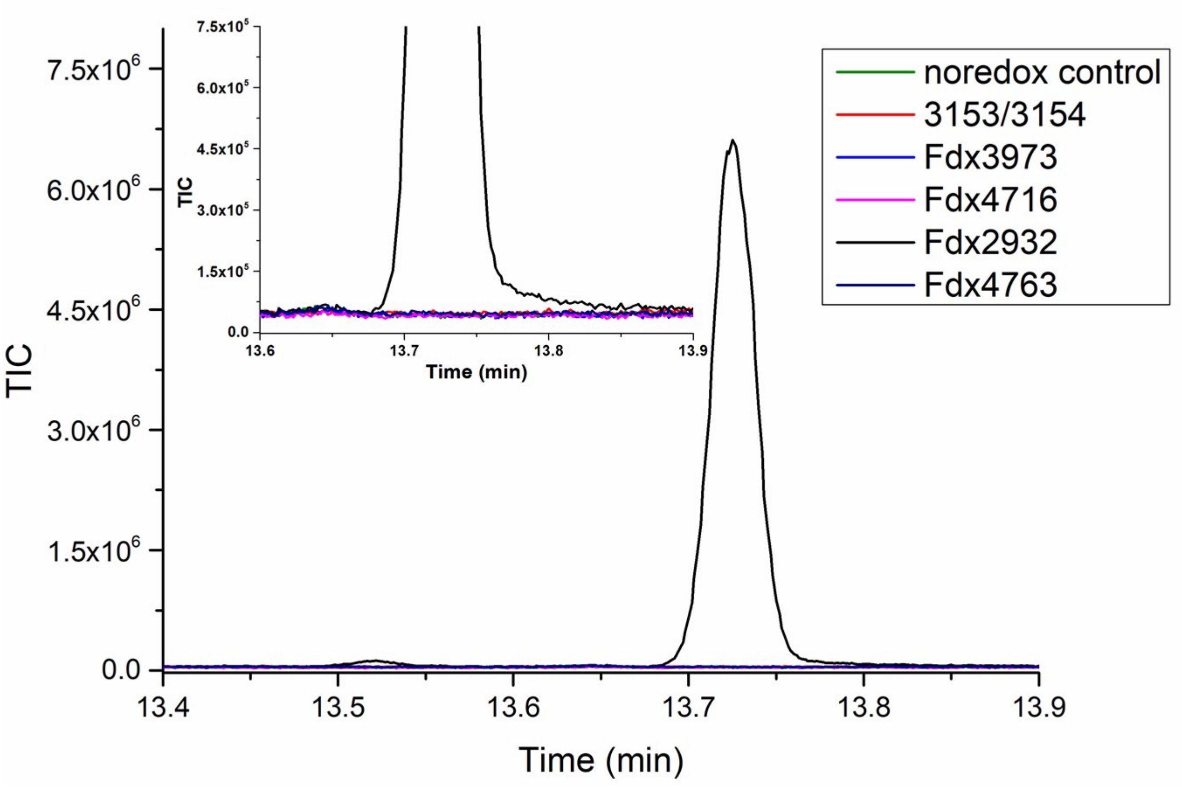

Reductase	Ferredoxin	Ferredoxin type	Source organism
FdR1	Fdx1-Fdx11	[3Fe-4S]	*M. marinum*
FdR2	Mmar_3155	[2Fe-2S]	*M. marinum*
AdR	Adx	[2Fe-2S]	bovine mitochondria[32]
pp1957	pp1957	FMN-[2Fe-2S] fusion	*Pseudomonas putida* KT2440[33]
ArR	Arx	[2Fe-2S]	*Novosphingobium aromaticivorans*[34, 35]
ArR	Tdx	[2Fe-2S]	*N. aromaticivorans* and *Pseudomonas* sp.[36, 37]

Figure S10 Type II spin-state shift and binding constants assays for CYP269A1 with miconazole.

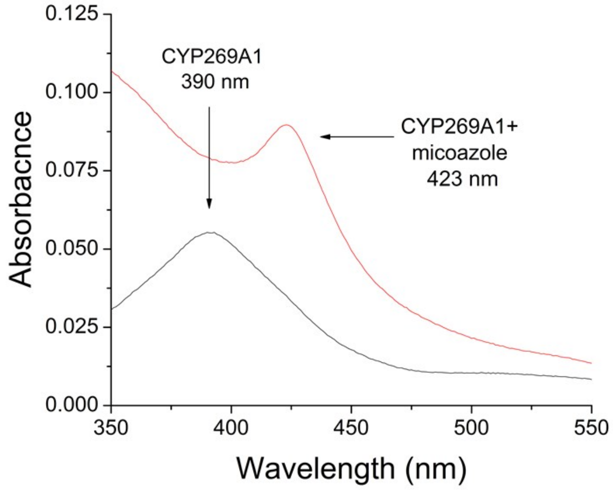

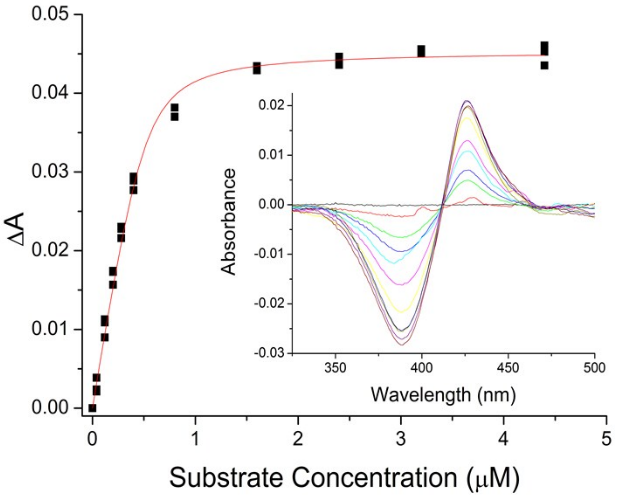

Figure S11 (a) SDS page of ferredoxin proteins. Note that the small highly acidic ferredoxins are difficult to quantitate in terms of their molecular weight via SDS page (Note; 3155 is the 2Fe-2S containing ferredoxin encoded by the gene mMar_3155).[38]

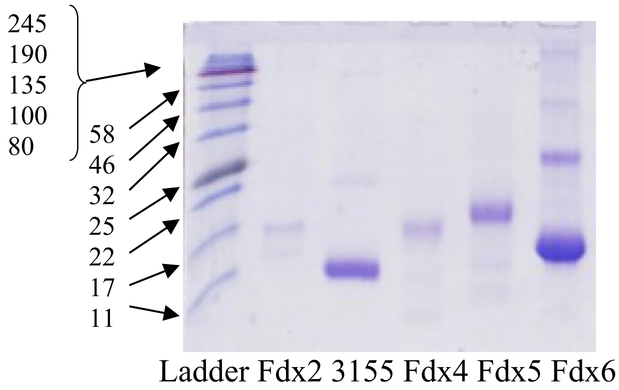

Protein MS data of selected ferredoxins
The average mass of the proteins was measured to be:
2Fe-2S (Mmar_3155)
12093.13 Da (theoretical average mass, 12216.65 Da)
Fdx4 (Mmar_3973)
7647.67 Da (theoretical average mass, 7642.71 Da)
Fdx5 (Mmar_4716)
8288 Da (theoretical average mass, 8118.77 Da)
Fdx6 (Mmar_4730)
11817.81 Da (theoretical average mass, 11781.52 Da)
Fdx8 (Mmar_4736)
7736.88 Da (theoretical average mass 7764.57 Da)
Fdx9 (Mmar_4763)
7662 Da (theoretical average mass, 7657.71 Da)

Note terminal methionine cleavage (-131) was observed in the [2Fe-2S] ferredoxin and Fdx4.

Figure S11 (b) SDS page analysis of Fdx4 purified by IMAC or Ion exchange followed by size exclusion (S100)

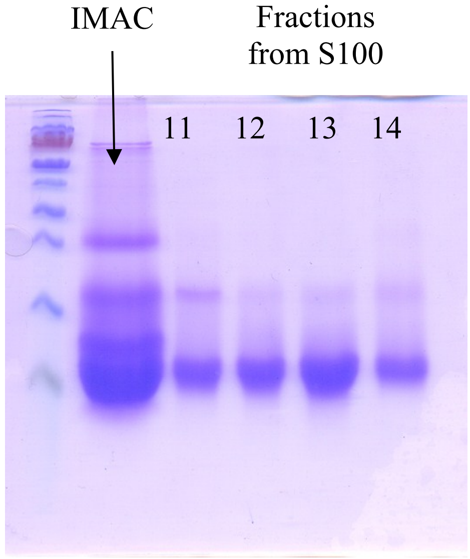

Figure S11 (c) SDS page analysis of associated CYP proteins.

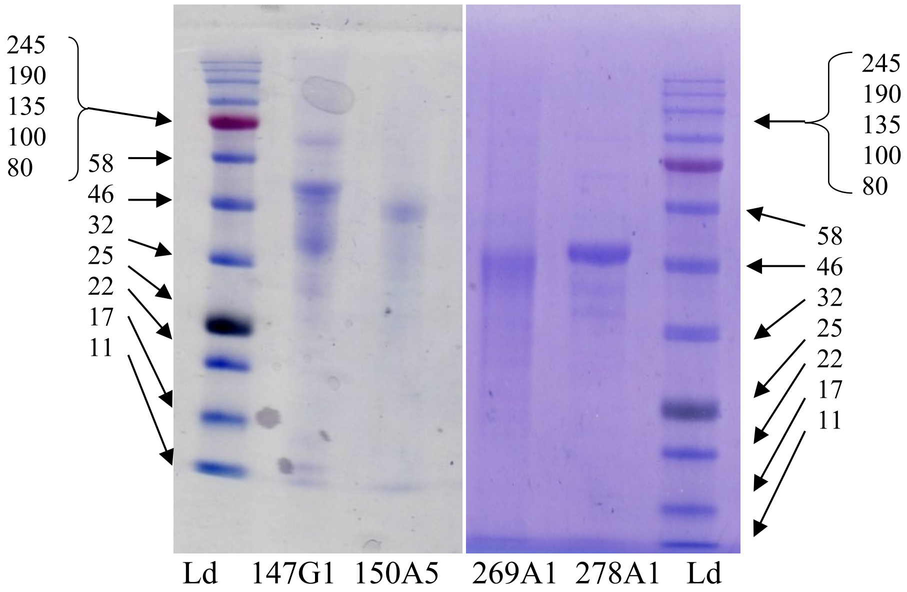

Figure S12 Activity of CYP150A5 and CYP278A1 when supported by native electron transfer partners *in vivo* **(a)** GC analysis of the whole-cell oxidation turnover of CYP150A5 and β-ionone: blue, β-ionone control (RT 12.3 min); magenta, CYP150A5 turnover supported by FdR1 and Fdx8 (product RT 14.3 min); red, *in vitro* turnover of β-ionone by CYP101B1 (major product 3-hydroxy-β-ionone, RT 14.4 min; minor product 4-hydroxy-β-ionone, RT 14.3min) and black, *in vitro* turnover of P450Bm3 (CYP102A1) and β-ionone (sole product, 4-hydroxy-β-ionone). (b) Reverse phase HPLC analysis of the whole-cell turnover of CYP278A1 and β-ionone (black, RT 14.5 min): red, β-ionone control (RT 23.4 min). The chromatograms have been offset along the x- and y-axes for clarity.

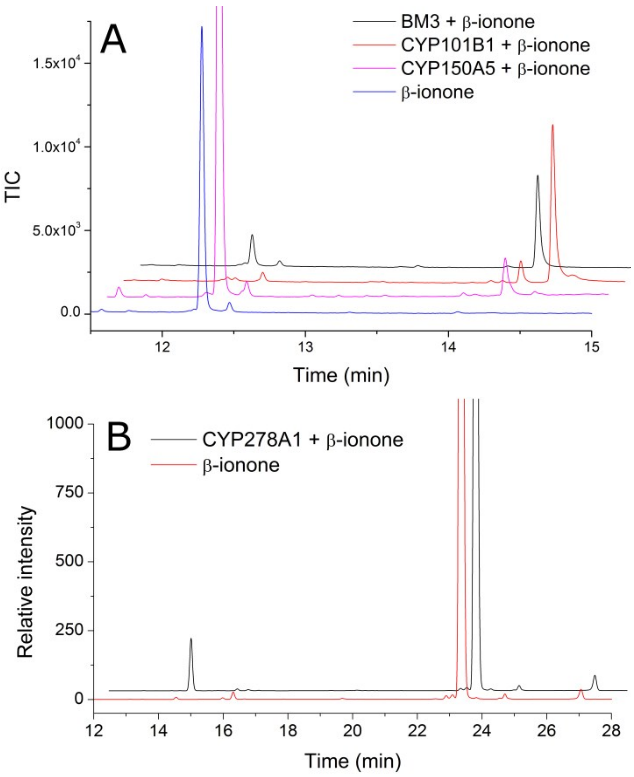

Figure S13 Absorbance (upper) and CD (lower) spectra of Fdx4 (panels A and D), Fdx5 (panels B and E) and Fdx9 (panels C and F) as isolated (red traces), incubated with ferricyanide (black traces), incubated with ascorbate (blue traces) and Eu^{2+} (dark cyan traces).

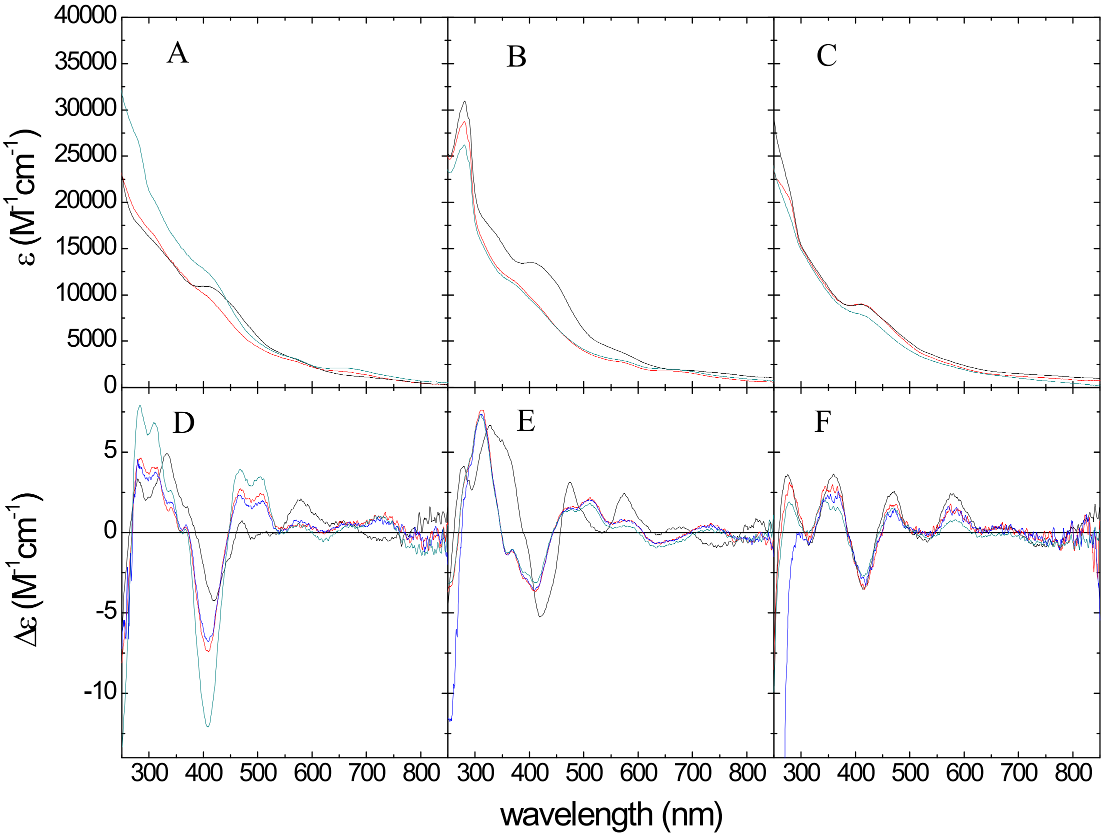

Figure S14 Cluster identification by mass spectrometry. Native (upper) and LC (lower) mass spectra of Fdx4 (panel A), Fdx5 (panel B) and Fdx9 (panel C). Each of the LC-MS spectra contain a peak at the predicted mass of the apo protein and an additional peak 131 Da lighter than this, consistent with cleavage of the N-terminal methionine. The native mass spectra contain peaks 296 Da higher in mass than each of these features (indicated by red arrows) consistent with binding of a [3Fe-4S] cluster by each of the ferredoxins.

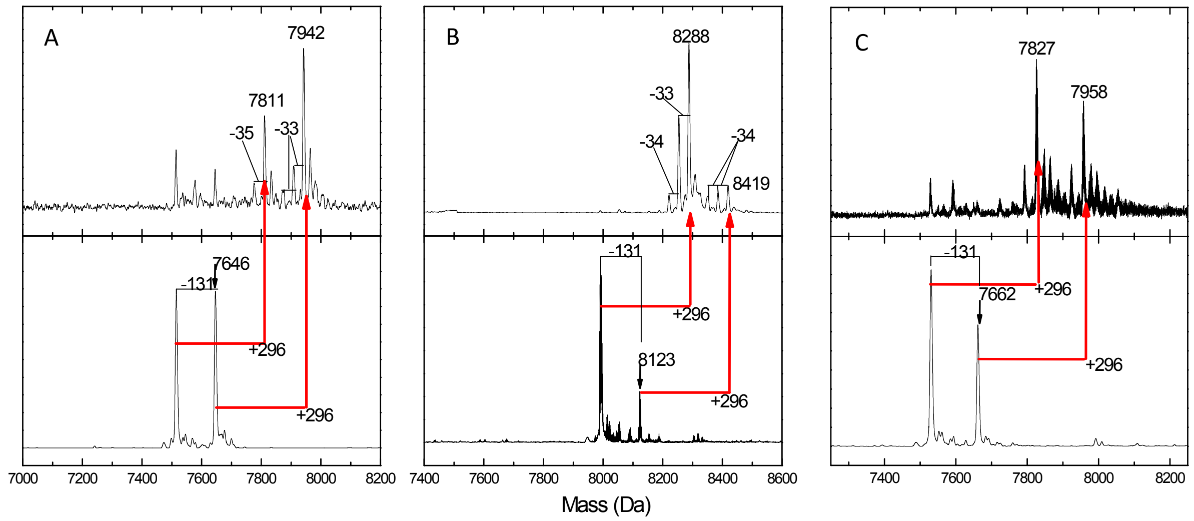

Figure S15 EPR of spectroscopy of Fdx4 (Panel A), Fdx5 (Panel B) and Fdx9 (Panel C). The form of the spectra was consistent with the oxidised form of a [3Fe-4S] cluster in each case. The black traces are those recorded for the proteins as isolated, red traces those following incubation with ferricyanide and blue traces those following incubation with Eu^{2+}.

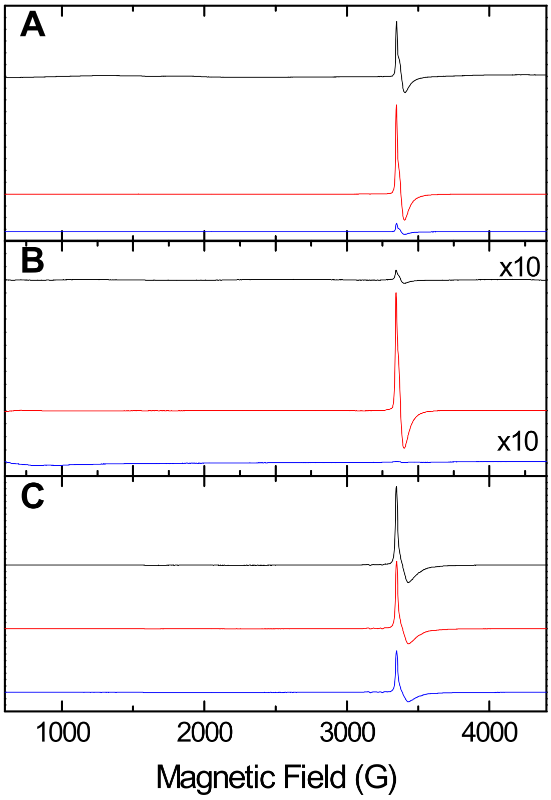

Figure S16 CD Spectra of Fdx4 isolated utilising a Ni^{2+} charged IMAC column (black trace) or a combination of anion exchange and size exclusion chromatography (red trace). Blue trace represents the latter sample following incubation with 1.0 stoichiometric equivalent of $(NH_4)_2Fe(SO_4)_2$

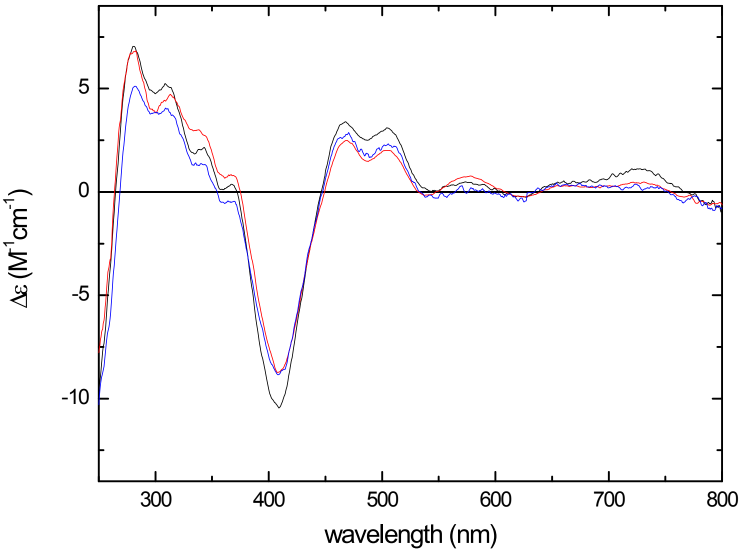

Figure S17 Measurement of the UV spectra (absorbance at 406 nm) of Fdx2 and Fdx4 which were purified under anaerobic conditions and then exposed to dioxygen.

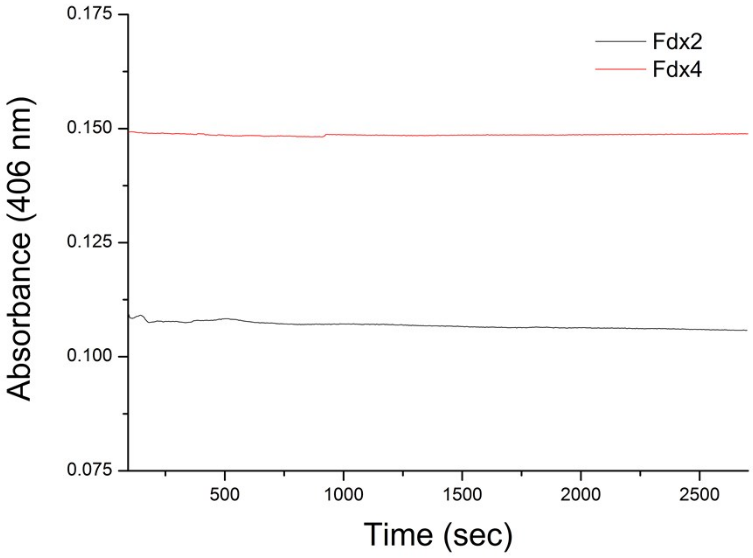

References

1. S. Rupasinghe, M. A. Schuler, N. Kagawa, H. Yuan, L. Lei, B. Zhao, S. L. Kelly, M. R. Waterman and D. C. Lamb, *FEBS Letters*, 2006, **580**, 6338-6342.
2. T. L. Poulos, *Chem. Rev.*, 2014, **114**, 3919-3962.
3. J. P. Clark, C. S. Miles, C. G. Mowat, M. D. Walkinshaw, G. A. Reid, S. N. Daff and S. K. Chapman, *J. Inorg. Biochem.*, 2006, **100**, 1075-1090.
4. A. Bellamine, A. T. Mangla, W. D. Nes and M. R. Waterman, *Proc. Natl. Acad. Sci. U. S. A.*, 1999, **96**, 8937-8942.
5. J. K. Capyk, R. Kalscheuer, G. R. Stewart, J. Liu, H. Kwon, R. Zhao, S. Okamoto, W. R. Jacobs, Jr., L. D. Eltis and W. W. Mohn, *J. Biol. Chem.*, 2009, **284**, 35534-35542.
6. M. D. Driscoll, K. J. McLean, C. Levy, N. Mast, I. A. Pikuleva, P. Lafite, S. E. Rigby, D. Leys and A. W. Munro, *J. Biol. Chem.*, 2010, **285**, 38270-38282.
7. J. B. Johnston, P. M. Kells, L. M. Podust and P. R. Ortiz de Montellano, *Proc. Natl. Acad. Sci. U. S. A.*, 2009, **106**, 20687-20692.
8. J. B. Johnston, H. Ouellet and P. R. Ortiz de Montellano, *J. Biol. Chem.*, 2010, **285**, 36352-36360.
9. D. Scheps, S. H. Malca, H. Hoffmann, B. M. Nestl and B. Hauer, *Org. Biomol. Chem.*, 2011, **9**, 6727-6733.
10. H. Ouellet, L. M. Podust and P. R. de Montellano, *J. Biol. Chem.*, 2008, **283**, 5069-5080.
11. M. D. Driscoll, K. J. McLean, M. R. Cheesman, T. A. Jowitt, M. Howard, P. Carroll, T. Parish and A. W. Munro, *Biochim. Biophys. Acta*, 2011, **1814**, 76-87.
12. B. Brezna, O. Kweon, R. L. Stingley, J. P. Freeman, A. A. Khan, B. Polek, R. C. Jones and C. E. Cerniglia, *Appl. Microbiol. Biotechnol.*, 2006, **71**, 522-532.
13. H. Ouellet, J. B. Johnston and P. R. Ortiz de Montellano, *Arch. Biochem. Biophys.*, 2010, **493**, 82-95.
14. E. Garcia-Fernandez, D. J. Frank, B. Galan, P. M. Kells, L. M. Podust, J. L. Garcia and P. R. Ortiz de Montellano, *Environ. Microbiol.*, 2013, **15**, 2342-2359.
15. T. P. Stinear, A. Mve-Obiang, P. L. Small, W. Frigui, M. J. Pryor, R. Brosch, G. A. Jenkin, P. D. Johnson, J. K. Davies, R. E. Lee, S. Adusumilli, T. Garnier, S. F. Haydock, P. F. Leadlay and S. T. Cole, *Proc. Natl. Acad. Sci. U. S. A.*, 2004, **101**, 1345-1349.
16. P. Belin, M. H. Le Du, A. Fielding, O. Lequin, M. Jacquet, J. B. Charbonnier, A. Lecoq, R. Thai, M. Courcon, C. Masson, C. Dugave, R. Genet, J. L. Pernodet and M. Gondry, *Proc. Natl. Acad. Sci. U. S. A.*, 2009, **106**, 7426-7431.
17. S. G. Bell, N. Hoskins, F. Xu, D. Caprotti, Z. Rao and L. L. Wong, *Biochem. Biophys Res. Commun.*, 2006, **342**, 191-196.
18. S. G. Bell and L. L. Wong, *Biochem. Biophys. Res. Commun.*, 2007, **360**, 666-672.
19. H. Ouellet, S. Guan, J. B. Johnston, E. D. Chow, P. M. Kells, A. L. Burlingame, J. S. Cox, L. M. Podust and P. R. de Montellano, *Mol. Microbiol.*, 2010, **77**, 730-742.
20. A. Zhang, T. Zhang, E. A. Hall, S. Hutchinson, M. J. Cryle, L. L. Wong, W. Zhou and S. G. Bell, *Molecular bioSystems*, 2015, **11**, 869-881.
21. D. R. Nelson, *Methods Mol. Biol.*, 1998, **107**, 15-24.
22. D. R. Nelson, *Methods Mol. Biol.*, 2006, **320**, 1-10.
23. S. T. Cole, R. Brosch, J. Parkhill, T. Garnier, C. Churcher, D. Harris, S. V. Gordon, K. Eiglmeier, S. Gas, C. E. Barry, 3rd, F. Tekaia, K. Badcock, D. Basham, D. Brown, T. Chillingworth, R. Connor, R. Davies, K. Devlin, T. Feltwell, S. Gentles, N. Hamlin, S. Holroyd, T. Hornsby, K. Jagels, A. Krogh, J. McLean, S. Moule, L. Murphy, K. Oliver, J. Osborne, M. A. Quail, M. A. Rajandream, J. Rogers, S. Rutter, K. Seeger, J. Skelton, R. Squares, S. Squares, J. E. Sulston, K. Taylor, S. Whitehead and B. G. Barrell, *Nature*, 1998, **393**, 537-544.
24. T. P. Stinear, T. Seemann, S. Pidot, W. Frigui, G. Reysset, T. Garnier, G. Meurice, D. Simon, C. Bouchier, L. Ma, M. Tichit, J. L. Porter, J. Ryan, P. D. Johnson, J. K. Davies, G. A. Jenkin, P. L. Small, L. M. Jones, F. Tekaia, F. Laval, M. Daffe, J. Parkhill and S. T. Cole, *Genome Res.*, 2007, **17**, 192-200.
25. D. T. Jones, W. R. Taylor and J. M. Thornton, *Comput. Appl. Biosci.*, 1992, **8**, 275-282.
26. K. Tamura, G. Stecher, D. Peterson, A. Filipski and S. Kumar, *Mol. Biol. Evol.*, 2013, **30**, 2725-2729.
27. I. Barr and F. Guo, *Bio. Protoc.*, 2015, **5**.
28. J. W. Williams and J. F. Morrison, *Methods Enzymol.*, 1979, **63**, 437-467.

29. T. Horak, J. Culik, P. Cejka, M. Jurkova, V. Kellner, J. Dvorak and D. Haskova, *J. Agric. Food Chem.*, 2009, **57**, 11081-11085.
30. L. P. Jenner and J. N. Butt, personal communication.
31. L. Overmars, R. Kerkhoven, R. J. Siezen and C. Francke, *BMC genomics*, 2013, **14**, 209.
32. A. V. Grinberg, F. Hannemann, B. Schiffler, J. Muller, U. Heinemann and R. Bernhardt, *Proteins*, 2000, **40**, 590-612.
33. S. G. Bell, L. French, N. H. Rees, S. S. Cheng, G. Preston and L. L. Wong, *Biotechnol. Appl. Biochem.*, 2013, **60**, 9-17.
34. S. G. Bell, A. Dale, N. H. Rees and L. L. Wong, *Appl. Microbiol. Biotechnol.*, 2010, **86**, 163-175.
35. W. Yang, S. G. Bell, H. Wang, W. Zhou, N. Hoskins, A. Dale, M. Bartlam, L. L. Wong and Z. Rao, *J. Biol. Chem.*, 2010, **285**, 27372-27384.
36. S. A. Child, E. F. Naumann, J. B. Bruning and S. G. Bell, *Biochem. J.*, 2018, **475**, 705-722.
37. J. A. Peterson, J. Y. Lu, J. Geisselsoder, S. Graham-Lorence, C. Carmona, F. Witney and M. C. Lorence, *J. Biol. Chem.*, 1992, **267**, 14193-14203.
38. J. G. Williams and W. B. Gratzer, *J. Chromatogr.*, 1971, **57**, 121-125.
